# Scaffolding protein functional sites using deep learning

**DOI:** 10.1126/science.abn2100

**Published:** 2022-07-21

**Authors:** Jue Wang, Sidney Lisanza, David Juergens, Doug Tischer, Joseph L. Watson, Karla M. Castro, Robert Ragotte, Amijai Saragovi, Lukas F. Milles, Minkyung Baek, Ivan Anishchenko, Wei Yang, Derrick R. Hicks, Marc Expòsit, Thomas Schlichthaerle, Jung-Ho Chun, Justas Dauparas, Nathaniel Bennett, Basile I. M. Wicky, Andrew Muenks, Frank DiMaio, Bruno Correia, Sergey Ovchinnikov, David Baker

**Affiliations:** a Department of Biochemistry, University of Washington, Seattle, WA 98105, USA; b Institute for Protein Design, University of Washington, Seattle, WA 98105, USA; c Graduate program in Biological Physics, Structure and Design, University of Washington, Seattle, WA 98105, USA; d FAS Division of Science, Harvard University, Cambridge, MA 02138, USA; e John Harvard Distinguished Science Fellowship Program, Harvard University, Cambridge, MA 02138, USA; f Howard Hughes Medical Institute, University of Washington, Seattle, WA 98105, USA; g Molecular Engineering Graduate Program, University of Washington, Seattle, WA 98105, USA; h Institute of Bioengineering, École Polytechnique Fédérale de Lausanne, Lausanne CH-1015, Switzerland

## Abstract

The binding and catalytic functions of proteins are generally mediated by a small number of functional residues held in place by the overall protein structure. We describe deep learning approaches for scaffolding such functional sites without needing to pre-specify the fold or secondary structure of the scaffold. The first approach, “constrained hallucination”, optimizes sequences such that their predicted structures contain the desired functional site. The second approach, “inpainting”, starts from the functional site and fills in additional sequence and structure to create a viable protein scaffold in a single forward pass through a specifically trained RosettaFold network. We use the methods to design candidate immunogens, receptor traps, metalloproteins, enzymes, and protein-binding proteins, and validate the designs using a combination of in silico and experimental tests.

The biochemical functions of proteins are often carried out by a subset of residues which constitute a functional site--for example, an enzyme active site or a protein or small molecule binding site--and hence the design of proteins with new functions can be divided into two steps. The first step is to identify functional site geometries and amino acid identities which produce the desired activity--for enzymes this can be done using quantum chemistry calculations (1–3) and for protein binders by fragment docking calculations (4, 5); alternatively, functional sites can be extracted from a native protein having the desired activity (6, 7). In this paper, we focus on the second step: given a functional site description from any source, design an amino acid sequence which folds up to a three dimensional structure containing the site. Previous methods can scaffold functional sites made up of one or two contiguous chain segments (6–10), but with the exception of helical bundles (8) these do not extend readily to more complex sites composed of three or more chain segments, and the generated backbones are not guaranteed to be designable (encodable by some amino acid sequence).

An ideal method for functional de novo protein design would 1) embed the functional site with minimal distortion in a designable scaffold protein; 2) be applicable to arbitrary site geometries, searching over all possible scaffold topologies and secondary structure compositions for those optimal for harboring the specified site, and 3) jointly generate backbone structure and amino acid sequence. We previously demonstrated that the trRosetta structure-prediction neural network (11) can be used to generate new proteins by maximizing the trRosetta output probability that a sequence folds to some (unspecified) three dimensional structure during Monte Carlo sampling in sequence space (12). We refer to this process as “hallucination” as it produces solutions that the network considers ideal proteins but do not correspond to any known natural protein; crystal and NMR structures confirm that the hallucinated sequences fold to the hallucinated structures (12). trRosetta can also be used to design sequences that fold into a target backbone structure by carrying out sequence optimization using a structure recapitulation loss function that rewards similarity of the predicted structure to the target structure (13). Given this ability to design both sequence and structure, we reasoned that trRosetta could be adapted to tackle the functional site scaffolding problem.

## Partially constrained hallucination using a multi-objective loss function

**Figure 1.**
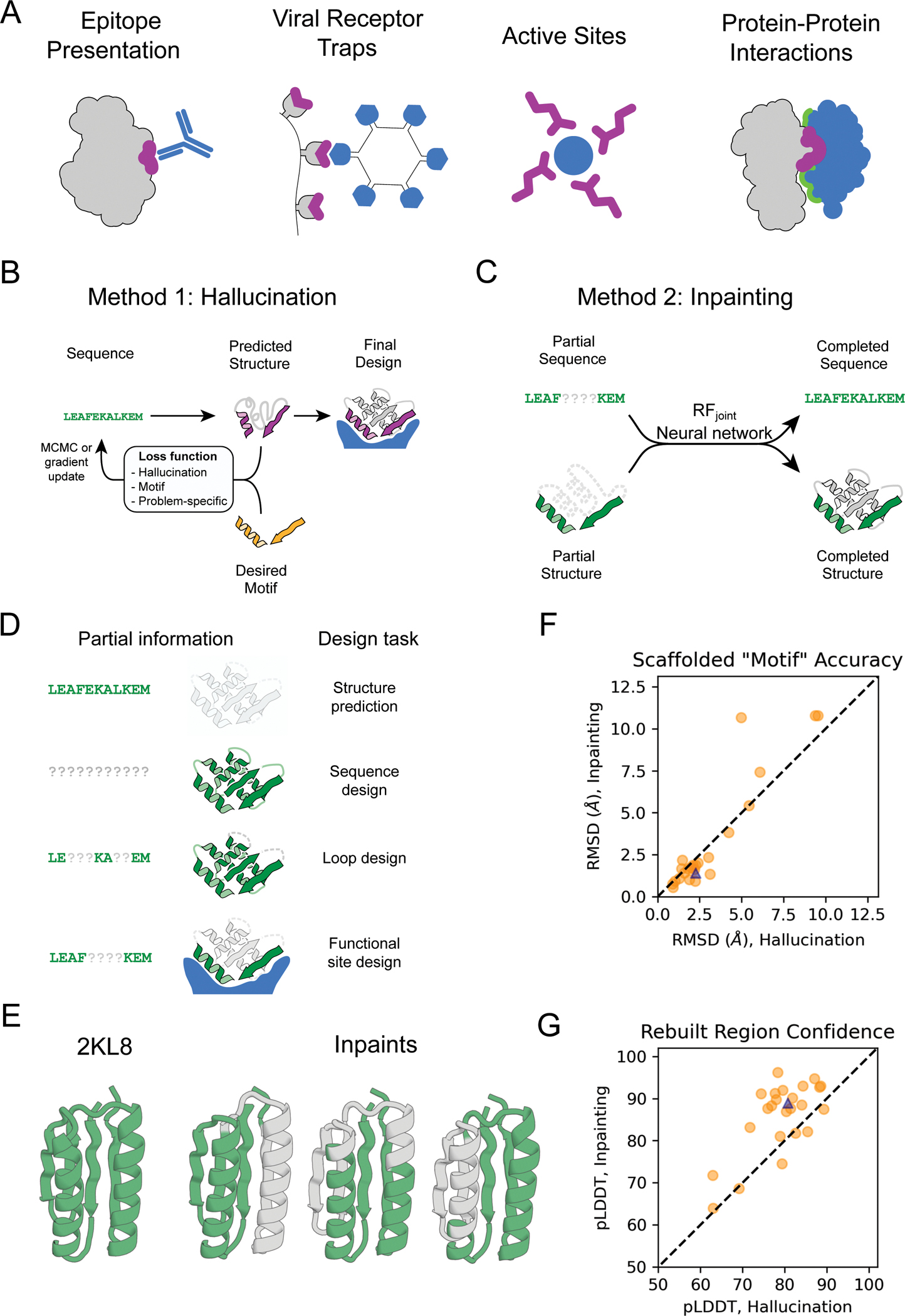
Methods for protein function design (A) Applications of functional-site scaffolding. (B-C) Design methods. (B) Constrained hallucination. At each iteration, a sequence is passed to the trRosetta or RosettaFold neural network, which predicts 3D coordinates and residue-residue distances and orientations (Fig. S2) which are scored by a loss function that rewards certainty of the predicted structure along with motif recapitulation and other task-specific functions. (C) Missing information recovery (“Inpainting”). Partial sequence and/or structural information is input into a modified RosettaFold network (termed RF_*joint*_), and complete sequence and structure are output. (D) Protein design challenges formulated as missing information recovery problems. (E) Joint RosettaFold (RF_*joint*_) can simultaneously recover structure and sequence of a masked region of protein. 2KL8 was fed into RF_*joint*_ with a continuous (length 30) window of sequence and structure masked out, with the network tasked with predicting the missing region of protein. Outputs (inpainted region in gray) closely resemble the original protein (2KL8, left) and are confidently predicted by AlphaFold (pLDDT/Motif RMSD of models shown: 91.6/0.91, 92.0/0.69, 90.4/0.82 respectively). (F-G) Motif scaffolding benchmarking data comparing RF_*joint*_ with constrained hallucination. A set of 28 *de novo* designed proteins, published since RosettaFold was trained, were used. For each protein, 20 random masks of length 30 were generated, and RF_*joint*_ and hallucination were tasked with filling in the missing sequence and structure to “scaffold” the unmasked “Motif”. For this mask length, RF_*joint*_ typically modestly outperforms hallucination, both in terms of the RMSD of the unmasked protein (the “motif”) to the original structure (F), and in AlphaFold confidence (pLDDT in the replaced region) (G). Circles: Average of 20 outputs for each of the benchmarking proteins. Triangle: 2KL8. Colors in all panels: native functional motif (orange); hallucinated/inpainted scaffold (gray); constrained motif (purple); binding partner (blue); non-masked region (green); masked region (light gray, dotted lines).

To extend existing trRosetta-based design methods to scaffold functional sites (Fig. 1A), we optimized amino acid sequences for folding to a structure containing the desired functional site using a composite loss function that combines the previously used hallucination loss with a motif reconstruction loss over the functional motif (rather than the entire structure as in (13) (Fig. 1B; Methods). While we succeeded in generating structures with segments closely recapitulating functional sites, Rosetta structure predictions suggested that the sequences poorly encoded the structures (Fig. S1A), and hence we used Rosetta design calculations to generate more-optimal sequences (14). Several designs targeting PD-L1 generated by constrained hallucination with binding motifs derived from PD-1 (Table S1) (15), followed by Rosetta design, were found to have binding affinities in the mid-nanomolar range (Fig. S1B–E). While this experimental validation is encouraging, the requirement for sequence design using Rosetta is inconsistent with the aim of jointly designing sequence and structure.

Following the development of RosettaFold (RF) (16) we found that it performed better than trRosetta in guiding protein design by functional-site-constrained hallucination (Fig. S1G), likely reflecting the better overall modeling of protein sequence-structure relationships (16). Constrained hallucination with RosettaFold has the further advantages that because 3D coordinates are explicitly modeled (trRosetta only generates residue-residue distances and orientations), site recapitulation can be assessed at the coordinate level, and additional problem-specific loss terms can be implemented in coordinate space that assess interactions with a target (Fig. S2; Materials and Methods).

## Generalized functional motif scaffolding by missing information recovery

While powerful and general, the constrained hallucination approach is compute-intensive, as a forward and backward pass through the network is required for each gradient descent step during sequence optimization. In the training of recent versions of RosettaFold, a subset of positions in the input multiple sequence alignment (MSA) are masked and the network is trained to recover this missing sequence information in addition to predicting structure. This ability to recover both sequence and structural information provides a second solution to the functional site scaffolding problem: given a functional site description, a forward pass through the network can be used to complete, or “inpaint”, both protein sequence and structure in a missing/masked region of protein (Fig. 1C; Methods). Here, the design challenge is formulated as an information recovery problem, analogous to the completion of a sentence given its first few words using language models (17) or completion of corrupted images using inpainting (18). A wide variety of protein structure prediction and design challenges can be similarly formulated as missing information recovery problems (Fig. 1D). Although protein inpainting has been explored before (19, 20), here we approach it using the power of a pre-trained structure-prediction network.

We began from a RosettaFold model trained for structure prediction (16) and carried out further training on fixed-backbone sequence design in addition to the standard fixed-sequence structure prediction task (Fig. S3; Materials and Methods). This model, denoted RF_implicit,_ was able to recover small, contiguous regions missing both sequence *and* structure (Fig. S3). Encouraged by this result, we trained a model explicitly on inpainting segments with missing sequence and structure given the surrounding protein context, in addition to sequence design and structure prediction tasks (Fig. S4A; Materials and Methods; Algorithm S1). The resulting model was able to inpaint missing regions with high fidelity (Fig. 1E, S4) and performed well at sequence design (32% native sequence recovery during training, Fig. S4C) and structure prediction (Fig. S4C). We call this network RF_joint_ and use it to generate all inpainted designs below except otherwise noted.

To evaluate *in silico* the quality of designs generated by our methods, we use the AlphaFold (AF) protein structure prediction network (21) which has high accuracy on *de novo* designed proteins (22) (Fig. S7A). RF and AF have different architectures and were trained independently, and hence AF predictions can be regarded as a partially orthogonal *in silico* test of whether RF-designed sequences fold into the intended structures, analogous to traditional *ab initio* folding (13, 24). We used AF to compare the ability of hallucination and inpainting to rebuild missing protein regions (Fig. 1F–G, S5). Inpainting yielded solutions with more accurately predicted fixed regions (“AF-RMSD”; Fig. 1G, S5B) and structures overall more confidently predicted from their amino acid sequences (“AF pLDDT”, Fig. 1F, S5A), and required only 1–10 seconds per design on an NVIDIA RTX2080 GPU (hallucination requires 5–20 minutes per design). However, hallucination gave better results when the missing region was large (Fig. S5) and generated greater structural diversity (Fig. S8, see below).

**Figure 2.**
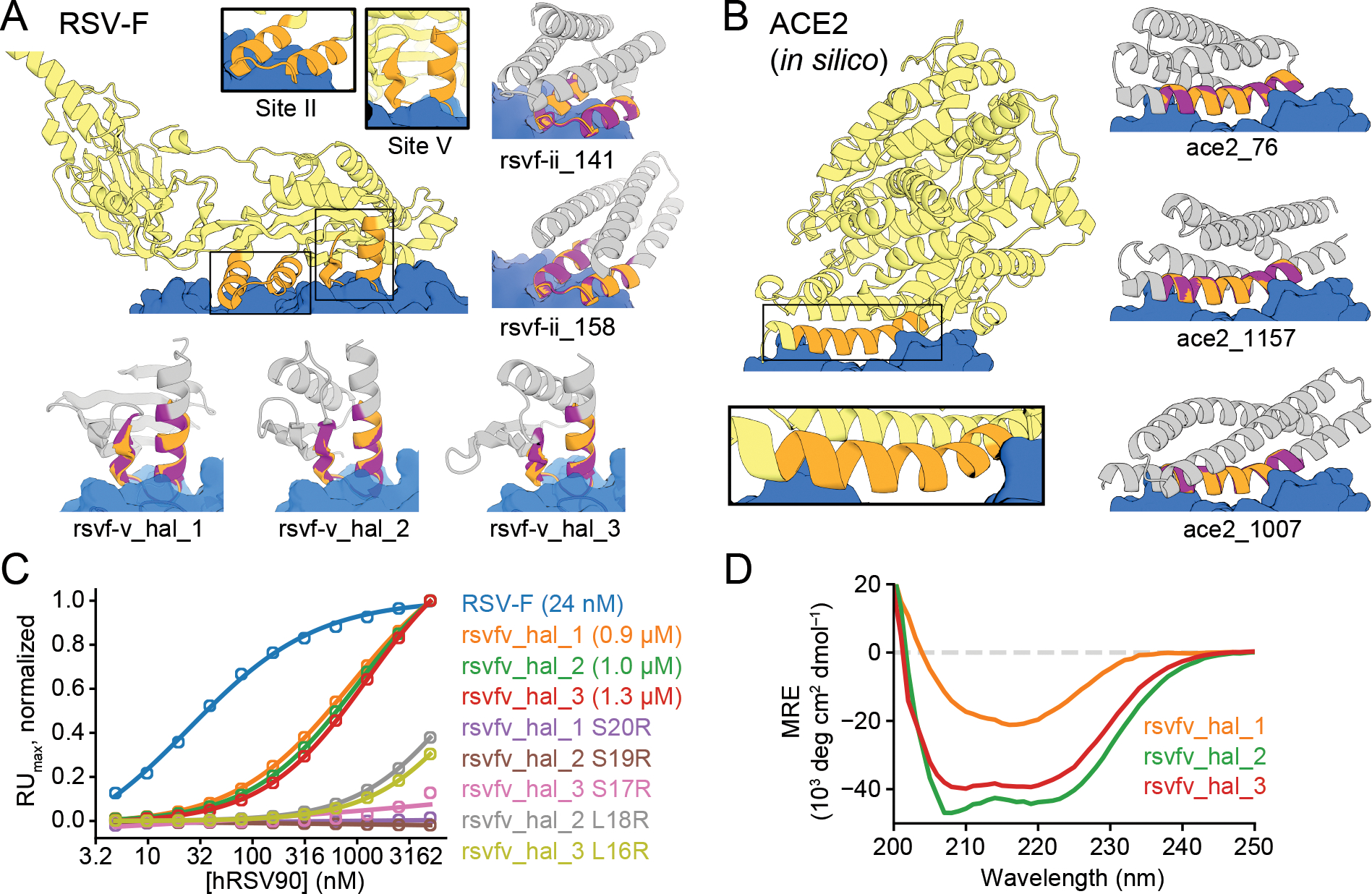
Design of epitope scaffolds and receptor traps. (A) Design of proteins scaffolding immunogenic epitopes on RSV protein F (site II: PDB 3IXT chain P residues 254–277; site V: 5TPN chain A residues 163–181). Comparisons of the RF hallucinated models to AF2 structure predictions from the design sequence are in Fig. S9; here because of space constraints we show only the AF2 model; the two are very close in all cases. Here and in the following figures, we assess the extent of success in designing sequences which fold to structures harboring the desired motif through two metrics computed on the AF2 predictions: prediction confidence (AF pLDDT), and the accuracy of recapitulation of the original scaffolded motif (motif RMSD AF versus native). For RSV-F designs, these metrics are rsvf_ii_141 (85.0, 0.53 Å), rsvf_ii_158 (82.9, 0.51 Å), rsvf_ii_171 (88.4, 0.69 Å); rsvfv_hal_1 (82, 0.7 Å); rsvfv_hal_2 (88, 0.64 Å); rsvfv_hal_3 (86, 0.65 Å). (B) Design of COVID-19 receptor trap based on ACE2 interface helix (6VW1 chain A residues 24–42). Design metrics: ace2_76 (89.1, 0.55 Å); ace2_1157 (80.4, 0.47 Å); ace2_1007 (83.3, 0.57 Å). Colors: native protein scaffold (light yellow); native functional motif (orange); hallucinated scaffold (gray); hallucinated motif (purple); binding partner (blue). See Table S2 for additional metrics on each design. (C) Normalized maximum SPR signal (response units) of purified RSV-F epitope scaffolds and point mutants at various concentrations of hRSV90 antibody, with sigmoid fits. RSV-F refers to purified trimeric native F protein. K_D_ values for each design are shown in legend. (D) Mean residue ellipticity (MRE) versus wavelength, from CD spectroscopy, for the 3 RSV-F site V hallucinations with binding activity.

**Figure 3.**
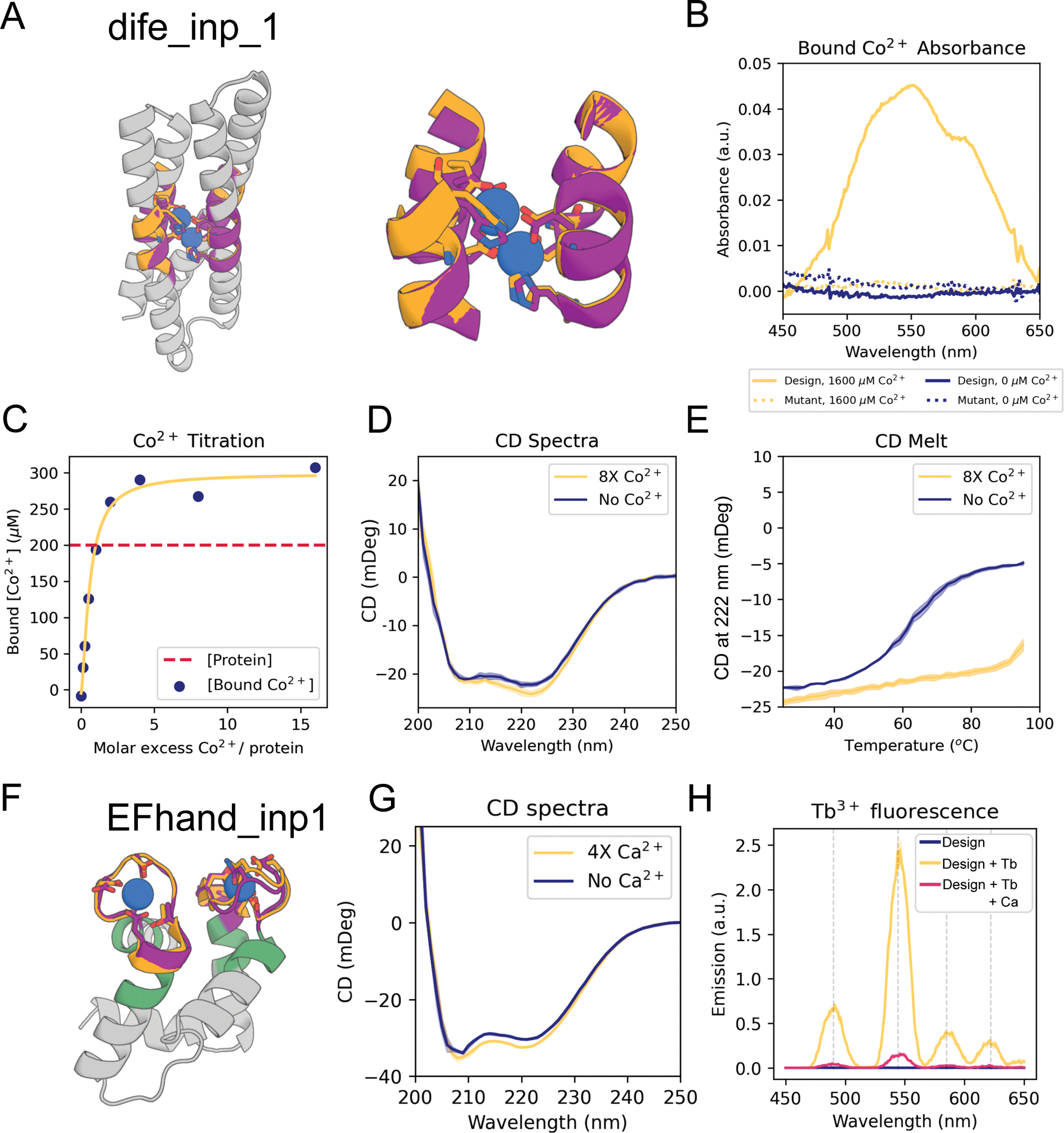
Design of metal binding (A) Di-iron binding site from E. coli cytochrome b1 (1BCF chain A residues 18–25, 27–54, 94–97, 123–130). Colors: native protein scaffold (light yellow); native functional motif (orange); hallucinated scaffold (gray); hallucinated motif (purple); bound metal (blue). Active site residues shown in boxes for di-iron and EF-hand respectively. (B) Absorbance spectra showing of dife_inp_1 (or mutant) in the presence (or not) of an 8-fold molar excess of Co^2+^. Note the peaks at 520 nm, 555 nm and 600 nm, consistent with Co^2+^ binding to the desired scaffolded motif (33). The mutant design was the same sequence but with the 6 coordinating residues (sidechains shown in (A)) mutated to alanine [E16A, E55A, H58A, E89A, H92A, E115A]). Protein concentration was 200 μM. (C) Titration analysis of Co^2+^ against the design (protein concentration = 200 μM). Quantification of the absorbance at 550 nm, using a predicted extinction coefficient of 155 for Co^2+^ binding the motif (33), is consistent with both binding sites being recapitulated in the dife_inp_1 design. (D) CD spectra of design in the presence and absence of Co^2+^. Both spectra are consistent with the predicted helical structure. (E) CD melt curve in the presence and absence of Co^2+^. Note that the coordination of Co^2+^ in the protein core significantly stabilizes dife_inp_1 (protein concentration in CD experiments = 6.7 μM, Co^2+^ concentration = 53.3 μM). (F) AF2 prediction of inpainted design EFhand_inp_1 scaffolding the double EF-hand motif with input motif residues in purple, input non-motif residues in green, and overlaid with the native motif from 1PRW (orange). (G) Tryptophan-enhanced terbium fluorescence spectra of EFhand_inp_1 matches known spectra (57) and suggests the design can bind terbium. (H) CD spectra of EFhand_inp_1 incubated with (4X protein concentration) and without CaCl_2_ suggest stabilization of the protein upon binding calcium. Design metrics (AF pLDDT, motif RMSD AF versus native): dife_inp_1 (92 /0.65 Å), EFhand_inp1 (84, 0.7 Å).

**Figure 4.**
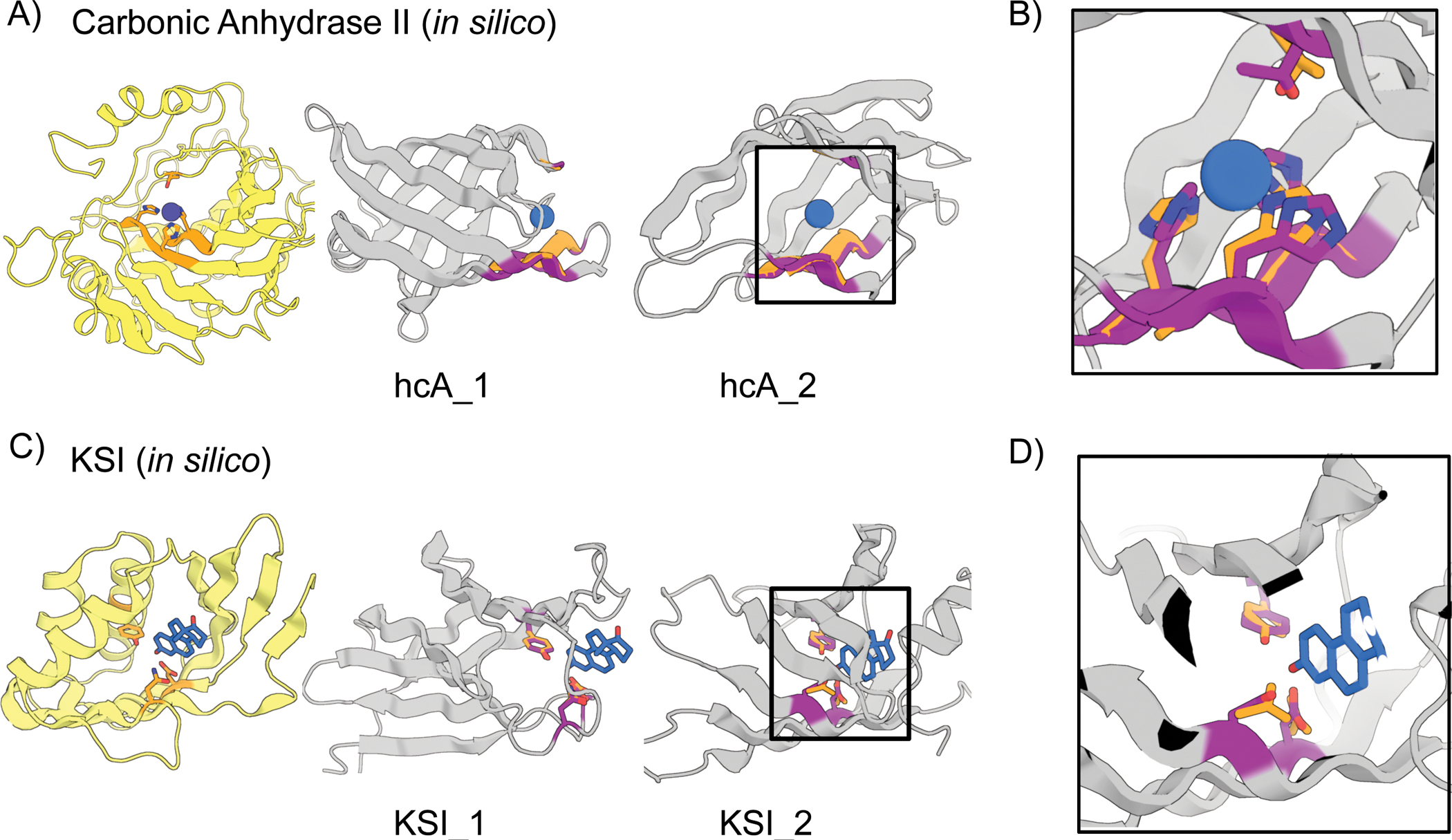
*In silico* design of enzyme active sites. (A-B) Hallucinations using backbone description of site using RF. (C-D) Hallucination using sidechain description of site using AF2 augmented with trRosetta (Materials and Methods). (A) Carbonic anhydrase II active site (5YUI chain A residues 62–65, 93–97, 118–120). (B) Δ^5^-3-ketosteroid Isomerase active site (1QJG chain A residues 14, 38, 99). Colors: native protein scaffold (light yellow); native functional motif (orange); hallucinated scaffold (gray); hallucinated motif (purple); bound metal (blue). Active site residues shown for boxed designs in panel B and for carbonic anhydrase II, and Δ^5^-3-Ketosteroid Isomerase respectively. Design metrics (AF pLDDT, motif RMSD AF versus native): hcA_1 (73, 1.04 Å), hcA_2 (71, 0.62 Å), KSI_1 (84, 0.30 Å Cb), KSI_2 (72, 0.53 Å Cb)

**Figure 5.**
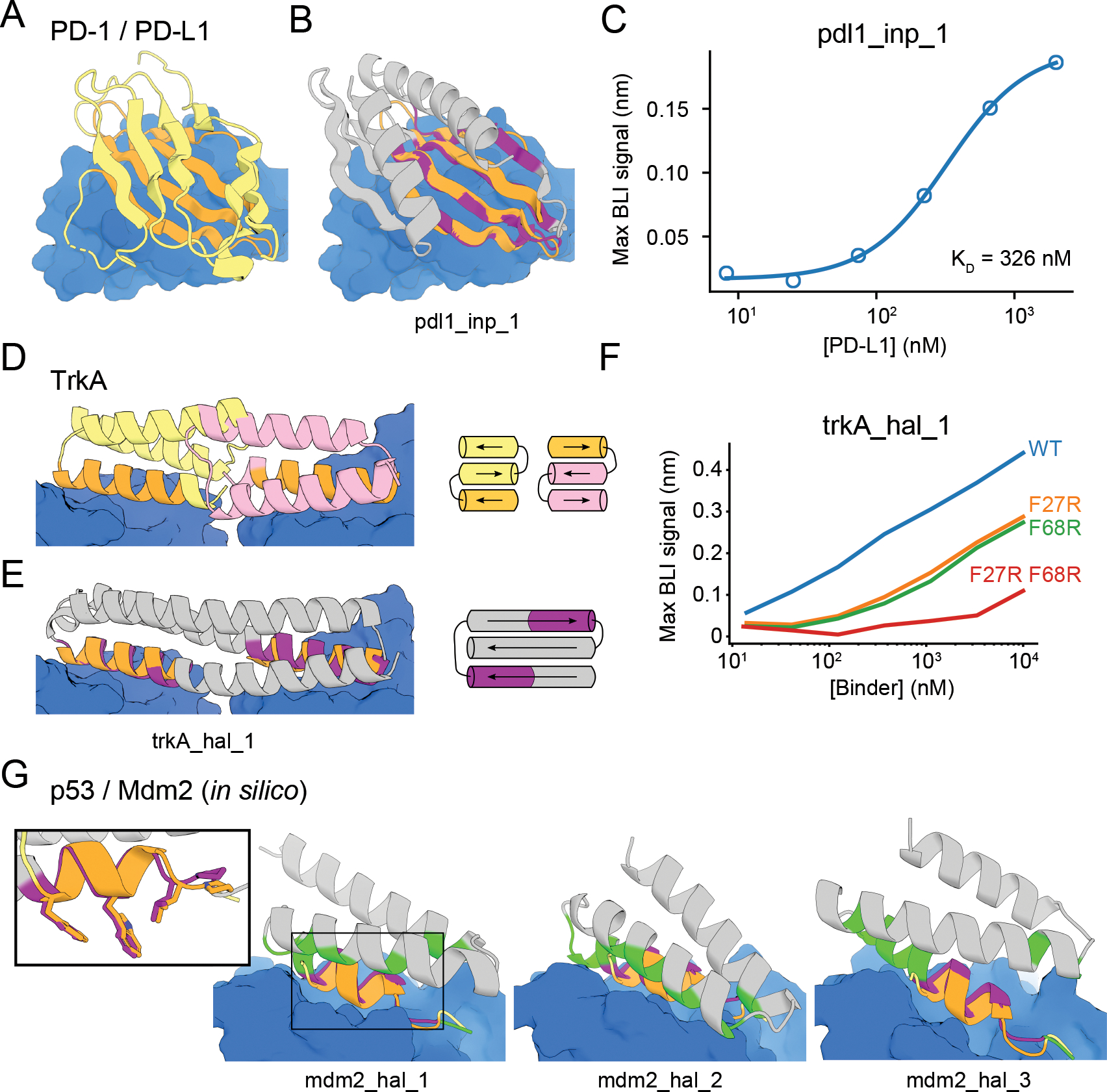
Design of protein-binding proteins. Designs containing target-binding interfaces built around native-complex-derived binding motifs. Targets are in blue, native scaffolds in yellow or pink, native motifs in orange, designed scaffolds in gray and designed motifs in purple. (A) Crystal structure of high-affinity consensus (HAC) PD-1 in complex with PD-L1. (B) Inpainted PD-L1 binder superimposed on PD-1 interface motif. (C) Max BLI binding signal versus PD-L1 concentration. (D) Crystal structure of previously designed TrkA minibinder in complex with TrkA, superimposed on TrkA receptor dimer. (E) Hallucinated bivalent TrkA binder. Protein topologies of (D-E) are shown to the right. (F) Max BLI binding signal versus TrkA concentration, showing that both binding sites bind TrkA. (G) Hallucinated Mdm2 binder designs superimposed on native p53 helix in complex with Mdm2 (see also Fig. S17D–E). New binding interactions (hallucinated residues within 5 Å of the target) are in green. Inset: Overlay of mdm2_hal_1 and native p53 helix showing key sidechains for binding.

In the following sections, we highlight the power of the constrained hallucination and inpainting methods by designing proteins containing a wide range of functional motifs (Fig. 2–5, Table S1). For almost all problems, we obtained designs that are closely recapitulated by AF with overall and motif (functional site) RMSD typically <2 Å and <1 Å respectively, with high model confidence (pLDDT > 80; Table S2); such recapitulation suggests the designed sequences encode the designed structures (although it should be noted that AF has limited ability to predict protein stability (25) or mutational effects (26, 27)). More critically, we assessed the activities of the designs experimentally (with the exception of those labeled “in silico” in Fig. 2–5).

## Designing immunogen candidates and receptor traps

The goal of immunogen design is to scaffold a native epitope recognized by a neutralizing antibody as accurately as possible, in order to elicit antibodies binding the native protein upon immunization. Additional interactions with the antibody are undesirable because the goal is to elicit antibodies recognizing only the original antigen, and hence for hallucination we add a repulsive loss term to penalize interactions with the antibody beyond those present in the scaffolded epitope (Fig. S2; Supplementary Text). As a test case, we focused on respiratory syncytial virus F protein (RSV-F), which has several antigenic epitopes for which structures with neutralizing antibodies have been determined (7, 9, 10). We scaffolded RSV-F site II, a 24-residue helix-loop-helix motif that had previously been grafted successfully onto a 3-helix bundle (7), as well as RSV-F site V, a 19-residue helix-loop-strand motif that has not yet been scaffolded successfully (28). We were able to hallucinate designs recapitulating both epitopes to sub-angstrom backbone RMSD in a variety of folds (Fig. 2A, Fig. S9; structures and sequences for all designs below are in Data S1–2 and differ considerably from native proteins (Table S2); RF and AF models are in Fig. S9, S11, S17; only the AF model is shown in the main figures). Inpainting also generated scaffolds for RSV-F site V, with comparable quality but less diversity than the hallucinations (Fig. S8).

We expressed 37 hallucinated RSV-F site V scaffolds with high AF pLDDT and low motif AF-RMSD in E. coli and found that three bound the neutralizing antibody hRSV90 (28) with Kd’s of 0.9–1.3 uM (Fig. 2C, S11; Methods; Supplementary Text). The Kd for the RSVF trimer is lower (23nM), but the interface is larger encompassing both sites II and V (28). Mutation of either of two key epitope residues reduced or abolished binding of the designs, suggesting that they bind the target through the scaffolded motif (Fig. 2C, S11A), and circular dichroism spectra were consistent with the designed scaffold structures for designs (Fig. 2D) and their point mutants (Fig. S11C). Four of the inpainted designs bound hRSV90 by yeast display, but were poorly expressed in E. coli (Fig. S11C–E). Overall, the designs provide a diverse set of promising starting points for further RSV-F epitope-based vaccine development.

We next applied hallucination to the *in silico* design of receptor traps which neutralize viruses by mimicking their natural binding targets and thus are inherently robust against mutational escape. We again augmented the loss function with a penalty on interactions beyond those in the native receptor to avoid opportunities for viral escape. As a test case, we scaffolded the helix of human angiotensin-converting enzyme 2 (hACE2) interacting with the receptor-binding domain (RBD) of severe acute respiratory syndrome coronavirus 2 (SARS-CoV-2) spike protein (29). The hallucinated hACE2 mimetics have a diverse set of helical topologies, and AF2 structure predictions recapitulate the binding interface with sub-Å accuracy (Fig. 2B, S9C).

## Designing metal-coordinating proteins

Di-iron sites are important in biological systems for iron storage (30) and can mediate catalysis (31, 32). We were able to recapitulate the di-iron site from *E. coli* bacterioferritin, composed of four parallel helical segments, to sub-angstrom AF-RMSD using both inpainting (Fig. 3A–E, S13) and hallucination (Fig. S12; the latter were not tested due to buried polar residues; Supplementary Text). The designs had diverse helix connectivities and low structural similarity to the parent (Fig. S13B, S12; TM-score 0.55–0.71 to 1BCF_A). We chose 96 inpainted designs to test experimentally, and found that 76 had soluble expression, at least 8 (Supplementary Text) had a spectroscopic shift indicative of Co^2+^-binding (a proxy for iron binding) (33, 34), and 3 (dife_inp_1–3, Fig. 3B, S13E) had CD spectra consistent with the designed fold (Fig. 3D, S13F) and were stabilized by metal binding (Fig. 3E, S13G). Mutation of the metal binding residues abolished binding (Fig. 3B, S13E), and titration analysis of dife_inp_1 suggested that both metal binding sites were successfully scaffolded (Fig. 3C).

We next scaffolded the calcium-binding EF-hand motif (35), a 12-residue loop flanked by helices. Both constrained hallucination and inpainting readily generated scaffolds recapitulating either 1 or 2 EF-hand motifs to within 1.0 Å AF-RMSD of the native motif (Fig. 3F, Fig S14A,B, table S2). We chose 20 hallucinations and 55 inpaints to display on yeast and screen for calcium binding using tryptophan-enhanced terbium fluorescence (36). 6 hallucinations and 4 inpaintings had fluorescence consistent with ion binding (Fig. S14A, Materials and Methods; one of these proteins (*EFhand_inp_2*) was designed using RF_implicit_ (Supplementary Text)). The top hit from yeast, the inpainted *EFhand_inp_1*, was purified from E. coli as a monomer (Fig. S14C), had the expected CD spectrum (Fig. 3G) and a clear terbium binding signal (Fig. 3H) which was eliminated by CaCl_2_ competition (Fig. 3H).

## *In silico* design of enzyme active sites

We next sought to scaffold the active site of carbonic anhydrase II, which catalyzes the interconversion of carbon dioxide and bicarbonate and has recently been of interest for carbon sequestration (32–34). The active site consists of 3 Zn^2+^-coordinating histidines on two strands and a threonine on a loop which orients the CO_2_ (Table S1). Despite the complexity of the irregular, discontinuous, 3-segment site, hallucination was able to generate designs with sub-angstrom motif AF-RMSDs with correct His placement for Zn^2+^ coordination (Fig. 4A, S9D); these are less than 100 residues, significantly smaller than the 261 residue native protein.

We next scaffolded the catalytic sidechains of Δ^5^-3-ketosteroid isomerase (Table S1) involved in steroid hormone biosynthesis (37). We attempted to use gradient descent by backpropagation through AF (Materials and Methods; a sidechain-predicting version of RF was not available at the time) but found it difficult to obtain accurate side-chain placement; the landscape may be too rugged with the high resolution sidechain-based loss (Supplementary Text). Better results were obtained with a two-stage approach using first both AF and trRosetta (to smoothen the loss landscape) and a description of the active site at the backbone level, followed by a second all-atom AF-only stage once the overall backbone was roughly in place. This yielded multiple plausible solutions with nearly exact matches to the catalytic sidechain geometry (Fig. 4C–D, S9E). *In silico* validation with a held-out AF model (Materials and Methods) recapitulated the designed active sites. The use of stage-specific loss functions illustrates the ready customizability of the hallucination approach to specific design challenges without network retraining.

## Designing protein-binding proteins

To design binders to the cancer checkpoint protein PD-L1, we scaffolded 2 discontiguous segments of the interfacial beta-sheet from a high-affinity mutant of PD-1 (Fig 5A; Methods) (15). Inpainting yielded designs with not only good AF predictions of the binder monomer (AF pLDDT > 80, motif AF-RMSD < 1.4 Å) but also of the complex between the binder and PD-L1, with an inter-chain predicted alignment error (inter-PAE) of <10 Å (Materials and Methods). Unlike our initial efforts with trRosetta hallucination (Fig. S1, Supplementary Text), it was not necessary to redesign the inpainted sequences using Rosetta. Of 31 designs selected for experimental testing, one design, *pdl1_inp_1*, bound PD-L1 with a *K*_D_ of 326 nM (Fig. 5B–C), worse than HAC PD-1 (*K*_D_ = 110 pM) (38) but better than WT PD-1 (*K*_D_ = 3.9 μM) (38). *pdl1_inp_1* expressed as a monomer (Fig. S15E), was thermostable, and had a CD spectrum consistent with that of a mixed alpha-beta fold (Fig. S15F). Unlike native PD-1, which has a immunoglobulin family beta-sandwich fold, *pdl1_inp_1* has 2 helices buttressing the interfacial beta sheet, as well as an additional 5th inpainted strand extending the interface (Fig. S15 A,B). The closest PDB hit had a TM-score of 0.61 and the closest BLAST NR hit had a sequence identity of 25.4%.

We next used inpainting to design ligands engaging multiple receptor binding sites. The nerve growth factor receptor TrkA dimerizes upon ligand binding (39), and starting from the TrkA-NGF crystal structure we positioned helical segments derived from two copies of a previously designed TrkA binding protein (4) and used hallucination followed by inpainting (Materials and Methods) to scaffold them on a single chain (Fig. 5D–E). A design predicted to be well-structured (AF pLDDT > 80) and interact with TrkA (inter-PAE < 10 Å) was expressed, purified and bound TrkA as assessed by biolayer interferometry (BLI) (Fig. 5F). A double mutant that knocked out both designed binding sites abolished TrkA binding, while single mutants knocking out either one of the binding sites maintained partial binding (Fig. 5F; Fig. S16), suggesting that the protein binds two molecules of TrkA as designed.

RosettaFold is able to predict the structures of protein complexes (40), and we hypothesized that it could generate additional binding interactions between hallucinated or inpainted binder and a target beyond the scaffolded motif. We used a “two-chain” hallucination protocol (Fig. S17, Methods) to design binders to the Mdm2 oncogene by scaffolding the native N-terminal helix of the tumor suppressor protein p53 and obtained diverse designs with AF inter-PAE < 7 Å, target-aligned binder RMSD < 5 Å, binder pLDDT > 85, and SAP score < 35 (Fig. S17D–E); 3 examples are shown in Fig. 5G.

The above approaches to protein-binder design require starting from a previously known binding motif, but hallucination should in principle be able to generate *de novo* interfaces as well. To test this, we used two-chain hallucination to optimize 12-residue peptides for binding to 12 targets starting from random sequences, minimizing an inter-chain entropy loss (Fig. S17H). Most of the hallucinated peptides bound at native protein interaction sites (Fig S18A); the remainder bound in hydrophobic grooves resembling protein binding sites (Fig. S18B). We used the same procedure to generate 55–80-residue binders against TrkA and PDL-1 without starting motif information, and obtained designs predicted by AF to complex with the target, at the native ligand binding site, with a target-aligned binder RMSD < 5 Å and an inter-PAE < 10 Å (Fig. S17F,G).

Unlike classical protein design pipelines, which treat backbone generation and sequence design as two separate problems, our methods simultaneously generate both sequence and structure, taking advantage of the ability of RosettaFold to reason over and jointly optimize both data types. This results in excellent performance in both generating protein backbones with a geometry capable of hosting a desired site and sequences which strongly encode these backbones. Our hallucinated and inpainted backbones accommodate all of the tested functional sites much more accurately than any naturally occurring protein in the PDB or AF predictions database (Fig. S20; Table S3; Supplementary Text) (41), and our designed structures are predicted more confidently from their (single) sequences than most native proteins with known crystal structures, and on par with structurally validated *de novo* designed proteins (Fig. S7A–B). The hallucination and inpainting approaches are complementary: hallucination can generate diverse scaffolds for minimalist functional sites but is computationally expensive because it requires a forward and backward pass through the neural network to calculate gradients for each optimization step (Methods), while inpainting usually requires larger input motifs but is much less compute intensive, and outperforms the hallucination method when more starting information is provided. This difference in performance can be understood by considering the manifold in sequence-structure space corresponding to folded proteins. The inpainting approach can be viewed as projecting an incomplete input sequence-structure pair onto the subset of the manifold of folded proteins (as represented by RosettaFold) containing the functional site--if insufficient starting information is provided, this projection is not well determined, but with sufficient information, it produces protein-like solutions, updating sequence and structure information simultaneously. The loss function used in the hallucination approach is constructed with the goal that minima lie in the protein manifold, but there will likely not be a perfect correspondence, and hence stochastic optimization of the loss function in sequence space may not produce solutions that are as protein-like as those from the inpainting approach.

## Conclusion

The approaches for scaffolding functional sites presented here require no inputs other than the structure and sequence of the desired functional site, and unlike previous methods, do not require specifying the secondary structure or topology of the scaffold and can simultaneously generate both sequence and structure. Despite a recent surge of interest in using machine learning to design protein sequences (42–49), the design of protein structure is relatively underexplored, likely due to the difficulty of efficiently representing and learning structure (50). Generative adversarial networks (GANs) and variational autoencoders (VAEs) have been used to generate protein backbones for specific fold families (51–53), whereas our approach leverages the training of RosettaFold on the entire PDB to generate an almost unlimited diversity of new structures and enable the scaffolding of any desired constellation of functional residues. Our “activation maximization” hallucination approach extends related work in this area (54–56) by leveraging its key strength, the ability to use arbitrary loss functions tailored to specific problems and design any length sequence without retraining. The ability of our inpainting approach to expand from a given functional site to generate a coherent sequence-structure pair should find wide application in protein design because of its speed and generality. The two approaches individually, and the combination of the two, should increase in power as more-accurate protein structure, interface, and small molecule binding prediction networks are developed.

## Supplementary Material

Supplementary Materials

Data S1

Data S2

## Data Availability

Code and neural network weights are available at https://github.com/RosettaCommons/RFDesign and archived at Zenodo (doi: 10.5281/zenodo.6673001). Plasmids of designed proteins are available upon request.
