# Supplementary material for: Scaffolding protein functional sites using deep learning
Source: Science. Author manuscript; Available in PMC 2022 Oct 31. (PMC9621694; doi:10.1126/science.abn2100)
Supplement: Supplementary Materials [file NIHMS1830590-supplement-Supplementary_Materials.docx]

# **Supplementary materials:** Scaffolding protein functional sites using deep learning

Jue Wang^a,b,†^, Sidney Lisanza^a,b,c,†^, David Juergens^a,b,g,†^, Doug Tischer^a,b,†^, Joseph L. Watson^a,b,†^, Karla M. Castro^i^, Robert Ragotte^a,b^, Amijai Saragovi^a,b^, Lukas F. Milles^a,b^, Minkyung Baek^a,b^, Ivan Anishchenko^a,b^, Wei Yang^a,b^, Derrick R. Hicks^a,b^, Marc Expòsit^a,b,g^, Thomas Schlichthaerle^a,b^, Jung-Ho Chun^a,b,c^, Justas Dauparas^a,b^, Nathaniel Bennett^a,b,g^, Basile I. M. Wicky^a,b^, Andrew Muenks^a,b^, Frank DiMaio^a,b^, Bruno Correia^i^, Sergey Ovchinnikov^d,e,*^, David Baker^a,b,f,*^

^a^ Department of Biochemistry, University of Washington, Seattle, WA 98105, USA

^b^ Institute for Protein Design, University of Washington, Seattle, WA 98105, USA

^c^ Graduate program in Biological Physics, Structure and Design, University of Washington, Seattle, WA 98105, USA

^d^ FAS Division of Science, Harvard University, Cambridge, MA 02138, USA

^e^ John Harvard Distinguished Science Fellowship Program, Harvard University, Cambridge, MA 02138, USA

^f^ Howard Hughes Medical Institute, University of Washington, Seattle, WA 98105, USA

^g^ Molecular Engineering Graduate Program, University of Washington, Seattle, WA 98105, USA

^H^ Molecular and Cellular Biology Graduate Program, University of Washington, Seattle, 98195, Washington, USA

^i^Institute of Bioengineering, École Polytechnique Fédérale de Lausanne, Lausanne CH-1015, Switzerland

^†^These authors contributed equally to this work.

* To whom correspondence should be addressed. Email: [dabaker@uw.edu](mailto:dabaker@uw.edu), [so@fas.harvard.edu](mailto:so@fas.harvard.edu)

####

This file contains:

- Materials and Methods
- Supplementary Text
- Figs. S1 to S21
- Tables S1 to S5
- Algorithm S1
- References 59-86
- Data S1 and S2

##

##

## Materials and Methods

### Sequence representation

For structure prediction, the input to trRosetta and RosettaFold is a tensor $X\in\mathbb{R}^{N\times L\times A}$representing a one-hot-encoded multiple sequence alignment (MSA), where *L* is the sequence length, *N* is the number of aligned sequences, and $A=21$ is the alphabet size (20 amino acids plus gap character, although gaps are never used during design). For design with RosettaFold, which was used for most of the designs in this paper, we optimized a single sequence ($N=1$) and applied a 20% dropout, which is implemented at a variety of layers within the network. The first set of PD-1 mimetics (Fig. S1) were hallucinated with trRosetta and optimized a 1000-sequence MSA ($N=1000$) with 0-20% dropout on input 2D features [(*14*)](https://www.zotero.org/google-docs/?dQrWbb). Designing an MSA improves motif accuracy with trRosetta [(*13*)](https://www.zotero.org/google-docs/?2Qilt3) but is not necessary when using RosettaFold. When residues on the functional motif are known to form desirable interactions with the binding partner or a ligand, we constrained these positions to stay the same (native) amino acid during optimization. Conversely, we also included the ability to avoid certain amino acids at all positions (e.g. cysteine). Both capabilities are implemented as adding or subtracting a large number ($10^{8}$) to the sequence logits at the beginning of optimization.

### Loss function

We optimize a loss function

$$\mathcal{L=}w_{M}\mathcal{L}_{M}+w_{H}{\mathcal{L}_{H}+\mathcal{L}_{aux}}$$

consisting of the motif loss $\mathcal{L}_{M}$, which scores the accuracy of the functional site in the design, and a hallucination loss $\mathcal{L}_{H}$, which scores how strongly the sequence encodes a backbone geometry (Fig. 1B), as well as optional auxiliary losses $\mathcal{L}_{aux}$ for specific tasks (Fig. S2; Supplementary Text). For all the designs in this paper we used $w_{M}=w_{H}=1$.

For a protein of length *L*, the motif loss is defined as a negative cross-entropy between reference (one-hot-encoded) and predicted residue-residue geometric feature distributions $p(y)$:

$$\mathcal{L}_{M}= -\sum_{y\in\left\{ d,⍵,\theta,\varphi,\theta^{T},\varphi^{T} \right\}} \left[ \left( \sum_{i =1}^{L} \sum_{j\neq i}^{L} m_{ij}logp(y_{ij}=y_{ij}^{0}) \right)/\left( \sum_{i=1}^{L} \sum_{j\neq i}^{L} m_{ij} \right) \right]$$

where

$$m_{ij}= \left\{ \begin{aligned} 1, \\ 0, \end{aligned} \right.\left. {}_{\text{otherwise}}^{\left\| C\beta_{i}-C\beta_{j} \right\| \leq20 \text{and} i,j\in\text{motif}} \right\}$$

$y\in\left\{ d,⍵,\theta,\varphi,\theta^{T},\varphi^{T} \right\}$ represents residue-residue distances and orientation angles and $y^{0}$ is the value of the distance or angle in the reference motif. The features $d$ and $⍵$ are symmetric while the angles $\theta,\varphi$ are asymmetric, so $\theta^{T}$ and $\varphi^{T}$ are included to match the double-counting of $d$ and $⍵$ across the diagonal. This cross-entropy is averaged over all residue pairs in the motif, represented as a binary mask *m*. We restrict this loss to residue pairs within 20 Å because RosettaFold and trRosetta do not make quantitative predictions beyond this distance. In some cases we supplemented this cross-entropy motif loss with a backbone coordinate RMSD loss (Supplementary Text).

The hallucination loss is defined as the entropy of renormalized network predictions:

$$\mathcal{L}_{H}= \sum_{y\in\left\{ d,⍵,\theta,\varphi,\theta^{T},\varphi^{T} \right\}} \left[ \left( \sum_{i=1}^{L} \sum_{j\neq i}^{L} (1-m_{ij})H\left( \hat{p}(y_{ij}) \right) \right)/\left( \sum_{i=1}^{L} \sum_{j\neq i}^{L} (1-m_{ij}) \right) \right]$$

where the entropy is defined as

$$H\left( p \right)=\sum_{k} p_{k}\log p_{k}$$

and $\hat{p}(y)=\exp\left( \beta\log p\left( y \right) \right)/\sum\exp\left( \beta\log p(y) \right)$. The last of the *K* distance or orientation bins (>20 Å pairwise distance or “no contact”) is excluded to avoid the trivial minimum-entropy solution of an extended chain where most residues are not in contact. Empirically, we found that performing this renormalization with $\beta=10$, and only using bins up to 5 Å for the pairwise distance distributions $p(d)$, gave more realistic structures. In an earlier version of our method we defined the hallucination loss using a KL divergence rather than entropy, which gave similar results (Fig. S2D; Supplementary Text) [(*14*)](https://www.zotero.org/google-docs/?XU6ONe).

### Optimization methods

In early tests, we used an MCMC method based on our previous work on unconstrained hallucination [(](https://www.zotero.org/google-docs/?dTKRdE)*12*[)](https://www.zotero.org/google-docs/?dTKRdE). Starting from a random sequence, single mutations were proposed and the loss function evaluated. The mutation was either accepted or rejected according to the standard Metropolis criterion. Acceptance temperature was 0.002 and annealed by exponential decay with a 500-step half-life; design quality was not sensitive to these parameters. For proteins around 120 residues long, we found this approach converged in about 30,000 steps and took about 90 minutes on Nvidia GeForce RTX2080 GPUs, which we used for all hallucination runs. Although slow, this approach has the advantage that mutations can include insertions and deletions, which is useful when redesigning loops.

For most design problems, we used a gradient-descent method based on our previous fixed backbone sequence design study [(*13*)](https://www.zotero.org/google-docs/?rmZwUn). Starting with randomly initialized input logits $X\sim N_{N\times L\times A}(0, 0.01)$, we apply a softmax followed by an argmax operation to obtain a one-hot-encoding $X_{oh}$. To backpropagate the gradient of the loss $\boldsymbol{\nabla}\mathcal{L}$ through the discrete one-hot sequence to the continuous logits, we employed a reparameterization trick[(*13*, *59*)](https://www.zotero.org/google-docs/?YVNEWn) where gradients were passed through the one-hot sequence as if it had the softmax values of the logits [(*60*, *61*)](https://www.zotero.org/google-docs/?H4xCFr). For a protein of length *L*, on optimization step *t*, we update the input logits with normalized gradients and a constant learning rate $\alpha$:

$$X^{(t+1)}\leftarrow X^{(t)} -\alpha\sqrt{L} \frac{\boldsymbol{\nabla}\mathcal{L}}{\left\| \boldsymbol{\nabla}\mathcal{L} \right\|}$$

Typically we used $\alpha=0.05$, although results are reasonable for any $0.01<\alpha<0.2$(Fig. S19A). We also tested decaying the learning rate over time, but this did not outperform constant learning rate, as seen previously for fixed backbone hallucination [(*13*)](https://www.zotero.org/google-docs/?zKHaZX). With trRosetta, we found that sampling from the softmax distribution over sequence logits [(*59*)](https://www.zotero.org/google-docs/?fxGVGp) yielded higher DAN-lDDT and lower motif RMSD than simply taking the most probable sequence (argmax), but argmax was better when using RosettaFold.

Gradient-based optimization with trRosetta converged in 200 steps for a 120-residue protein, taking approximately 5 minutes on our GPUs, while RosettaFold took 400 steps or 10 minutes per design. A hybrid procedure of gradient descent followed by MCMC yielded improved designs but required much more GPU time, while MCMC-only or MCMC followed by gradient descent yielded inferior results (Fig. S19B-C). In practice, we found that the most efficient use of GPU time was to first generate designs using gradient descent to sample a diverse structural space (and explore hyperparameters such as motif placements and sequence length), then use the best resulting designs to “seed” many short MCMC trajectories (300-1000 steps) to obtain further-refined and diversified final designs.

### Motif placement

At the beginning of optimization, each discontinuous segment of the motif is mapped to a random block of residue positions on the designed sequence. The motif loss is applied to these “constrained” regions, while the hallucination loss is applied to the remaining residue positions. The positions corresponding to the motif stay fixed during optimization. For each new problem, we start by specifying a range for the total protein length *L* and generate many designs with randomly sampled *L* from the range and randomly placed motif segments. We then identify the values of *L* and inter-segment gap lengths that yielded the best designs and run followup design trajectories with these parameters in order to deeply sample productive regions of the search space. In early testing, we developed algorithms which adaptively place motifs during optimization either by minimizing motif loss over all possible placements or performing a greedy search (Supplementary Text). While potentially useful for certain problems, these were not consistently better than the simpler fixed-placement strategy (Fig. S19D-E).

### Scaffolding enzyme active sites using AlphaFold

To design de novo scaffolds for the active site of ∆^5^-3-ketosteroid isomerase (KSI) [(*36*)](https://www.zotero.org/google-docs/?iekfzn), we used AF in a two-stage method, the first stage focusing on backbone generation and the second on sidechain geometry optimization. In stage 1, we perform 200 steps of gradient descent to optimize a real-valued tensor $X\in\mathbb{R}^{1\times L\times A}$ representing sequence logits. The argmax of the softmax of the logits is used as input to AF and trRosetta. To allow backpropagation through the argmax function, we use the gradient straight-through trick as described previously [(13)](https://www.zotero.org/google-docs/?aK9rmt). Gradients are obtained from both AF and trRosetta, weighted equally, and used to update the logits X. Losses used for AF are the predicted LDDT and aligned error (for hallucination) and Cb distogram CCE (for motif recapitulation, defined similarly as the CCE used with RosettaFold above), sidechain FAPE [(*21*)](https://www.zotero.org/google-docs/?wco8xS) and RMSD (root-mean-squared-deviation); losses for trRosetta are KL divergence (Supplementary Text) and CCE, but excluding the theta dihedral. Stage 1 is run using the ADAM optimizer [(*62*)](https://www.zotero.org/google-docs/?9CVvP4) with a learning rate of 5e-3. The gradients are normalized by the norm at each iteration. We found that if we do not use trRosetta as part of the loss, it is very unstable and the motif RMSD rarely goes below 2 Å (see further discussion in Supplementary Text). In stage 2, the sequence from stage 1 is subjected to 400 steps of semi-greedy optimization using AF: at each step a random position is mutated, if the loss decreases, the mutation is accepted, if not, up to 20 independent random mutations are attempted. If none of the 20 mutations decreased loss, the mutation with best loss is accepted. For the first stage, 400 independent designs were generated. Each design had 3 random indices between 0 and 99 selected to define the positions of the active site. The top 4 designs were selected for stage 2. The loss for stage 2 is the weighted sum of predicted LDDT and aligned error, and sidechain FAPE and RMSD. The confidence loss was scaled by 0.01 and sidechain loss by 1.0. To attempt to avoid false local optima in a particular set of AF weights, during stage 2 we evaluated the loss using a randomly chosen one of 4 AF models (model_1_ptm, model_2_ptm, model_3_ptm, and model_5_ptm) (sets of weights) on each step. This is similar to averaging the 4 models [(*54*, *55*)](https://www.zotero.org/google-docs/?Qczg9x) but is more compute efficient. We withhold model_4_ptm for validation -- the designs shown in the figures come from this model.

### Protein binder “two-chain” hallucination

#### Expanding interfaces around native binding motif

To design Mdm2 binders, we first used standard hallucination to scaffold the p53 helix, with the repulsive loss on. These designs are roughly shape-complementary to Mdm2 but do not make biochemical interactions. We then refined a small number of high-scoring designs by 100-1000 steps of MCMC with RosettaFold predicting the entire binder/target complex but only optimizing the binder sequence. We predicted complexes by concatenating the binder and target sequences with a 200 amino-acid gap between them in the residue index input to RosettaFold [(*16*)](https://www.zotero.org/google-docs/?UNgpeM). RosettaFold has limited accuracy predicting native protein structures and complexes from single sequences. To ensure that the target is accurately predicted (as this is a prerequisite for accurately hallucinating interactions to it) we input the structure of the target plus the stub as homology templates to RosettaFold (Fig. S17A). As expected, this usually yielded predictions of the target (and target-stub relative position) extremely close to the crystal structure. During 2-chain refinement, we applied the motif loss to preserve the structure of the binder and its relative position to the target and the hallucination loss to the rest of the binder to encourage formation of interactions with the target; no repulsive or attractive losses were used. For this task, gradient descent did not give good results, and MCMC refinement of a previously hallucinated monomer was the most efficient and robust approach.

#### Free hallucination

To generate the 12-residue stubs against various targets, as well as binder designs against TrkA and PD-L1 without using a pre-specified motif, we initialized a completely random sequence of a pre-defined length (12 AAs or 55-80 AAs) and concatenated it to the sequence of the target (Fig. S17A). On each iteration we predicted the structure of the complex using template input for the target, as described above. To promote binder-target contacts, we used an “inter-chain” entropy loss which was computed only on the inter-chain residue pairs and given a weight of 1 to 5 (Supplementary Text); the usual (intra-chain) entropy loss (with weight 1) was also used, to promote hallucination of a well-packed binder monomer. The entropy calculation was modified in some cases to improve handling of the “no-contact” bin (see “Leaky entropy” in Supplementary Text). For the stub design problem (Fig. S18), we ran 600 steps of MCMC (gradient descent was not possible for these targets due to GPU memory limitations); for TrkA and PD-L1 (Fig. S17F-G), we ran 200-400 gradient descent steps followed by 200-300 MCMC steps. Multiple rounds of filtering and design refinement/diversification were performed (Supplementary Text).

### Training RosettaFold to jointly model sequence and structure (RF_joint_)

Standard RosettaFold [(*16*)](https://www.zotero.org/google-docs/?4SxP7s) (RF) has been trained on structure prediction (sequence inputs, structure outputs) using homolog templates (structure input). In the newer versions, we mask a portion of the input MSA and apply a loss to predictions of the masked amino acids (sequence output) to encourage the network to extract more meaning from the MSA [(*21*, *63*)](https://www.zotero.org/google-docs/?stM3ia). RF_joint_ was fine tuned from a pre-trained RosettaFold model (RF-Nov05-2021, see Supplementary Text, “RosettaFold variants” section for details on the architectural details of this model). The training regime for this model, which was initially trained solely on structure prediction, is below:

Training set: 25% of examples came from the PDB (published before February 17th, 2020), which is the same training set used in the original RosettaFold model [(*16*)](https://www.zotero.org/google-docs/?1BkV1H). The other 75% of examples included a distillation set of AlphaFold predicted structures [(*64*)](https://www.zotero.org/google-docs/?Q3iN1c). This distillation set was clustered at 30% sequence identity cutoff, and sequences sharing greater than 30% similarity to any protein in the PDB were excluded. Only proteins greater than 200 residues in length, with mean AlphaFold pLDDT > 85 were included in training, and only residues with per-residue pLDDT > 70 were included from these models. The AdamW Optimizer was used throughout training, with default pytorch parameters. The epoch size was 25600 training examples, with a batch size of 64. The learning rate for the initial round of training (200 epochs) was 0.001, with a linear warm-up for the first 1000 optimization steps. The learning rate was then decayed by a factor of 0.95 after every 10000 optimization steps. A crop size of 256 residues was used, with cropping following the same strategy as described previously [(*16*)](https://www.zotero.org/google-docs/?4KUn1h). The number of MSA seed sequences was 128, and the number of extra MSA sequences was 1024. For the second stage of training (100 epochs), the learning rate was set of 0.0005 (no warm-up), with learning rate decay by a factor of 0.95 every 10000 optimization steps. A larger crop size (350 residues), and more MSA sequences (256 seed sequences, 2048 extra sequences) were used in this second phase of training.

Starting with this pre-trained RosettaFold, we fine-tuned this model for inpainting, for an additional 27 epochs on three tasks (Fig. S4), training only on the PDB training set. For tasks 1 and 2 (fixed backbone sequence design, and inpainting respectively, chosen 33% of the time each) were masked in essentially the same manner. Contiguous regions of 10-35 amino acids comprising at least one full secondary structure element (helix, loop or strand) were masked out (Task 1: only sequence masked; Task 2: sequence and structure masked). The sequence and structure of a further 3-6 ‘flanking’ residues were masked out either side of this contiguous region (Fig. S4A, red). The distograms (but not angle maps or amino acid identity) were provided for the residue immediately N- and C-terminal to the central contiguous masked region (Fig. S4A, asterisks). Noise was also applied to these two positions, by randomly translating them following a normal distribution (μ = 0 Å, σ = 1 Å), such that at inference time, coordinates would be provided to the network as a “guide” rather than as absolute positions. Losses were not applied to the flanking regions either side of these two coordinates. The masking of flanking sequence and structure modestly improved the performance of the network in the benchmarking test, compared to just masking a 10-35 residue window (Fig. S4D). The final task (structure prediction from MSA information) was the original task the pre-trained RosettaFold was trained on, which differs slightly from the original RosettaFold network (*15*). Specifically, in this task, 15% of the MSA (excluding the input sequence) was randomly masked or corrupted (following the strategy used by AlphaFold [(*21*)](https://www.zotero.org/google-docs/?HS63iR), of this 15% of residues, 70% of residues were replaced with a ‘mask’ token, 10% were mutated to a random amino acid, 10% were mutated to another amino acid in the MSA column, and 10% were not replaced). Homologous template structural inputs were unchanged from the original network (*15*). The applied loss function was the same for all three tasks:

The loss function formulation for RF*_joint_* is as follows.

$$\mathcal{L}_{total}={{1.0\mathcal{L}}_{dist}+ 3.0\mathcal{L}}_{aa}+{{1.0\mathcal{L}}_{tors}+5.0\mathcal{L}}_{FAPE}+{0.1\mathcal{L}}_{lddt}$$

Where $\mathcal{L}_{dist}$ is a cross entropy loss over the distogram and anglegram as described in (*15*), predictions $\mathcal{L}_{aa}$ is a cross entropy loss over any masked positions in the input MSA, $\mathcal{L}_{tors}$ is a cross entropy loss on binned backbone dihedral angle predictions, $\mathcal{L}_{FAPE}$ is a backbone level frame aligned point error, as described in [(*21*)](https://www.zotero.org/google-docs/?WS7CJp), with a relu cutoff of 20. $\mathcal{L}_{lddt}$ is the lDDT loss as calculated in (*15*). Note that structure related losses are applied over the entire predicted protein, and the sequence cross entropy loss is only applied at masked (Tasks 1 and 2) and/or corrupted (Task 3) regions. For the fixed-backbone sequence design task (Fig. S4A, Task 1) and for the inpainting task (Fig. S4A, Task 2), no loss was applied on the ‘flanking’ region of protein N- and C-terminal to the central masked region. The learning rate was set to 0.0003 throughout the training of these three tasks, with a batch size of 512. We refer to this fine-tuned RosettaFold inpainting model as RF*_joint_*, and selected training curves from this model are shown in Fig. S4B,C. Details of a different training strategy used to train an earlier version of the inpainting network, which *implicitly* learned to inpaint, are provided in the supplementary methods.

### Joint sequence-structure inpainting with a jointly trained RosettaFold

To apply RF_joint_ to protein design, we input a sequence and structure, masking certain residues in the sequence by replacing them with mask tokens and masking corresponding residues in the structure by setting their template embeddings to zero [(*16*)](https://www.zotero.org/google-docs/?oXQKFm). We then predict the structure and sequence logits for the entire protein. The output structure, including regions that were originally both masked and unmasked, is used as the design model, and the most probable predicted amino acid at each masked position (argmax) is taken to complete the sequence. Note that in the RF-Nov05-2021 version of RosettaFold used to train RF_joint,_ as in AlphaFold, latent representations of the output structure are ‘recycled’ back through the network to refine the final structure. During inpainting, we utilize this ‘recycling’ to refine our inpainted sequence and structure, typically recycling information 5-15 times (similar to the number of times used for structure prediction with RosettaFold, which is typically 10). A single design of 100 amino acids in length, using 10 iterations of inpainting, takes 5.3 seconds on a GeForce RTX 2080 GPU. We refer to this prediction, with recycling, as a ‘forward pass’ through the network.

The iterative inpainting method described above is approximately deterministic. To sample ensembles of outputs with small variations in sequence and structure using RF*_joint_*, we either vary the exact boundaries of masked regions, the length of regions to replace a masked region or by varying specific input coordinates (for example, in Fig. S6C, the coordinates of two Cɑ-coordinates were randomly translated up to a specified distance from their original positions, and the network was tasked with inpainting the masked region given the unmasked positions of the two translated residues). For each of the design cases presented in the paper, the precise strategy used to generate and filter the designs is described in the supplementary methods.

### Motif selection

Because RosettaFold predicts helices and sheets more accurately than loops, we selected functional motifs composed of as much secondary structure as possible. In initial exploratory design runs, our methods performed poorly if the motif to scaffold contained too many loops or depended on networks of tertiary polar contacts (e.g. antibody H3 CDR regions). For antigenic epitopes, viral receptor traps, and enzyme active sites, we chose the functional motifs based on previous structural literature. For binding interfaces, we identified interface residues as those with any atom within 5 Å of the binding partner and scaffolded motifs consisting of 2-4 contiguous blocks manually chosen to contain as many of the interface residues as possible. Table S1 lists the design targets, their PDB accessions, the residue numbers of constrained regions, and references.

### Design filtering & selection

For each experimentally tested design case shown in this paper, we generated between 4000 and 30,000 designs, and filtered these based on the AF pLDDT, motif RMSD of AF predictions to native, (see supplementary text for exact cutoffs). Broadly, these included ‘confident/accurate’ AF pLDDT (> 80), sub-angstrom (< 1 Å) AF-RMSD. Orthogonal filters were determined on a per-problem basis (fully outlined in the supplementary text), but broadly comprised features such as radius of gyration, Rosetta per-residue spatial aggregation propensity (SAP) score [(*65*)](https://www.zotero.org/google-docs/?FKpQ6F), net charge (# Arg + # Lys - # Asp - # Glu) and structural diversity. The cutoffs were typically chosen to give an experimentally tractable final number of designs. In some cases, in preparation of the final set of proteins to be ordered, and after design filtering, we performed a final visual inspection to look qualitatively at aspects such as poor core packing, presence of cavities, buried polar groups, or surface hydrophobics, which typically reduced the set of proteins by around 0-50%.

For designs that were only validated *in silico*, that are represented in the figures, we filtered designs predominantly on AlphaFold pLDDT and AF-RMSD, as well as radius of gyration. The AlphaFold metrics are presented in Table S2.

The “model 4” weights were used for all AF predictions for filtering. The pLDDT was taken as the average of the residue-wise confidence values output by the network. Using AF to filter our designs has the risk of designing “adversarial examples”, or sequence-structure pairs that score well by AF that do not fold or function in reality, due to the presence of artifactual minima in the loss landscape of the structure-prediction model [(*66*, *67*)](https://www.zotero.org/google-docs/?vktxPh). However, because we design using RosettaFold, which is trained independently of AF (although both use the PDB as training data), any final designs must be well-predicted by two partially orthogonal networks, which is expected to provide some (although not total [(*68*)](https://www.zotero.org/google-docs/?nvYsOt)) robustness to adversarial examples. This is supported by our finding that a high fraction of our designs are solubly expressed. Additionally, if we redesign the sequence of our highest-pLDDT designs by Rosetta, pLDDT continues to be high, indicating that the original hallucination had a designable backbone (and isn’t purely an artifact of RF or AF’s loss landscape) (Fig. S7C). Finally, we find that AF pLDDT of our RF-generated designs correlate well with physics-based metrics such as Rosetta energy and *ab initio* folding (Fig. S7D, F; Supplementary Text).

To score protein binder designs, we used a modified AlphaFold prediction script that took as input the design model of the target-binder complex (from RF hallucination or inpainting) and the concatenated binder-target sequence (with a residue number gap to denote different chains). AF was asked to predict the complex structure from single-sequence, given the target protein structure as template information and its structural representation (atom coordinates) of the binder-target complex initialized to the target-binder complex design model. The confidence in AF2’s prediction of the interface was assessed by the inter-chain predicted aligned error (inter-PAE), or the average value of interchain positions in the predicted aligned error matrix. We found that inter-PAE < 10 Å corresponded to predicted complexes that were docked roughly correctly, while predictions with inter-PAE above this threshold usually had binder and target far apart in space. In addition to inter-PAE, we also filtered on: binder pLDDT (average residue-wise confidence over the binder from complex prediction); AF-Rosetta ddG (Rosetta ddG calculated on the AF model after minimizing interface side chains); target-aligned binder RMSD (RMSD of the binder, after aligning AF and RF models on the target).

### Protein purification

All designs tested in E. Coli were cloned, expressed and purified using standard methods. Briefly, Golden Gate assembly with BsaI-HF (New England Biolabs) was used to insert designs into a modified pET29b+ vector containing C-terminal SNAC [(*69*)](https://www.zotero.org/google-docs/?U3sJxP) and 6xHis tags (or, in the case of *EFhand_inp_1*, into a modified pET29b+ vector with a C-terminal TEV cleavage site and a 6xHis tag). Plasmids were transformed into BL21 bacteria. For small-scale expression tests, bacteria were cultured overnight at 37^o^C in 2 ml cultures of lysogeny broth (LB) supplemented with 50 μg/mL of kanamycin. Cells were then grown in 2 ml cultures of Terrific Broth (TB) for one hour, before induction with 1 mM of IPTG for 4 hours. Cells were then lysed with B-PER supplemented with 1 mM PMSF, 0.1 mg/mL Lysozyme, 25 U/ml Benzonase, before lysate clarification by centrifugation. Lysate was incubated with 75 μl Ni-NTA resin, before washing thrice with wash buffer (25 mM Tris, 300 mM NaCl, 20 mM Imidazole, pH 7.8) and elution in 25 mM Tris, 300 mM NaCl, 250 mM Imidazole. Expression was assessed by SDS-PAGE. For larger scale cultures, cultures were grown overnight at 37^o^C in autoinduction medium [(*70*)](https://www.zotero.org/google-docs/?j9E5eB), before sonication-based lysis in wash buffer supplemented with 1mM PMSF, 0.1 mg/mL Lysozyme, 0.01 mg/ml DNase I. After centrifugal lysate clarification, lysates were incubated with an appropriate volume of Ni-NTA resin and subsequently washed thrice with wash buffer. For purification of di-iron binding proteins, the His-tag was cleaved off by cleavage of the SNAC-tag. Briefly, after binding to the Ni-NTA resin, the protein was washed in SNAC cleavage buffer (100 mM CHES, 100 mM Acetone oxime, 100 mM NaCl, 500mM GuHCl, pH 8.6) before addition of 2 mM NiCl_2_. After overnight cleavage, proteins were further purified by size exclusion chromatography on a Superose 75 column in 20 mM Hepes, 100 mM KCl, pH 7.8, and monomeric fractions pooled.

### Spectroscopic analysis of cobalt binding to di-iron binding proteins

Analysis of cobalt binding to inpainted di-iron binders was performed essentially as described previously [(*32*)](https://www.zotero.org/google-docs/?RDcwzY). Proteins (200 μM in 20 mM Hepes, 100 mM KCl, pH 7.8) were incubated overnight with (or not) an 8x molar excess (1600 μM) CoCl_2_. Absorbance spectra were collected in a Jason V-750 spectrophotometer. Mean background absorbance (measured between 700 and 800 nm) were subtracted from all spectra. Successful designs showed absorbance peaks characteristic of cobalt coordinated in a tetra/penta-coordinate state.

### Fluorescence analysis of terbium binding to EF-hand designs

#### Yeast-displayed designs

Transformed yeast were cultured in TRP(-), URA(-) media for two days followed by expression culture. Samples containing ~8.5e7 cells were incubated in TBS (pH 8.0) containing 1mM Ca^2+^ and washed twice with TBS only. Yeast cells were resuspended in TBS containing 50 μM Tb^3+^ For 3 hours and then washed twice in TBS + 1mM Ca^2+^. Washed samples were moved to a black bottom, plate-reader 96 plates for fluorescence spectra measurement. Fluorescence signals were collected using a flash plate reader in time-resolved fluorescence mode (TRF, delay time: 100us , integration time: 1000us, gain: 130).

#### Purified designs

Designs harboring the EF-hand motif , were purified by His-purification as described above. After size exclusion chromatography in 20 mM Hepes, 150 mM KCl, pH 7.8, the His tag was cleaved by TEV-cleavage, with the addition of 40 μM Super-TEV protease, 1 mM DTT and 0.5 mM EDTA (overnight at room temperature). To ensure the EF-hands were not bound to any residual calcium in buffers, after passing through a NiNTA-column after TEV-cleavage, protein were run on a size exclusion column equilibrated in 20 mM Hepes, 150 mM KCl, pH 7.8 buffer, which had been Chelex treated overnight to remove any residual calcium. Proteins were incubated (or not) with terbium (40 μM terbium in 5 μM protein) for 3 hours, before analysis of terbium fluorescence on a NEO2 plate reader. Samples were excited at 250 nm (to excite the tryptophan residue near the EF-hand motif), and fluorescence was measured between 450 and 650 nm, 100-1000 μs after excitation.

### Circular dichroism spectroscopy

All circular dichroism (CD) analyses except those for RSV-F site V immunogens were performed on a JASCO J-1500 CD Spectrophotometer. Di-iron binding proteins were analyzed at 6.7 μM in 20 mM Hepes, 10 mM KCl, pH 7.8, with or without an 8x molar excess of CoCl_2_. Analysis of the EF-hand inpaint was performed at 20 μM in chelex100-treated 20 mM Hepes, 150 mM KF, pH 7.6, in the presence or absence of 200 μM CaCl_2_. Analysis of the PDL-1 binder was performed at 5 μM in 20 mM Hepes, 10 mM KCl, pH 7.8. Thermal melt analyses were performed between 25 ^o^C and 95 ^o^C, measuring CD at 222 nm. All reported measurements were measured within the linear range of the instrument.

For RSV-F designs, CD spectra were measured using a ChirascanTm V100 spectrometer in a 1-mm path-length cuvette. The protein samples were diluted to 30 µM in PBS. Wavelengths between 195 nm and 250 nm were recorded. Thermal melt analyses were performed between 20 °C and 95 °C with an increment of 2 °C/min, measuring CD at 222 nm. All spectra were corrected for buffer absorption.

### Measuring protein binding

#### Yeast surface display

As an initial screen for protein binding, linear DNA were synthesized as “e-blocks” (Integrated DNA Technologies), pooled, and transformed into the yeast strain EBY100 (by electroporation if >100 designs, by the lithium acetate method otherwise) along with a pETCON3 backbone linearized at NdeI and XhoI (for Aga2p and c-Myc fusion) [(*4*, *5*)](https://www.zotero.org/google-docs/?k0a3Ty). The transformed pool was inoculated into CTUG medium (yeast nitrogen base 6.7g/L (difco) + complete amino acids -trp -ura + 2% glucose) and incubated 12-16 hours at 30°C with shaking, then diluted 200uL + 2mL into SGCAA (yeast nitrogen base 6.7g/L + complete amino acids 5g/L (Bacto) + 90mM Na_2_HPO_4_ + 2% galactose + 0.1% glucose) and incubated 12-16 hours to induce binder expression and display. For flow sorting, around 10^7^ cells were harvested, washed 3x in TBSF (50mM Tris-HCl pH8.0, 150mM NaCl, 1% bovine serum albumin), incubated in TBSF with biotinylated binding target for 30 minutes at room temperature, washed 1x in TBSF, incubated for 30 minutes at room temperature in 0.1mg/mL FITC anti-c-Myc (ICL Lab) and 70mg/mL streptavidin R-phycoerythrin (PE) conjugate (Invitrogen), and washed 3x in TBSF. The binding target and FITC/PE were added in the same incubation when labeling with avidity. Cells were sorted on a Sony SH800 flow sorter and 10^3^ - 10^6^ FITC+/PE+ cells were collected. The cells were either cultured in liquid CTUG for another round of sorting, or plated onto CTUG agar and individual colonies Sanger-sequenced to identify the designs. For trRosetta-hallucinated PD-L1 binders and Mdm2 binders, clonal yeast cultures expressing a single design were analyzed in binding assays to confirm the results of sorting as well as to assess the binding affinity of designs. In this case, yeast culture and binding were performed identically as above except that an Attune NxT (Invitrogen) flow cytometer was used to analyze the cells. For all other problems, hits identified by yeast display were followed up by *E. coli* expression and purification.

#### Surface plasmon resonance (SPR) to assess RSV-F site V binding

SPR measurements were performed on a Biacore 8K (GE Healthcare) in 10 mM HEPES pH 7.4, 150 mM NaCl, 3 mM EDTA, 0.005% v/v Surfactant P20 (GE Healthcare). Ligands were immobilized on a CM5 chip (GE Healthcare) via amine coupling. The preRSVF and RSVF-site V immunogens were immobilized at approximately 300-500 response units (RU). The site V specific RSV90 Fab was injected as analyte in two-fold serial dilutions. The flow rate was 30 µl/min for a contact time of 120 s followed by 400 s dissociation time. After each injection, the surface was regenerated using 0.1 M glycine at pH 3.0. *K*_D_ values were obtained by fitting the maximum response versus log10 Fab concentration to a sigmoid function using GraphPad PRISM.

#### Biolayer interferometry (BLI) to assess bivalent TrkA binding

BLI binding experiments were performed on an Octet Red96 (ForteBio), with streptavidin coated tips (Sartorius Item no. 18-5019) and BLI buffer (10 fold dilution of 10x HBS-EP+ buffer [Cytiva Item no. BR100669] supplemented with 0.1% w/v bovine serum albumin). Tips were pre-incubated in BLI buffer for at least 30 minutes before use. To collect binding data, the tips were incubated in BLI buffer for 100 s, loaded with biotinylated TrkA (30 nM in BLI buffer; a kind gift from Chris Garcia’s lab) for 300 s, equilibrated in BLI buffer to obtain a baseline for 150 s , dipped into BLI buffer with the designed proteins for 900 s (association phase) and finally returned to BLI buffer for 900 s (dissociation phase). Reported responses are the change in wavelength between the beginning and end of the association phase.

### Similarity search against the PDB and NR databases

We used TM-align [(*71*)](https://www.zotero.org/google-docs/?mBQIF0) to calculate the TM-score of a design against all chains in a local copy of the March 3, 2021 version of PDB100. TM-scores reported are normalized to the length of the query (designed) protein.

We used Protein-Protein BLAST 2.9.0+ to query our design sequences against a version of the BLAST NCBI non redundant database downloaded in April 2020. For each design, we ran the blastp executable with flags -outfmt 15 -max_hsps 1 -max_target_seqs 1, thus only taking the best high scoring pair (HSP) and reporting its statistics for each design. The sequence identities quoted throughout the manuscript were calculated by taking the number of identities contained in the HSP and dividing by the length of the query (designed) sequence. In some cases, BLAST did not return any HSP’s, in which case the entries from BLAST for these designs in Table S5 were marked “None”/ “NA”.

## Supplementary Text

### RosettaFold variants

The hallucination pipeline uses the following neural networks.

- The published version of trRosetta [(*11*)](https://www.zotero.org/google-docs/?O7WwqL), used for the PD-L1 binder designs in Fig. S1.
- The published 2-track RosettaFold [(*16*)](https://www.zotero.org/google-docs/?1CgRTG), used for hyperparameter tuning results shown in Fig. S19.
- The published 3-track RosettaFold [(*16*)](https://www.zotero.org/google-docs/?KJyR2g), used for some of the designs shown in Fig. S10.
- An unpublished “RF-perceiver” where the MSA Transformer [(*63*)](https://www.zotero.org/google-docs/?o6PidK) track of RosettaFold is replaced with a Perceiver architecture [(*72*)](https://www.zotero.org/google-docs/?bgnZA9) to reduce memory cost for MSAs having too many sequences. Instead of making all sequence-to-all sequence attention for the input MSA, the input MSA is split into two groups, a small seed MSA (up to 128 sequences) and an extra MSA (up to 2048 sequences). It first takes a cross-attention that maps the extra MSA to the seed MSA, then takes a regular self-attention on the seed MSA. During training, backbone-level Frame Aligned Point Error (FAPE) loss [(*21*)](https://www.zotero.org/google-docs/?lzsxlf) was used. For the hallucination task, because input is a single sequence, extra MSA features were initialized to zeros. This was used for most of the designs in Fig. S10, all RSV-F and ACE2 designs except those noted below, and the carbonic anhydrase designs.
- An unpublished “RF-Nov05-2021” with multiple architectural improvements adopted from AlphaFold2: 1) use of a “ExtraMSAStack” module instead of perceiver architecture, 2) use of triangular multiplicative updates and triangular self-attention for pair feature updates, 3) communication between 1D, 2D, and 3D tracks through attention biasing, and 4) use of recycling that executes the network multiple times with the updated input embeddings based on outputs from the previous cycle. In addition to the PDB, the model was also trained on 12 million Alphafold-predicted models generated by Facebook [(*64*)](https://www.zotero.org/google-docs/?TpMLAJ). The model was trained using the masked language model objective as well as distogram prediction loss and FAPE loss. This was used to generate rsvfv_hal_2 and rsvfv_hal_3 (and other designs tested together with them), the Mdm2 binder designs, and the TrkA & PD-L1 free hallucinations.

The protein structure prediction performance of each RosettaFold variant was evaluated based on CASP14 targets and 60 recently published de novo designs (not included in the RosettaFold training set) as shown in Fig. S21.

Inpainting models were fine-tuned starting from one of the pre-trained RF versions above. RF_joint_ was based on RF-Nov05-2021, and RF_implicit_ was based on RF-perceiver (see dedicated sections for precise training details). Both PDB and Facebook AF2 models were used for training.

Because RosettaFold only predicts backbone coordinates, we added sidechains to hallucination and inpainting outputs using Rosetta and refined the full-atom structure by relaxing once in torsion space with predicted pairwise restraints and once in cartesian space with only pairwise distance restraints and C𝛼 coordinate restraints. Outputs from the trRosetta-based hallucination pipeline were relaxed similarly, except a structural model was first built by minimizing against the predicted pairwise restraints because trRosetta does not directly predict 3D coordinates. The output of the final relax step is the model used for downstream analysis and further design.

### Alternate formulations of motif and scaffold losses

#### Coordinate RMSD loss

In addition to the cross-entropy motif loss, sometimes we used an additional RMSD motif loss $\mathcal{L}_{M,RMSD}$ defined as the backbone (N, C𝛼, C) root-mean-squared distance between predicted and reference motif coordinates after superposition [(*73*)](https://www.zotero.org/google-docs/?2eNvzn). While using $\mathcal{L}_{M,RMSD}$ alone did not yield as good designs as using the cross-entropy loss $\mathcal{L}_{M}$ alone, a combination of the two losses (with weights $w_{M}=1$ and $w_{M,RMSD}=0.5$) gave the best DAN-lDDT and motif RMSD.

#### KL divergence loss

In some cases we defined the hallucination loss as a Kullback-Leibler (KL) divergence rather than entropy, following previous practice[(](https://www.zotero.org/google-docs/?PneQzR)*12*[)](https://www.zotero.org/google-docs/?PneQzR). Given network predictions $p(y)$ and background distributions $q(y)$ discretized over *B* bins,

$\mathcal{L}_{H,KL}=-\sum_{y\in\left\{ d,\omega,\theta,\phi,\theta^{T}\phi^{T} \right\}} \left[ \left( \sum_{i=1}^{L} \sum_{j\neq i}^{L} (1-m_{ij})\sum_{b=1}^{B} p\left( y_{ijk} \right)\log\frac{p\left( y_{ijb} \right)}{q\left( y_{ijb} \right)} \right)/\left( \sum_{i=1}^{L} \sum_{j\neq i}^{L} (1-m_{ij}) \right) \right]$

The background distributions represent residue-residue distance and angle distributions conditioned on only sequence separation, without knowledge of the amino acid identities[(*11*, *74*)](https://www.zotero.org/google-docs/?g0Nf1U). We generated the background using a separately trained neural network (for trRosetta) or by averaging the predictions for 100 random sequences (RosettaFold). The KL hallucination loss gave generally similar results as the entropy loss, although entropy yielded designs with higher helical content.

#### Leaky entropy (free binder hallucination)

In our standard entropy loss term, the probability distributions over distances and orientations are renormalized after removing the last (>20 Å or “no-contact”) bin, and then the entropy is computed (Materials and Methods). However, for residue pairs that are not in contact (i.e. most of the probability is in the last bin), the total probability of the contact bins before renormalization is very low and probably not meaningful, and optimizing their entropy after renormalization can create instability. Therefore, we defined an alternate entropy loss where normalization is performed over all bins before taking the log (thus making it sensitive to probability “leaking” from no-contact into contact bins), but this log probability is multiplied by a probability that excludes the last bin before renormalization. This “leaky” entropy is defined as:

$$\mathcal{L}_{H,leaky}=\sum_{y\in\left\{ d,⍵,\theta,\varphi,\theta^{T},\varphi^{T} \right\}} \left[ \left( \sum_{i=1}^{L} \sum_{j\neq i}^{L} \left( 1-m_{ij} \right)H_{leaky}(p\left( y_{ij} \right)) \right)/\left( \sum_{i=1}^{L} \sum_{j\neq i}^{L} (1- m_{ij}) \right) \right]$$

where

$$H_{leaky}\left( p \right)= \sum_{k=1}^{K-1} \hat{p_{k}}\log\bar{p_{k}}$$

Here the probability outside the log is renormalized after excluding the last bin (out of K bins):

$$\hat{p}(y)=exp\left( \beta logp(y) \right)/\sum_{k=1}^{K-1} exp\left( \beta logp(y) \right)$$

and the probability inside the log is renormalized across all bins (but the last bin is excluded in the entropy calculation).

$$\bar{p}(y)=exp\left( \beta logp(y) \right)/\sum_{k=1}^{K} exp\left( \beta logp(y) \right)$$

Like our standard entropy loss, leaky entropy avoids favoring too much probability in the no-contact bin, but it more accurately scores the contact bins when their probability is low. Most of the designs in this paper are generated using standard entropy. However, we used leaky entropy to generate the free-hallucinated interface stubs, as well as the free-hallucinated PD-L1 binders. In this application, leaky entropy yields better designs than standard entropy, especially when used to compute the entropy over inter-chain residue pairs (see “Auxiliary losses” below; Fig. S17H).

### Auxiliary losses

For some problems we used additional auxiliary loss terms consisting of repulsive, attractive, radius-of-gyration, surface nonpolar, and net charge loss terms (Fig. S2):

$\mathcal{L}_{aux}=w_{rep}\mathcal{L}_{rep}+w_{atr}\mathcal{L}_{atr}+w_{rog}\mathcal{L}_{rog}+w_{surfnp}\mathcal{L}_{surfnp}+w_{nc}\mathcal{L}_{nc}+w_{H,ic}\mathcal{L}_{H,ic}$.

#### Repulsive and attractive

The repulsive and attractive losses $\mathcal{L}_{rep}$ and $\mathcal{L}_{atr}$ are partial Lennard-Jones potentials with a user-specified characteristic distance 𝜎 (Fig. S2B)[(*75*)](https://www.zotero.org/google-docs/?Cc7eTX). The potentials are a function of the distance between predicted backbone atoms of the hallucinated protein and all atoms of a user-defined binding partner, and averaged over all such pairs (Fig. S2A).

#### Radius of gyration

The radius of gyration loss $\mathcal{L}_{rog}$ is used to control the overall shape of generated proteins and to indirectly favor a well-packed core (Fig. S2B). It is defined as an exponential linear unit with a user-specified threshold *R*_0_:

$$\mathcal{L}_{rog}=\left\{ {}_{exp(R_{g}-R_{0}), R_{g}\leq R_{0}}^{R_{g}, R_{g}>R_{0}} \right\}$$

where the radius of gyration $R_{g}$ is calculated as the root-mean-squared position of the predicted C𝛼 positions $\boldsymbol{r}_{C\alpha}$:

$$R_{g}=\sqrt{\frac{1}{L}\sum_{i}^{L} \left| \left| \boldsymbol{r}_{C\alpha} \right| \right|^{2}}$$

For epitope presentation and receptor decoy hallucinations, we used the repulsive and radius-of-gyration losses, with $w_{rep}=1$, $\sigma_{rep}=4 Å$, $w_{rog}=1$, and $R_{0}=18 Å$. For binder design, we used both repulsive and attractive losses, with $w_{rep}=1$, $\sigma_{rep}=4 Å$, $w_{atr}=10$, $\sigma_{atr}=6 Å$,$w_{rog}=1$, and $R_{0}=18 Å$. The unweighted attractive loss is typically 50-100x smaller than the other loss terms, so it is given a higher weight.

#### Surface non-polar

Using only hallucination and motif losses, hallucination sometimes places hydrophobic residues at the surface of a protein (although it does not place buried polar residues); this is likely due to some of RosettaFold’s training examples being single chains extracted from homooligomeric complexes. To discourage this, we compute a loss term that is higher when there are more non-polar residues (V, I, L, M, W, F) on the surface of the hallucinated protein. First, the number of neighbors n_i of each residue i is calculated as the sum of contributions from all other residues j weighted by their distance and position in a cone around the Ca-Cb vector of i:

$$n_{i}=\sum_{j=1}^{L} 1/(1+exp(d_{ij}-m))*((cos({\pi-\phi}_{ij})+a)/(1+a))^{b}$$

This formula is based on the sidechain neighbors selector in RosettaScripts. dij and phi_ij are, respectively, the Cb-Cb distance and Ca-Cb / Ca-Cb angles between residues i and j (Fig. S2A), and m=1, a=0.5, and b=2 are tuning parameters set to their default values in RosettaScripts (<https://www.rosettacommons.org/docs/latest/scripting_documentation/RosettaScripts/ResidueSelectors/ResidueSelectors>).

The surface non-polar loss is then defined as

$$\mathcal{L}_{surfnp}=\sum_{i=1}^{L} \delta_{i}*[1-sigmoid(n_{i}-n_{0})]/\sum_{i=1}^{L} [1-sigmoid(n_{i}-n_{0})]$$

where $\delta_{i}=1$ if residue i is non-polar and 0 otherwise, and n_0 is a user-defined threshold (which we set to 2.5) representing the maximum number of “neighbors” a surface residue can have. The quantity $1-sigmoid(n_{i}-n_{0})$ ranges from 0 to 1 and is higher when a residue is closer to the surface.

#### Net charge

The net charge of a protein is important for its solubility, with neutral or positive net charge more likely to lead to aggregation (possibly due to nonspecific binding to negatively charged DNA). Therefore, we compute the net charge by summing up the number of lysines and arginines and subtracting the number of glutamates and aspartates, and applied an exponential rectified linear transform at a user-defined threshold (usually -5) so that net charge below this value has a loss of 0. Although this loss $\mathcal{L}_{nc}$ can be applied during gradient descent via our straight-through gradient approximation, it is more stable to optimize during MCMC refinement stages. We only used this loss for designs intended for experimental testing, and not when generating designs to benchmark method performance.

#### Inter- and intra-chain entropy

During (two-chain) free hallucination for binder design, we compute the entropy loss only over the binder intra-chain contacts. We then add a separate loss term for the inter-chain entropy $\mathcal{L}_{H,ic}$, which is the sum of the entropy over residue pairs that are not in the same chain. This can be either standard entropy or leaky entropy (see above); we used standard entropy for TrkA free hallucination (Fig. S17F) and leaky entropy for stub design (Fig. S18) and PD-L1 free hallucination (Fig. S17G).

### Automatic motif placement

The fixed motif-placement method described in the main methods is simple, but requires extensive sampling to identify good motif placements and iterative rounds of design to efficiently explore the search space. To avoid this sampling and iteration, we developed 2 methods to automatically place the motif during optimization.

#### Motif placement by exhaustive triplet enumeration

The first method uses a modified motif loss that rewards recapitulation of the motif in any location on the protein. Consider a motif consisting of M discontinuous segments or “contigs” being placed on a protein of length L. Exhaustive enumeration of all contig placements in all possible positions in the designed sequence would require $O\left( L^{M} \right)$ loss evaluations and is not feasible for multi-segment motifs with many contigs (large M>3). However, the M=3 case is still practically realizable, so we developed an approach which exhaustively enumerates placements for all possible contig triplets from the motif and forces placements of different triplets to be self-consistent (described below). This was achieved by developing a two-term loss function

$$\mathcal{L}_{motif}=\mathcal{L}_{sat}+\mathcal{L}_{con}$$

The first term $\mathcal{L}_{sat}$ forces recapitulation of the entire motif by averaging over $\left( {}_{3}^{M} \right)$ $\mathcal{L}_{sat}^{abc}$ scores controlling how well each individual $abc$ triplet fits into the hallucinated structure:

$$\mathcal{L}_{sat}=1/\left( {}_{3}^{M} \right)\sum_{abc\in\left\{ triplets \right\}} \mathcal{L}_{sat}^{abc}$$

Given network predictions $p(y)$, triplet-wise satisfaction scores $\mathcal{L}_{sat}^{abc}$ are calculated as a weighted average of cross entropy scores $H_{ijk}^{abc}=H_{ij}^{ab}+H_{jk}^{bc}+H_{ik}^{ac}$ for placing contigs a,b,c at positions i,j,k in the sequence:

$$\mathcal{L}_{sat}^{abc}=\sum_{i,j,k} p_{ijk}^{abc}H_{ijk}^{abc}$$

where$H_{ij}^{ab}=-\sum_{y\in\left\{ d,⍵,\theta,\varphi,\theta^{T},\varphi^{T} \right\}} logp(y_{ij}=y^{0})$, , $y_{ij}$ is the predicted distance or orientation angle between positions i and j, and $y^{0}$ is the desired value of the geometric parameter between contigs a and b. The best placement of 3 contigs is the (i,j,k) that minimizes $H_{ijk}^{abc}$. To favor emergence of a single best placement during optimization, we weight the 3-body cross entropy scores by their statistical weight:

$$p_{ijk}^{abc}=exp(-\beta H_{ijk}^{abc})/\sum_{i,j,k} exp(-\beta H_{ijk}^{abc})$$

The inverse temperature parameter $\beta$ controls the strength of constraints and is increased throughout optimization from 2 to 20.

In the triplet decomposition, it is possible that different triplets *abc* and *abd* sharing a pair of contigs *ab* may yield optimal placements $ijk$ and $lmn$such that $ij\neq lm$. To discourage this, we use a “triplet consistency” loss defined as the negative symmetrized cross-entropy between marginal probabilities of placements of a given contig pair in different triplets, averaged over all order 4 permutations of contigs a,b,c,d :

$$\mathcal{L}_{con}=-1/\left( {}_{4}^{M} \right) \sum_{a,b,c,d\in\{quadruplets\}} \frac{1}{L^{2}}\sum_{i,j} \left( p_{ij}^{ab(c)}logp_{ij}^{ab(d)}+p_{ij}^{ab(d)}logp_{ij}^{ab(c)} \right)$$

where $p_{ij}^{ab(c)}=\sum_{k} p_{ijk}^{abc}$ is the probability of placing 2 contigs a,b at positions i,j marginalized over the placements of a 3rd contig c.

During optimization, we use $\mathcal{L}_{sat}+\mathcal{L}_{con}$ instead of $\mathcal{L}_{M}$ as the motif loss term, and at the end of optimization we identify contig placements by looking for high-scoring cliques in the weighted adjacency matrix $A_{ij}=p_{ij}^{\alpha b(c)}+p_{ij}^{\alpha c(b)}+p_{ij}^{bc(a)}$ averaged over all triplets a,b,c.

#### Motif placement by greedy search

Although fully differentiable, the triplet enumeration method above requires $O(L^{3}M^{3})$ memory for a length L protein with M contigs, and only approximately computes the motif loss when $M>3$. An alternative method uses a greedy search algorithm to place the contigs and applies the exact motif loss given this placement. On each optimization step, this method will:

1. Place 2 of the contigs by computing the cross-entropy between their inter-contig geometries and network predictions at all possible starting residue numbers i and j where they can be placed, respectively. Try this for all pairs of contigs and keep the placement of the 2 contigs with the lowest score.
2. Place remaining contigs one at a time, minimizing its inter-contig cross entropy with the already-placed contigs, until all have been placed.
3. Once all contigs are placed, the standard motif loss is calculated for that placement and used to compute the gradient.

Contigs are required to remain in a user-defined order. Positions that would result in the contigs overlapping with each other or prevent the placement of the remaining contigs are scored as positive infinity. Because greedy searches can miss global optima, we added the top 3 scoring results at each step to a search tree, yielding a collection of possible contig placements.

Intuitively, this method will initially place contigs in positions that randomly happen to score well, but after a few gradient updates, these regions will match the contigs more and more and the process becomes self-reinforcing. Because this method only evaluates pairs of contigs, it requires $O(L^{2})$ memory and $O(M)$ time.

### Catalytic site scaffolding with TrRosetta & Alphafold

In initial tests, we found that only using AF to hallucinate scaffolds for keto-steroid isomerase (KSI) led to poor recapitulation of the active-site side-chains. We were only able to obtain designs that fully recapitulated the catalytic sidechain geometry when optimization was over a multiple sequence alignment rather than a single sequence; the landscape may be too rugged with the high resolution sidechain-based loss in the single sequence case. However, adopting the two-stage method with trRosetta-based losses in the 1st stage allowed accurate catalytic sites to be scaffolded (Materials and Methods). We think there are two reasons why using TrRosetta early in optimization is beneficial:

1. Using a low-resolution model early in optimization and switching to a high-resolution model is common practice to avoid local minimums and to navigate the rugged landscape. Even if the structure module is disabled and the predicted distances are used during optimization, we suspect the triangle attention in AlphaFold's evoformer and 3D track in RoseTTAFold, adds an extra physical constraint contributing to traps or local minimums in the optimization landscape. Since TrRosetta does not have this constraint it has the ability to navigate between radically structural conformations during optimization.
2. During optimization, it is not clear if the desired sidechains configuration will be achieved until the end of the trajectory. A low-resolution score function is needed to approximate the desired sidechain configuration early in the optimization. One such function is the orientation of ca-cb vector from one position and cb-ca vector of the other position. These can be defined using the interresidue orientations predicted by TrRosetta. AlphaFold's distogram predictions are a poor approximation of this, often resulting in sidechains pointing in the wrong direction, even when the cb atoms are correct.

### Training RosettaFold to perform fixed backbone sequence design (RF_implicit_)

When we first began to explore inpainting with RosettaFold, we realized that RosettaFold already has some capacity to simultaneously complete protein sequence and structure, given that, during training, some of the sequence (MSA) information is corrupted/masked. Because of this, we reasoned that, simply by improving RosettaFold’s ability to predict sequence given structure, it might *implicitly* learn to inpaint *both* sequence and structure when both are masked. This was indeed the case (Fig. S3F), although the results were of lower quality than with the *explicitly*-trained RF_joint_ model (Fig. S4E). The training of this model is described below. As with RF_joint_, structure related losses were applied over the entire predicted protein, and the sequence cross entropy loss is only applied at masked regions.

We started from a pre-trained RosettaFold model (RosettaFold perceiver, see supplementary text “RosettaFold variants” section for architectural details). This model was originally trained solely for structure prediction, on the PDB set of proteins described in [(*16*)](https://www.zotero.org/google-docs/?JQmh90). The model was trained for 300 epochs, with 21120 examples per epoch and a batch size of 64. The AdamW Optimizer with default pytorch parameters was used. The learning rate was set to 5x10^-4^, with a linear warm up for the first 16000 optimization steps, and a linear decay for 200000 optimization steps. Proteins were cropped as in [(*16*)](https://www.zotero.org/google-docs/?OvEZiQ), with a crop size of 300 residues. The number of MSA seed sequences was 128, and the number of extra MSA sequences was 2048.

After this structure-prediction training, the model was further fine-tuned for an additional 5 epochs on both sequence design and structure prediction tasks with a fixed learning rate of 5x10^-4^. In the fixed-backbone sequence design task, which comprised 75% of the fine-tuning examples, we replaced 90-100% of the sequence input with “mask” tokens while retaining the native backbone features as a template structure input [(*16*)](https://www.zotero.org/google-docs/?0oqNat). For the remaining 25% of fine-tuning examples, classic structure prediction was performed, with 15% of the MSA randomly masked, no masking of the query sequence, and inputting homolog template structures as usual [(*16*)](https://www.zotero.org/google-docs/?aQcW9m). As a control, we also started joint training from a completely untrained RosettaFold model, and saw training saturation at very poor losses on both sequence design and structure prediction (Fig. S3C). This suggests that pre-training on structure prediction was needed to achieve high performance on the sequence design task.

For all fine tuning examples, we used the following loss formula, which had increased weight on the cross entropy over sequence prediction logits compared with the original RosettaFold (*15*):

$$\mathcal{L}_{total}={{0.05\mathcal{L}}_{dist}+ 1\mathcal{L}}_{aa}+{{0.025\mathcal{L}}_{tors}+0.5\mathcal{L}}_{FAPE}+{0.05\mathcal{L}}_{bond angle}+{0.05\mathcal{L}}_{bond length}+{0.05\mathcal{L}}_{lddt}$$

Where $\mathcal{L}_{dist}$ is a cross entropy loss over the distogram and anglegram as described in [(*15*)](https://www.zotero.org/google-docs/?mfQJT9), predictions $\mathcal{L}_{aa}$ is a cross entropy loss over any masked positions in the input MSA, $\mathcal{L}_{tors}$ is a cross entropy loss on binned backbone dihedral angle predictions, $\mathcal{L}_{FAPE}$ is a backbone level frame aligned point error [(*18*)](https://www.zotero.org/google-docs/?rLQWe8), with a relu cutoff of 20. $\mathcal{L}_{bond angle}$ is calculated as $\mathcal{L}_{bond angle} = \sum_{i=1}^{L} \sqrt{{(\hat{\theta}_{{Ca}_{i},C_{i},N_{i+1}} - \theta_{{Ca}_{i},C_{i},N_{i+1}})}^{2}} +\sqrt{{(\hat{\theta}_{C_{i},N_{i+1},{Ca}_{i+1}} - \theta_{C_{i},N_{i+1},{Ca}_{i+1}})}^{2}}$where $\hat{\theta}_{a_{i},b_{j},c_{k}}$is the planar angle between atoms *a*, *b*, and *c* from residues *i*, *j*, and *k* (respectively) resulting from a backbone prediction, $\theta_{a_{i},b_{j},c_{k}}$ is the ideal planar bond angle between those atoms, and *L* is the number of amino acids in the protein. $\mathcal{L}_{bond length}$ is calculated as $\mathcal{L}_{bond length} = \sum_{i=1}^{L} \sqrt{{(\hat{D}_{C_{i},N_{i+1}} - D_{C_{i},N_{i}+1})}^{2}}$where $\hat{D}_{a_{i},b_{j}}$ and $D_{a_{i},b_{j}}$ are the predicted and ideal covalent bond lengths between atoms *a* and *b* from residues *i*, and *j*, respectively. $\mathcal{L}_{lddt}$ is the lDDT loss as calculated in [(*15*)](https://www.zotero.org/google-docs/?ZnwJUU).

This version of RoseTTAFold did not have latent information recycling like AF2 or newer versions of RoseTTAFold do, but we found that recycling the predicted structure and inputting it again as a template without masking, along with inputting the original masked sequence yielded improved outputs. Therefore, for most inpainting problems using RF_implicit_, we ran 5-15 iterations of the aforementioned recycling to get a final optimized design. If there is a functional motif whose structure must be maintained, we include the native structure of this motif as an additional template input for each iteration so as to encourage the model to respect its geometry. A single design of 100 amino acids in length, using 10 iterations of inpainting, takes 5.3 seconds on a GeForce RTX 2080 GPU.

### Comparing the performance of different methods in a benchmarking test

To allow comparison both between hallucination and inpainting (Fig. S5) and between different RF_joint_ training regimes (Fig. S4D-E), we established a benchmarking test designed to test motif scaffolding. From the set of 34 proteins listed above, we selected those without missing internal residues, and, for each protein and each mask length (10, 20, 30, 40, 50 or 60 residues), masked (up to) 20 random non-redundant regions. The networks were then tasked with replacing these masked residues with protein that would support the structure of the unmasked protein (the “Motif”). For each protein and mask window size, the median (of the up to 20 designs) AlphaFold pLDDT in the replaced region (i.e. how confident AlphaFold is with the region that has been built) and the AlphaFold RMSD of the “motif” to the corresponding region in the input crystal structure was compared.

### Benchmarking AlphaFold performance

#### Rosetta ab initio folding

To compare AlphaFold structure predictions on single-sequence inputs with energy-based structure prediction (Fig. S7E-G), we collected the experimentally determined structures of 34 de novo designed proteins that were released in the PDB after April 30th, 2018, the cutoff date for the AlphaFold training set. The single sequences were input to AlphaFold and Rosetta *ab initio* folding [(*13*)](https://www.zotero.org/google-docs/?NnFxhG) and the RMSD to the true structure was calculated. The p_near_ metric, a summary statistic of how strongly a design model is encoded by its sequence, was calculated as in [(*13*)](https://www.zotero.org/google-docs/?e6l9bU). For NMR structures, the minimum RMSD across all states was recorded. Similarly, the minimum RMSD across all five AF models was recorded. The PDB accession codes of the 34 structures are: 2KL8, 5UOI, 5UP1, 5UP5, 5UYO, 5VLI, 6E5C, 6LLQ, 6MSP, 6W2R, 6W2V_A, 6W2V_B, 6W2W, 6W3F, 6W3G, 6W3W, 6W40, 6WI5, 6WMK_A, 6WMK_B, 6X9Z, 6XH5, 6XI6, 6YWC, 6YWD, 7JZM, 7JZU, 7K3H, 7KUW, 7M0Q, 7MWQ_A, 7MWQ_B, 7MWR_A, 7MWR_B. (Accession codes with a trailing “_A” or “_B” denote using chain A or B from that structure, as it had two different *de novo* structures.)

#### Single sequence predictions using AlphaFold

To evaluate the ability of AlphaFold to predict the structure of proteins using single sequences, we predicted the structure of 153 structurally validated natural proteins and 86 structurally validated *de novo* proteins (Fig. S7). A 32-protein subset of the *de novo* set (plus 20 unpublished structures) was also used for the inpainting benchmarks in Fig. S3D-F.

The list of natural proteins by PDB accession code is: 1a2y_C, 1a32_A, 1aaj_A, 1acf_A, 1agy_A, 1b0b_A, 1bk2_A, 1bkr_A, 1bm8_A, 1cc8_A, 1cei_A, 1ctf_A, 1elw_A, 1enh_A, 1ew4_A, 1ez3_A, 1fna_A, 1fzy_A, 1gou_A, 1h4a_A, 1h4y_A, 1hz6_A, 1i27_A, 1i2t_A, 1ifb_A, 1ifc_A, 1igd_A, 1iib_A, 1iu1_A, 1iul_A, 1iz6_A, 1jbe_A, 1kaf_A, 1kf5_A, 1khy_A, 1kmt_A, 1l3k_A, 1l8r_A, 1lis_A, 1lou_A, 1lu4_A, 1luz_A, 1mjc_A, 1mn8_A, 1nps_A, 1o8x_A, 1opd_A, 1pgx_A, 1poh_A, 1prq_A, 1r69_A, 1r6j_A, 1r77_A, 1row_A, 1sau_A, 1sen_A, 1su9_A, 1t2i_A, 1t2p_A, 1t3x_A, 1t3y_A, 1tg0_A, 1tig_A, 1tsf_A, 1ttz_A, 1tud_A, 1tul_A, 1ubi_A, 1ugh_A, 1unq_A, 1uow_A, 1vcc_A, 1vkk_A, 1wdv_A, 1wlz_A, 1x6x_A, 1xmk_A, 1xmt_A, 1yn3_A, 1z2u_A, 1zlm_A, 1zma_A, 1zuu_A, 2a28_A, 2acy_A, 2b29_A, 2bf5_A, 2chf_A, 2cxd_A, 2dfb_A, 2dyj_A, 2fe5_A, 2fi1_A, 2fwh_A, 2g6f_A, 2gzv_A, 2h28_A, 2h3l_A, 2he4_A, 2hhg_A, 2i24_A, 2i4a_A, 2i4s_A, 2iay_A, 2ic6_A, 2icp_A, 2igd_A, 2j5y_A, 2jek_A, 2nqw_A, 2nr7_A, 2nsz_A, 2nt4_A, 2nwd_A, 2oml_A, 2oss_A, 2pcy_A, 2ppp_A, 2qjz_A, 2qsk_A, 2qy7_A, 2r2z_A, 2ra9_A, 2re2_A, 2v1m_A, 2ve8_A, 2vq4_A, 2vwr_A, 2wwe_A, 2x35_A, 2y4x_A, 2y72_A, 2z0t_A, 2zib_A, 2zxj_A, 3b79_A, 3co1_A, 3cx2_A, 3d4e_A, 3dke_A, 3ess_A, 3ey6_A, 3f2z_A, 3fk8_A, 3gbw_A, 3hp4_A, 3hyn_A, 3ich_A, 3klr_A, 3nbm_A, 3q6l_A, 4lzt_A, 4m3s_A

The list of *de novo* proteins is: 1QYS, 2KL8, 2KPO, 2LN3, 2LTA, 2LVB, 2N2T, 2N2U, 2N3Z, 2N76, 3R2X, 4EEF, 4KY3, 4KYZ, 4UOS, 5BVL, 5CW9, 5CWB, 5CWC, 5CWD, 5CWF, 5CWG, 5CWH, 5CWI, 5CWJ, 5CWL, 5CWO, 5KPE, 5KPH, 5L33, 5TPH, 5TPJ, 5TRV, 5TS4, 5U35, 5UOI, 5UP1, 5UP5, 5UYO, 5VID, 5VLI, 5VMR, 6CZG, 6CZH, 6CZI, 6CZJ, 6D0T, 6DG6, 6DKM_A, 6DKM_B, 6DLM_A, 6DLM_B, 6E5C, 6LLQ, 6MRR, 6MRS, 6MSP, 6NUK, 6W2R, 6W2V, 6W2W, 6W3D, 6W3F, 6W3G, 6W3W, 6W40, 6WI5, 6X1K, 6X9Z, 6YWC, 6YWD, 7BPL, 7BPM, 7BPN, 7BPP, 7BQB, 7BQC, 7BQD, 7BQE, 7BQM, 7BQN, 7BQQ, 7BQR, 7BQS, 7JZM, 7JZU

### RSV-F site V immunogen design

#### Hallucination

For the first round of experimental testing and the distributions of metrics in Fig. S8, 600 designs were hallucinated using 600 steps of gradient descent, repulsive loss (𝜎=3.5 Å, weight = 2), and rog loss (threshold = 16 Å, weight = 1), and motif from 5tpn chain A residues 163-181. From this, 7 designs with AF pLDDT > 80, AF motif RMSD < 2 Å were chosen for experiments. A subset of these designs were “refined” using a greedy mutational walk with Alphafold2: all point mutants are predicted by AF, the one with the best pLDDT is kept, and the procedure repeated, until pLDDT ceases to improve or 5 mutations have been made. From the original hallucinations and AF-refined designs, 7 final designs were selected for testing. The successful binder rsvfv_hal_1 has 5 mutations that were introduced by the AF greedy refinement step. Two of the designs were solubly expressed and one design, rsvfv_hal_1, bound the antibody with a *K*_D_ of 0.9 μM (Fig. 2C, Fig. S11A) and had a melting point of 78°C by circular dichroism (CD) spectroscopy (Fig. S11B). The design model for rsvfv_hal_1 had solvent-exposed hydrophobic residues, so we generated another round of designs after adding loss terms penalizing surface hydrophobics and favoring net negative charge (see above).

For the 2nd round of experiments, designs were made using a multi-stage pipeline. Initially, 10,000 hallucinations were generated with the settings above, using either the motif above or with an additional strand (chain A 163-191) to support the motif. 577 hits with AF pLDDT > 75, motif AF-RMSD < 1.2 Å and radius of gyration < 16 Å were selected to serve as “seeds” for further refinement and diversification. Starting from each seed, multiple trajectories of 300-1000 MCMC steps were run with surface nonpolar (weight 1) and net charge (target charge -7, weight 0.02) losses. Designs were filtered on AF pLDDT, AF motif RMSD, and SAP score and used to seed additional rounds of MCMC. Finally, designs were subjected to 20-100 steps of MCMC with two-chain hallucination to eliminate any side-chain clashes with the antibody from residues outside the epitope motif; these runs were kept short to avoid creating unwanted positive interactions. A total of 13,157 hallucinations were generated over 9 rounds of hallucination, and the 30 best designs (AF pLDDT > 85, AF motif RMSD < 0.65 Å, radius of gyration < 16 Å, SAP score < 35, net charge < -5, Rosetta score/residue < -3, Rosetta ddG < -10) were chosen for testing. Rosetta ddG was calculated after superimposing the design on the native motif in complex with hRSV90 antibody minimizing sidechains in Rosetta. Of 30 selected designs from the second round, 17 expressed in E coli, 3 were monomeric, and 2 designs, rsvfv_hal_2 and rsvfv_hal_3, bound hRSV90 with *K*_D_’s of 1.0 μM and 1.3 μM (Fig. 2C, S11; the *K*_D_ values are likely under-estimated due to lack of saturation of the binding curves).

#### Inpainting

For inpainting of RSV-F site V immunogens, designs were initially scaffolded from either just the native motif (A 163-181) or the native motif and an adjacent strand (A 184-191). In the latter case, the additional strand was either connected using the native residues (A 182-183), or RF_joint_ was allowed to redesign the connection. RF_joint_ was also allowed to redesign the sequence of non-interface residues. Different combinations of lengths to inpaint were randomly sampled to yield a diversity of solutions.

Given the small size of the input motif, it was unsurprising that many of the outputs had poor (< 80) AlphaFold pLDDT, despite being predicted to scaffold the motif accurately. We therefore devised a refinement protocol, where the best topologies from the first round were resampled by providing some proportion of their inpainted residues (10, 25 or 50%, with sequence masked) to the network during inpainting, along with the native motif. This yielded outputs with similar topologies but better AlphaFold confidence metrics. 254 designs passed filters:

- AF pLDDT > 80
- AF motif RMSD < 1 Å
- Net charge < 20
- Rosetta ddG < -7
- SAP score < 38
- Rosetta score/residue < -2.5

These were subsequently structurally clustered to remove designs with TM-scores > 70 between designs, to yield the final set of 56 that were ordered and experimentally characterized.

### Di-iron binding protein design

#### Hallucination

800 hallucinations were generated using the reference PDB and motif residues shown in Table S1. Many outputs had good pLDDT and AF-RMSDs over the motif, but all the designs contained buried polar residues interacting with the metal-coordinating histidines and aspartates/glutamates (Fig. S12). We hypothesize that these residues were generated by hallucination because RosettaFold does not explicitly model metal ions, and therefore tried to satisfy the polar groups of the metal-coordinating sidechains using additional hallucinated sidechains. Although this ability could have interesting potential applications in designing buried polar networks[(*76*)](https://www.zotero.org/google-docs/?gNfgZz), we did not pursue these designs further here.

#### Inpainting

The input motif we sought to scaffold was extracted from bacterial cytochrome b-1 (PDB accession 1BCF), and comprised four approximately parallel helices (residues A18-25, A47-54, A92-99 and A123-130, harboring motif residues Glu^A18^, Glu^A51^, His^A54^, Glu^A94^, Glu^A127^, His^A130^. Eight potential looping orders were inpainted (Fig. S13B), randomly sampling connecting lengths between helices of 16-30 residues, with 8-15 residues inpainted at the N- and C-termini. For each looping order, 500 designs were generated.

While confidently predicted by AlphaFold to scaffold the motif, we noticed that some designs had a higher-than-ideal number of surface hydrophobic residues (as assessed by SAP units [(*65*)](https://www.zotero.org/google-docs/?jxwtsf)). Given the ability of RF_joint_ to design sequence-given-backbone, for some designs, we used RF_joint_ to modestly redesign the sequence to reduce the SAP score. Specifically, we redesigned hydrophobic surface residues to reduce the predicted aggregation propensity (given either the AlphaFold or RF_joint_ model as backbone-input).

The following filters were used for filtering the inpainted designs:

- AlphaFold mean pLDDT > 80
- AlphaFold pTM score > 0.7
- RMSD of AlphaFold-predicted motif to native < 1 Å
- Net charge between -25 and -5
- Surface hydrophobicity (SAP units) < 40 (for designs without surface-redesign) or < 34 (for designs with surface redesign)
- Rosetta Iron-binding energy of at least one site < -2.4

We experimentally tested 96 inpainted designs and found that 76 showed clear soluble expression. As it was impractical to characterize all 76, we chose the 12 most highly expressed, to assess metal binding by measuring the spectroscopic shift in Co^2+^ absorbance. 8 of the 12 designs displayed a spectroscopic shift at wavelengths consistent with coordination of Co^2+^ when incubated with CoCl_2_, and the 3 with the largest shifts were characterized further (dife_inp_1-3, Fig. 3B, S13E). It is likely that many of the other uncharacterized designs also could fold as intended and bind iron.

### EF-hand design

#### Hallucination

For the hallucinated Ef-hand designs tested, for single design trajectory we used 400 steps of gradient descent with an amino acid length of 100. We either used one EF-hand domain or two. We therefore used chain A 19-33 as the contig for 1-motif hallucination or in addition chain A 55-68 for 2-motif hallucination from the native (PDB ID 1PRW). The following amino acid positions from the native were forced during design 20D, 22D, 23G, 24D, 25G, 26T, 27I, 28T, and 31E. For 2-motif hallucination we also forced positions 56D, 58D, 59G, 60N, 61G, 62T, 63I, 64D, and 67E. Arginine, cysteine, and histidine were excluded from the amino acids the network was allowed to use during design. The losses used were cce (weight of 1), entropy (weight of 1), and net charge (weight of 0.5). The contig regions were placed randomly along the hallucinated proteins. Designs were filtered by AlphaFold mean pLDDT > 82, RMSD of AlphaFold-predicted motif to native < 2 Å, and RMSD of AlphaFold-predicted design to Rosettafold design < 2 Å.

#### Inpainting

The 55 inpainted EF-hand designs tested experimentally contain 51 designs from RF_joint_, and 4 designs from RF_implicit_.

For RF_joint_ designs, we began with 18,000 inpainted designs: 9,000 using native 1PRW as an input template and 9,000 from a version of 1PRW where the backbone is identical but the sequence contains a K30W mutation. In all designs, we combinatorially sampled template inputs that contained

- 5-20 masked residues at the N terminus, followed by residues A16-35 from 1PRW
- 10-25 masked residues between A35 and domain A52-71 from 1PRW
- 5-20 masked residues after A71

We chose to inpaint the second set of 9,000 off of the K30W mutant because the downstream functional assay (tryptophan-enhanced fluorescence) requires tryptophan to be near the ion binding site, and we reasoned that final designs might be higher quality if the model was conditioned on a TRP residue in its input, rather than retrospectively making a TRP mutation on an unconditioned design. The AF2 pLDDT distributions for these two sets of 9,000 designs were nearly identical (mean 77 vs 76), and their motif AF-RMSD distributions were also similar. Given this, we reasoned that a K30W mutation likely would have minimal effect on a design’s AF2 prediction metrics (especially given it is a surface mutation in the design). Thus for any designs which passed filters (see below) but were *not* conditioned on the K30W mutation, we manually added the mutation without further calculation.

We filtered this initial set of 18,000 by AF2 pLDDT > 80, and the individual EFhand domain AF-RMSDs both being < 1. This yielded 1496 sequences, all of which now had the K30W mutation discussed above. We next created two mutants for each of these sequences to add a second TRP near the binding sites - T26W and F65W (numbering with reference to 1PRW). We then used AF2 to predict the structure of all mutants to ensure the addition of a second TRP was not deleterious for a design’s AF2 metrics. Using a filter of AF2 pLDDT < 83.7, AF-RMSD of both domains individually < 1.0, and SAP score < 36, we filtered this set of 2992 designs to the final set of 51 for testing.

For 4 RF_implicit_ designs, we started from two hallucinated designs which initially scaffolded the EFhand motif(s) from 1PRW (Table S2), denoted here as *EFhand_hal_A* and *EFhand_hal_B*.

We inpainted 300 designs seeding off of *EFhand_hal_A* by combinatorially sampling template inputs that contained

- 5-13 masked residues at the N-terminus, followed by residues A11-30
- 15-24 masked residues between residue A30 and domain A50-81
- 4-8 residues after residue A81

We inpainted 300 designs seeding off of *EFhand_hal_B* by combinatorially sampling template inputs that contained

- 0-4 masked residues at the N-terminus, followed by residues A7-17
- 13-28 masked residues between residue A17 and domain A31-55
- 6-16 masked residues after residue A55

Designs were filtered using AF2 pLDDT > 80 and backbone RSMD between the AF2 prediction and the native 1PRW EF-hand on at least one of the motifs. We arbitrarily chose 1 design that passed these filters from each of the two sets of 300 designs. For both proteins, two mutants were created. For both mutants, the K30W (numbering with respect to 1PRW) mutation as seen above was made. Then the T26W mutation was made for one mutant, and the F65 mutant mutation was made for the other. This process yielded 4 tested designs, one of which showed terbium binding activity in the yeast display terbium binding assay (Fig. S14, *EFhand_inp_2*).

### PD-L1 binder (PD-1 mimetic) design

We used hallucination and inpainting to scaffold a 2-segment beta-sheet motif from the high-affinity consensus (HAC) PD-1 interface toward PD-L1 (5IUS chain A residues 63-82, 119-140) [(*15*)](https://www.zotero.org/google-docs/?jmcOAW) Given the immunoglobulin-like topology of PD-1, these 2 segments do not have nearby N- and C-termini and therefore cannot easily be linked by a short hairpin; therefore, it is non-trivial to scaffold them into any fold other than their native immunoglobulin.

#### trRosetta hallucination

We used trRosetta to hallucinate 100,611 PD-1 mimetic designs and selected a subset of 66,501 with DAN-lDDT > 0.6 and interface RMSD < 1.5 Å to sequence-design using the Rosetta FastDesign mover with layer design and fragment-based PSSMs to constrain amino acid choices in the protein. We also constrained interface residues (chain A 64, 66, 68, 70, 73 -75, 77-78, 81, 85, 89-91, 124, 126, 128, 132, 134, 136, 139) to only repack (keeping only native amino acids at these positions) and put harmonic coordinate restraints to these residues to ensure they didn’t move during relaxation. We then filtered designs on a panel of Rosetta- and deep-learning-based metrics (Fig. S1A): interface RMSD (backbone RMSD over 22 interface positions between trRosetta hallucination and reference structure), DAN-lddt (DeepAccNet predicted lDDT), cce10 (cross-entropy of residue-residue distances and angles of the design model to trRosetta predictions for the design sequence, filtered to pairs within 10 Å [(*13*)](https://www.zotero.org/google-docs/?6Rm0HN)), avg_all_frags (a measure of local sequence-structure agreement [(*13*)](https://www.zotero.org/google-docs/?OYQWUG)), score_res_monomer (Rosetta energy per residue), ddg (Rosetta ∆∆G of binding), and contact_molecular_surface (a measure of the interface area [(*4*)](https://www.zotero.org/google-docs/?Pr9KUp)). We selected 3,042 designs for testing which had interface RMSD < 0.8 Å and (DAN-lDDT > 0.75 or cce10 < 2.1 or avg_all_frags < 2.1) and DAN-lDDT > 0.7 and avg_all_frags < 2.5 and score_res_monomer < -2 and contact_molecular_surface > 475 and ddg < -50.

#### Inpainting

We generated 2 sets of inpainted designs: “free” inpaintings where only the binding motif was used as input, so RF_joint_ would have to generate the entire scaffold from scratch; and “guided” inpaintings where the binding motif, as well as guiding structural information input by hand, were provided. All designs were modeled in the presence of the target PD-L1, analogous to “two-chain” hallucination (Materials and Methods).

For free inpainting, we manually chose a looping order for the design to be inpainted with, starting at the N-terminus with motif segment A119-140 from 5IUS, then allowing 22-29 inpainted residues, then segment A63-82 from 5IUS, and finally 28-39 inpainted C-terminal residues. Additionally, we allowed RF_joint_ to redesign residues 67, 69, 71, 73, 75, and 77 in the input motif (i.e. mask and re-predict amino-acid identity, taking the most probable amino acid at each position, without masking structure) in case they changed from core to surface, or vice versa, after inpainting. We generated 314 designs using this approach. The successful binder pdl1_inp_1 is a refined (see below) version of a parent design from this set.

For guided inpainting, we tried to bias RF_joint_ to explore a topology of a beta-sheet buttressed by 2 helices that was observed in high-scoring hallucinations. To do this we manually placed 5 “guiding” residues in an input structure and asked inpainting to generate a design containing the interface motif which generally goes through the backbone atoms of the guide residues. 4 of the guide residues correspond to the rough location of N and C termini of two helices that might buttress the sheet. The 5th guide residue is placed in the middle of one of the buttressing helices, at an elevated distance above the interfacial beta-sheet so as to induce a bend in the helix to pack against the sheet without clashes. To obtain a diversity of designs, we sampled input coordinates for each guide residue from a uniform random sphere of radius 2 Å around its original manually chosen position, and also combinatorially sampled the lengths of the regions to be inpainted. Specifically, we combinatorially sampled the following template inputs, with each masked region being uniformly sampled from allowed window lengths:

- Residues A119-140 from 5IUS
- 4-6 masked residues between the previous segment and guiding residue 1
- 12-14 masked residues between guiding residues 1 and 2
- 4-7 masksed residues between guiding residue 2 and A63-82 from 5IUS
- 5-8 masked residues between the previous segment and guiding residue 3
- 11-13 masked residues between guiding residues 3 and 4
- 9-12 masked residues between guiding residues 4 and 5
- 0-3 masked

Given these inputs, RF_joint_ was able to generate a diverse family of PD-1 mimetics with this fold. We generated 1000 parent designs using this approach, although no descendants of these parent designs ended up having binding activity.

After initial design runs, designs with pLDDT > 80 and inter-chain PAE < 10 were refined using RF_joint_ to (1) “resample” the protein by randomly re-inpainting a fraction of the residues, (2) redesign *only* the sequence (keeping structure) of hydrophobic surface / boundary residues or (3) changing the order in which elements of the protein appear in primary sequence while keeping the overall fold of the protein (“relooping”). Combinations of (1), (2) and (3) were used for exploring near the topology proposed by inpainting initially, as well as optimizing a design for low net charge and low SAP score. We generated a total of 2,025 refinements off of the initial “free inpainting” set and 415 refinements off of the initial “guided inpainting” set, using the AF2 predictions of designs as input backbones for refinement over a maximum of 3 rounds of filtering (pLDDT > 80, inter-chain PAE < 10) and refinement. The final designs for experimental characterization were redundancy-reduced by mmseqs2 at 90% identity cutoff, and then filtered by Rosetta DDG < -30, SAP score < 40, net charge < -4, AF2 inter-PAE < 10, and AF2 pLDDT > 80. This final filtering yielded the pool of 31 tested sequences, one of which bound PD-L1 (Fig. 5A-C).

### Testing trRosetta-hallucinated PD-1 mimetics

Designs were reverse-translated and split in 2 halves to be synthesized by Twist on a 300-bp oligonucleotide chip, assembled by PCR, and transformed into yeast for homologous recombination with pETCON3. Yeast culture and sorting were performed as described in Materials and methods, using biotinylated PD-L1 (R & D Systems). A series of 4 sorts were performed: first twice at 1 μM PD-L1 with avidity, then 1 μM, and then at 100 nM, 10 nM, and 1 nM (Fig. S1B). Cells from the final sort were plated on CTUG agar plates and 56 colonies were Sanger sequenced to identify the designs. The 3 most abundant designs were validated by labeling clonal yeast cultures in a titration of PD-L1 and measuring on an Attune NxT flow analyzer (Invitrogen) (Fig. S1C). The resulting data was processed by manually choosing a FITC threshold for expression (log10 FITC > 3.2) and fitting a hyperbola $y=A\frac{x}{x+K}$ with free parameters *A, K* to the mean PE-H/FITC-H, where *A* is the maximal binding signal and *K* is the apparent *K*_d_. Plots in (Fig. S1C) are shown with data normalized to fitted *A* so all curves saturate at 1. Competition experiments with unlabeled wildtype PD-1 were performed with clonal yeast cultures of the binders in a similar manner (Fig. S1E).

### Bivalent TrkA binder design by hallucination followed by inpainting

We began the design process by aligning the structure of the TrkA minibinder bound to a single domain of TrkA (PDBID: 7N3T) to the complex of TrkA with its native ligand, nerve growth factor (PDBID: 2IFG). Having obtained the relative positions of the two minibinders in a signaling competent TrkA arrangement, we defined the functional motif as residues 5-18 on each of the minibinder chains. We carried out 600 steps of gradient descent with the usual motif and hallucination losses and forcing the native identity on motif residues 5, 6, 9, 10, 12, 13, 14, 16, 17 and 18 from both minibinder chains. To avoid clashes with TrkA, we applied a repulsive loss against the coordinates from the appropriately aligned TrkA structure (𝜎=3.5 Å, weight = 5). Because many of the residues in either of the two motif segments were further from each other than the 20 Å distogram horizon, we also found it necessary to apply a coordinate rmsd loss (weight = 1), which has no such distance maximum, to encourage the two motifs to have the correct orientation to each other. The resulting 380 designs were filtered (cce loss < 1.0, coordinate rmsd loss < 1.5 Å and entropy loss < 2.0) down to 9 seed designs. After manual inspection for designs with well-packed secondary structure elements and minimal loops, we chose to diversify one design of an elongated three helix.

To diversify the seed designs, we used inpainting to change the length and position of the two loop regions connecting each helix. First, we made 20 “jittered” structures by adding gaussian noise ~N(0,1) to “guide points” two residues inside each loop region. (Since inpainting is deterministic, this approach allowed us to sample different inpainting solutions for loops of the same length.) For each jittered structure, we inpainted the loops while varying their lengths between -3 and +7 residues of the original length, generating 1280 designs. After filtering for well folded designs (AF pLDDT > 80) that interact with TrkA (inter-PAE < 10 Å for at least one binding site), one design remained. This design and derivative mutants were assayed for TrkA binding by biolayer interferometry.

### Mdm2 binder (p53 helix scaffold) design by hallucination

We generated 6000 initial (monomer) hallucinations using the same settings as in RSV-F site V above, with the motif defined as 1ycr chain B 17-29 or 19-29. We kept native amino acids on the motif except at positions 20, 24, 25, and 28, since these faced away from the target and may need to be designed to pack against the scaffold. 28 filtered designs (AF pLDDT > 85, motif AF-RMSD < 1 Å, Rosetta score/residue < -2.5, rog < 14 Å) were used to seed two-chain hallucination runs of 300 MCMC steps each. Some of these designs were further refined in additional rounds of MCMC. Eventually 17,492 designs were generated from two-chain hallucination, which were filtered (inter-chain PAE < 7 Å, pLDDT_binder > 85, AF-Rosetta ddG < -48, SAP score < 33, target-aligned binder RMSD < 5 Å, net charge < -5) and manually inspected to identify the best designs.

### Native protein scaffold search

How necessary was it to use deep learning methods to scaffold the chosen functional sites? Without the ability to generate protein backbones, we would be limited to grafting the motifs into existing native protein backbones. To estimate the difficulty of using this non-deep learning approach, we used the Rosetta MotifGraft [(*77*)](https://www.zotero.org/google-docs/?XtH6pb) mover to search the PDB for proteins that we could potentially graft the motifs into, with the following requirements:

1. The resulting chimera does not clash with the binding target (if applicable).
2. For motifs with multiple segments, all of the starting and ending backbone atoms of the motif have a combined RMSD less than 1A to the graft sites in the native proteins. For added flexibility, each motif segment could replace a native segment up to 50 aa longer or shorter than itself. (ie - A short loop in the motif could replace a long native loop, so long as the starting and ending points were close to each other.)
3. For motifs with only a single element, the corresponding native segment be the same length and have an all backbone RMSD less than 1A. We could not filter on just the RMSD of the starting and ending points because it poorly constrains the orientation of the amino acids, resulting in non-plausible chimera junctions.

To account for sequence (and structural) redundancy, the sequences of all single protein chains in the Protein Data Bank (PDB) solved by x-ray crystallography were clustered at a 30% sequence identity threshold using mmseqs2 [(*78*)](https://www.zotero.org/google-docs/?ZypQtd) and assigned to a unique cluster. The number of suitable native scaffolds reported in Table S3 is the number of clusters that had at least one match, excluding the cluster that the motif was taken from. The frequency is that number divided by the total number of clusters in the PDB.

The number of matches is still likely an overestimate, since many of the matched native proteins are highly structurally homologous to the original structure the motif was taken from and therefore unlikely to scaffold the motif in a meaningfully different way. Additionally, there is no constraint that the matched native proteins be small or compact, an advantage in potentially downstream applications and a requirement that the hallucinated designs generally fulfill.

For the RSV-F site V motif, we performed a more detailed analysis against both the PDB100 and the AlphaFold proteomes database [(*41*)](https://www.zotero.org/google-docs/?okiQse) (Fig. S20). The motif, which contains a single contiguous segment, was searched against all possible positions in all structures (or models) in the 2 databases, and the lowest backbone RMSD was recorded for each structure. We filtered out any structure containing clashes (defined as heavy atoms closer than 2Å) to the antibodies against this motif in PDB:5TPN, as well as any whose sequence was more than 50% identical to 5TPN. Only 2 results (6w16, 5wb0) remained with RMSDs lower than our best designs (or a frequency of 2/(355712+161370) = 3.9x10^-6^ across the 2 databases), and even these are distantly related (36% identity) to 5TPN and highly related (90% identity) to each other. Before filtering out homologs and receptor clashes, we obtained 67 scaffolds in the databases (frequency 1x10^-4^) better than our best design.

## Supplementary Figures


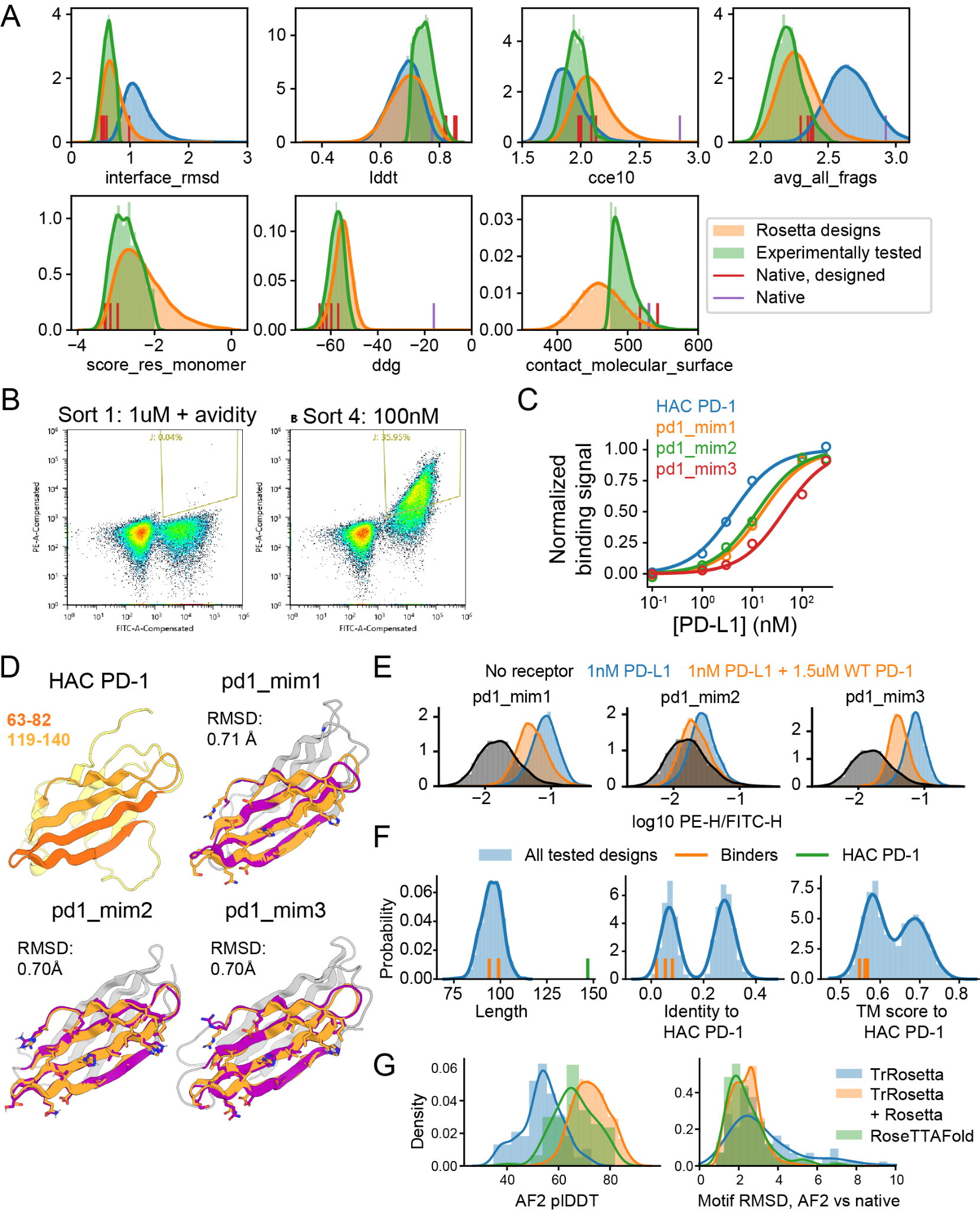


### Figure S1. trRosetta-based hallucination and testing of PD-1 mimetics

(A) Distributions of metrics for PD-1 hallucinations, Rosetta designs, and experimental library (Supplementary Text). (B) PE (binding) vs FITC (surface displayed protein) signal during FACS sorting of PD-1 mimetics. Sort 2 (1 μM PD-L1 with avidity) and 3 (1 μM PD-L1, no avidity) are not shown. (C) Binding signal (Methods) from clonal yeast cultures versus receptor concentration for HAC PD-1 and designs isolated from pooled sorting. Apparent K_d_ values in nM are: HAC PD-1: 4.10; pd1_mim1: 15.9; pd1_mim2: 12.5; pd1_mim3: 42.9. (D) Crystal structure of HAC PD-1 (discontinuous interface motif in 2 shades of orange) and design models of 3 experimentally isolated binders. “RMSD” denotes the backbone RMSD between design model and template motif at 22 interface residues (Methods). (E) Normalized PE (binding) signal for clonal yeast cultures expressing the 3 binders in the presence of receptor and receptor + unlabeled purified wildtype PD-1. (F) Distribution of sequence length, amino-acid identity to HAC PD-1, and TM-score to HAC PD-1 for the 3,038 experimentally tested designs. The values for HAC PD-1 and the 3 binders shown in (D) are plotted as vertical bars. (G) Comparison of trRosetta and RosettaFold for hallucinating PD-1 mimetics. AlphaFold predicted lDDT and motif backbone RMSD (AF model versus native motif) for hallucinations generated using trRosetta, RosettaFold, or trRosetta followed by Rosetta-based sequence design.

###

###
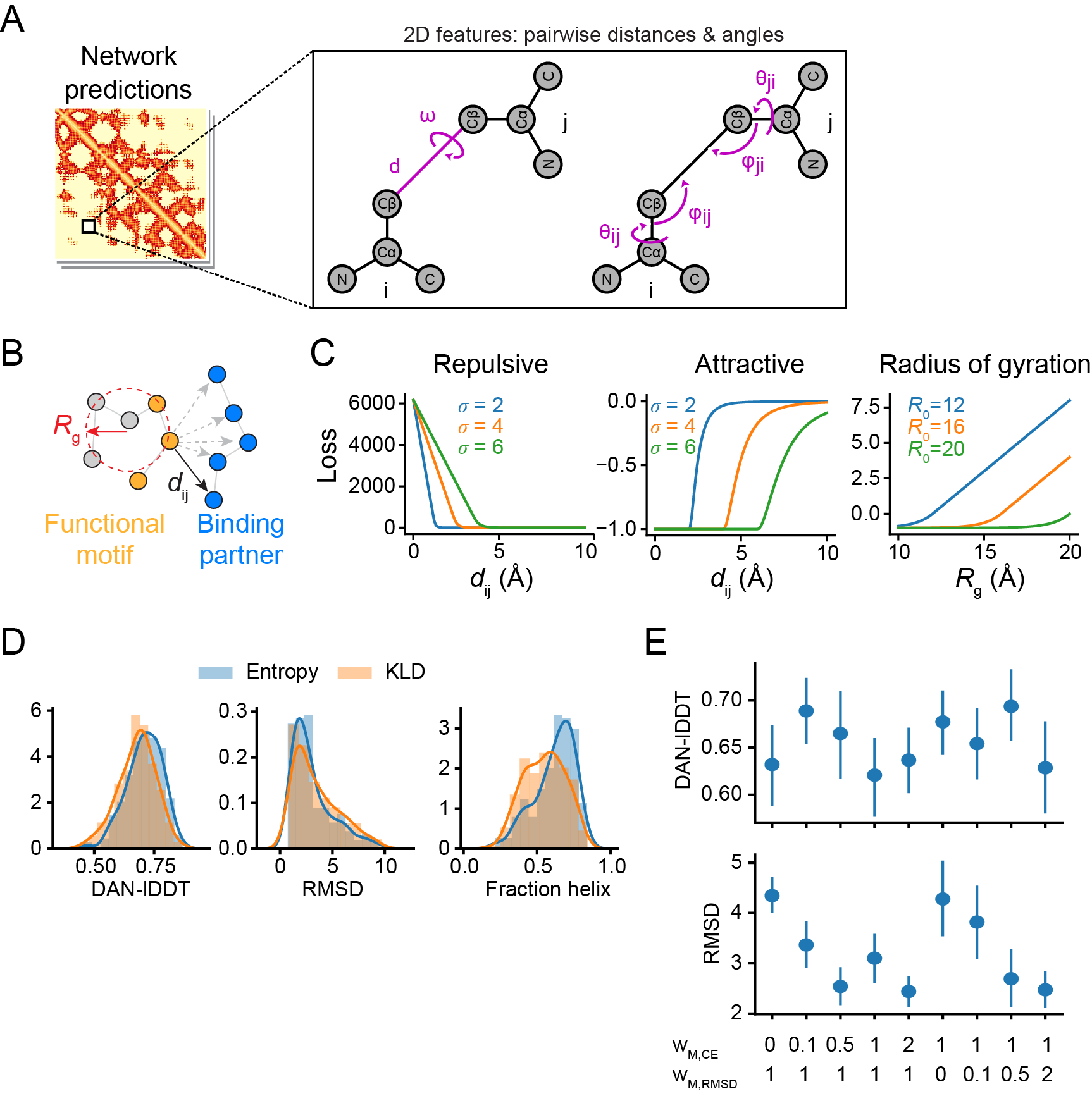


### Figure S2. Auxiliary and alternative loss terms

(A) Schematic of the pairwise distances and orientation angles whose distributions are predicted by trRosetta and RosettaFold and which are used to define the motif and hallucination losses. (B) Schematic of radius of gyration and distances used to calculate repulsive and attractive losses (Supplementary Text). (C) Functional forms of the losses. (D) Distributions of DAN-lDDT, motif RMSD, and fraction of residues that are helix for designs generated using entropy or KL divergence hallucination losses (Supplementary Text), for scaffolding a 2-segment motif from C3d (1GHQ chain A residues 104-126, 170-185). (E) DAN-lDDT and motif RMSD for the same C3d scaffolding problem as in (D), but with varying the loss term weights for the cross-entropy based motif loss (w_M,CE_) or RMSD-based motif loss (w_M,RMSD_).

###
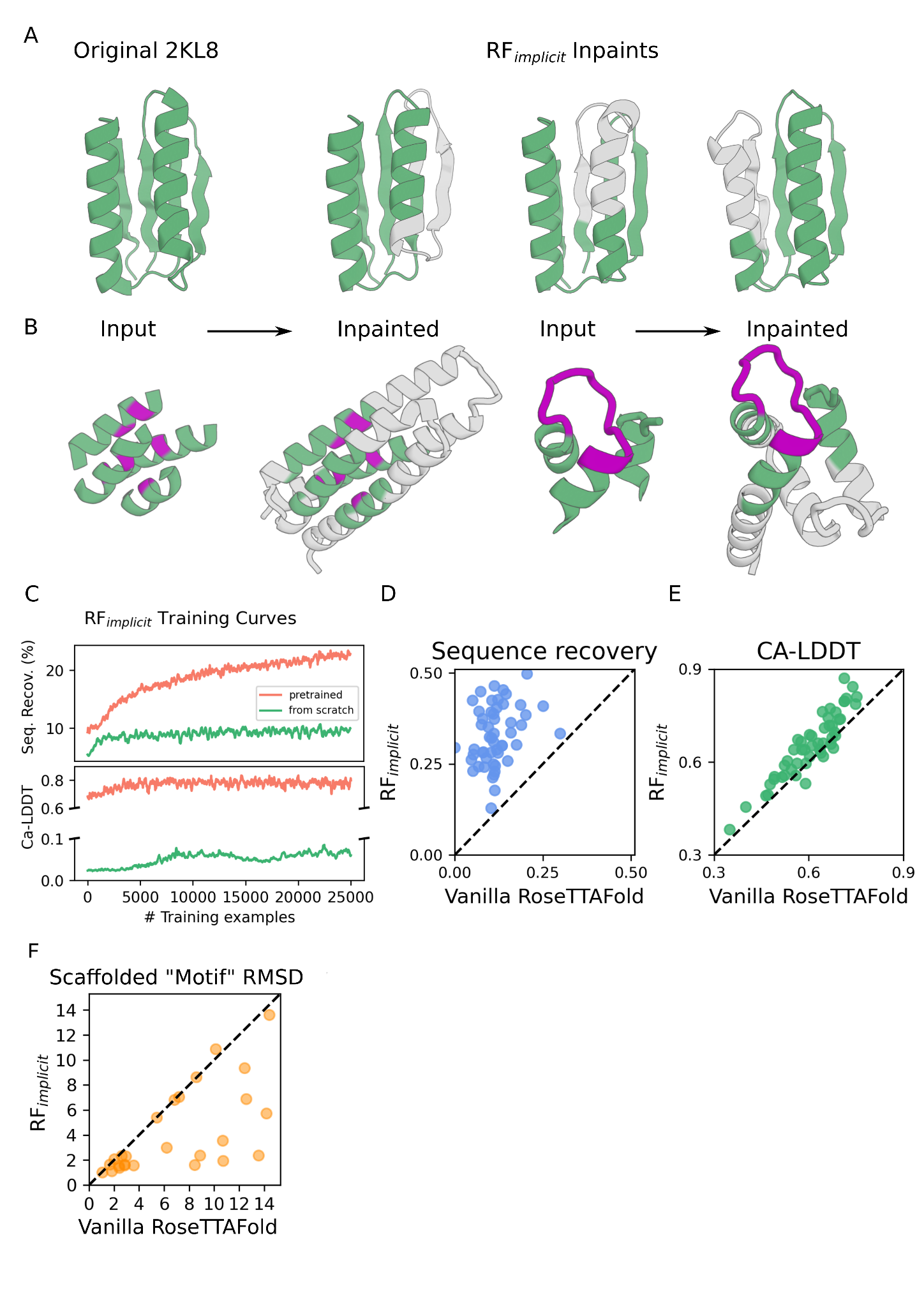


### Figure S3. Training and inpainting with RF_implicit_

(A) NMR structure of 2KL8 (left) alongside models of selected inpainting examples of the protein with a masked window size of 20 residues. Green denotes areas of sequence and structure that the network was allowed to see, gray denotes areas that the network inpainted. (B) Functional site scaffolding examples designed with RF_implicit_. (Left) AF2 prediction of design *EFhand_inp_2* from Fig. S16 scaffolding the EFhand calcium binding site, with RMSD on the motif of 0.7Å between the prediction and the native 1PRW. (Right) AF2 prediction of design *dife_impl_1* scaffolding the di-iron binding site from bacterioferritin protein 1BCF, with an RMSD on the motif of 0.5Å between the prediction and the native, and an AF2 pLDDT of 91. (C) Training curves of RF_implicit_ show that starting the training procedure from a pretrained RosettaFold model (red) results in better sequence design accuracy and structure prediction accuracy than starting from a completely untrained RosettaFold (green). (D) Sequence recovery of RF_implicit_ vs Vanilla RosettaFold on a set of 52 *de novo* proteins (Supplementary Text, “Single sequence predictions using AlphaFold”) shows RF_implicit_ outperforms the baseline model at protein sequence design. (E) CA-LDDT of RF_implicit_ vs Vanilla RosettaFold shows the model is able to retain its structure prediction capabilities on the same set of 52 *de novo* proteins even after learning protein sequence design. (F) AF-RMSD of the “motif” (unmasked) region when performing the inpainting benchmark seen in Fig. 1F-G (main text) using RF_implicit_ vs Vanilla RosettaFold, and a masked window size of 20 residues.


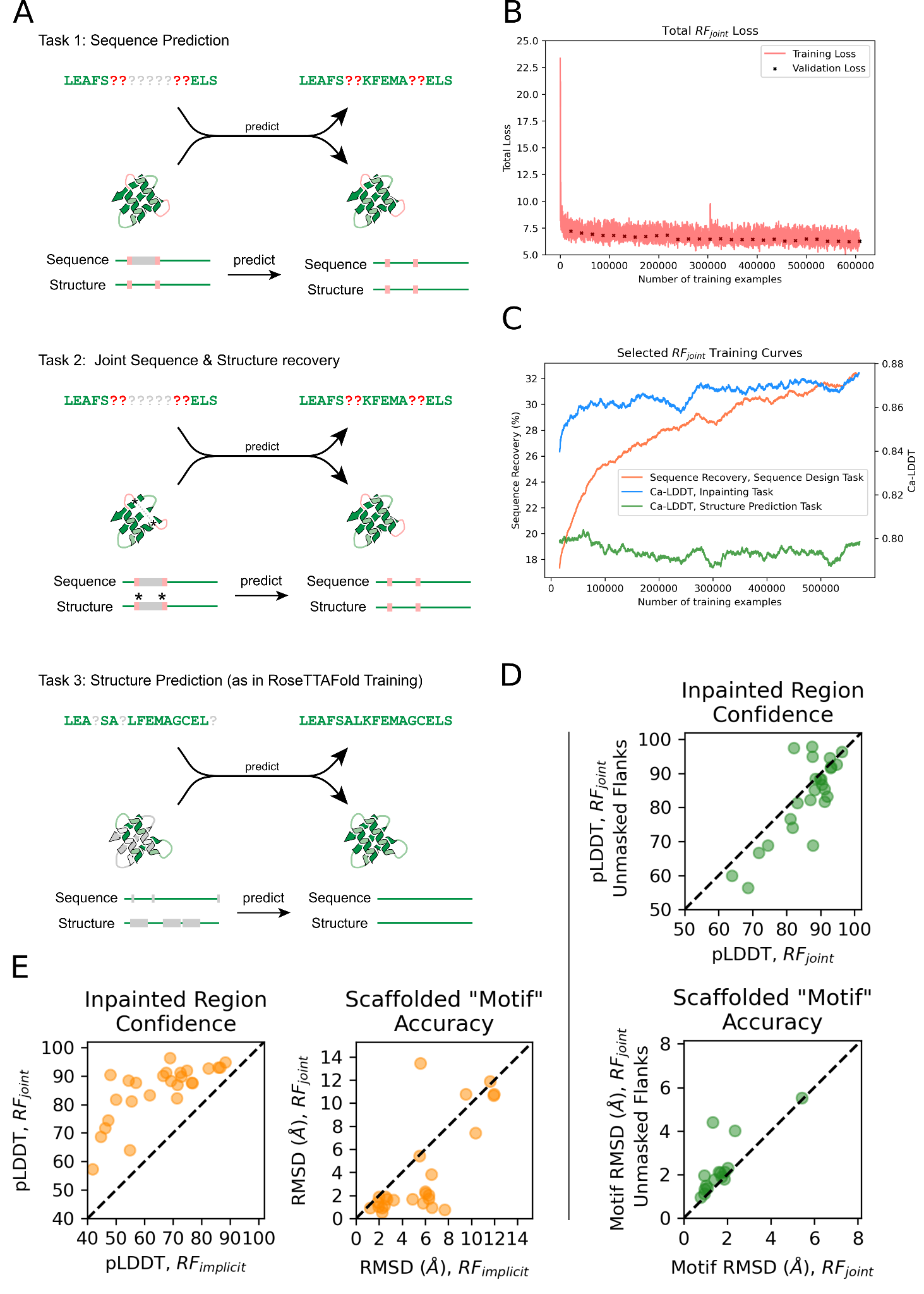


### Figure S4. Training of joint sequence-structure recovery RosettaFold.

(A) Depiction of the three tasks used to train RF_joint_, which were trained with equal likelihood (see Algorithm 1). Task 1 comprised a fixed-backbone sequence design task of a continuous segment of a given protein, without the immediate up- and downstream protein visible (see Methods). Task 2 comprised an inpainting task, where the model was tasked with predicting the sequence and structure of a continuous section of protein, also without up- and downstream protein visible. Asterisks indicate “guiding points” provided as inputs during inpainting to Task 3 is the structure prediction task originally used to train RosettaFold. (B) Training curve for RF_joint_, showing total training (red) and validation (black crosses) losses decreasing. (C) A selection of different losses associated with each of the three tasks. RF_joint_ does not severely deteriorate in its ability to predict protein structures (task 3, green line), but its ability to inpaint structure (task 2) improves dramatically (blue line). The model also learns to predict the sequence of a fixed backbone (task 1, orange line). (D) Masking out the structure and sequence of the flanking regions (depicted in (A), Tasks 1 and 2) improves inpainting performance. RF_joint_ was compared to an identically-trained model, except that flanking regions were not masked during training, on the benchmarking task described in Fig. S5. Both AlphaFold pLDDT in the inpainted region (top), and the “Motif” RMSD of the AlphaFold predictions (bottom) were marginally better for RF_joint_. (E) RF_joint_ outperforms RF_implicit_, both in terms of the AlphaFold pLDDT in the inpainted region (left), and in the “Motif” RMSD of the AlphaFold prediction (right). Graphs in D and E correspond to a masked window of 30 residues.


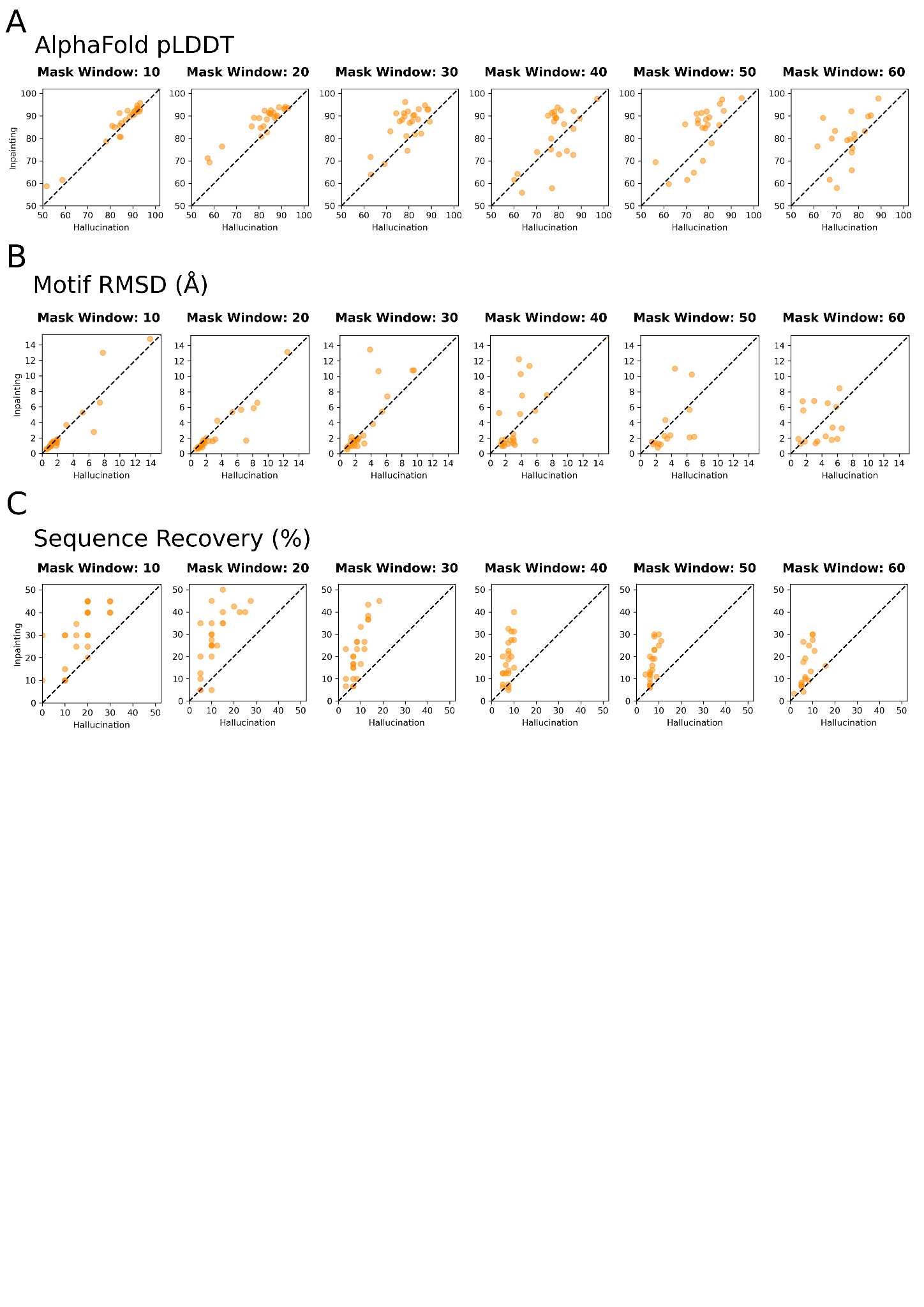


### Figure S5. Comparison of hallucination and inpainting design quality

(A) Inpainting versus hallucination AlphaFold pLDDT, as a measure of overall design quality, for various window sizes over which sequence and structure were rebuilt by both methods. Each point corresponds to a crystal structure from a benchmarking set of *de novo* proteins. (B) Inpainting versus hallucination motif AF-RMSD for the same benchmarking set. The “motif” is defined as the region of the protein that was not masked for rebuilding. (C) Percentage sequence recovery in the rebuilt region of protein, in the same benchmarking set.


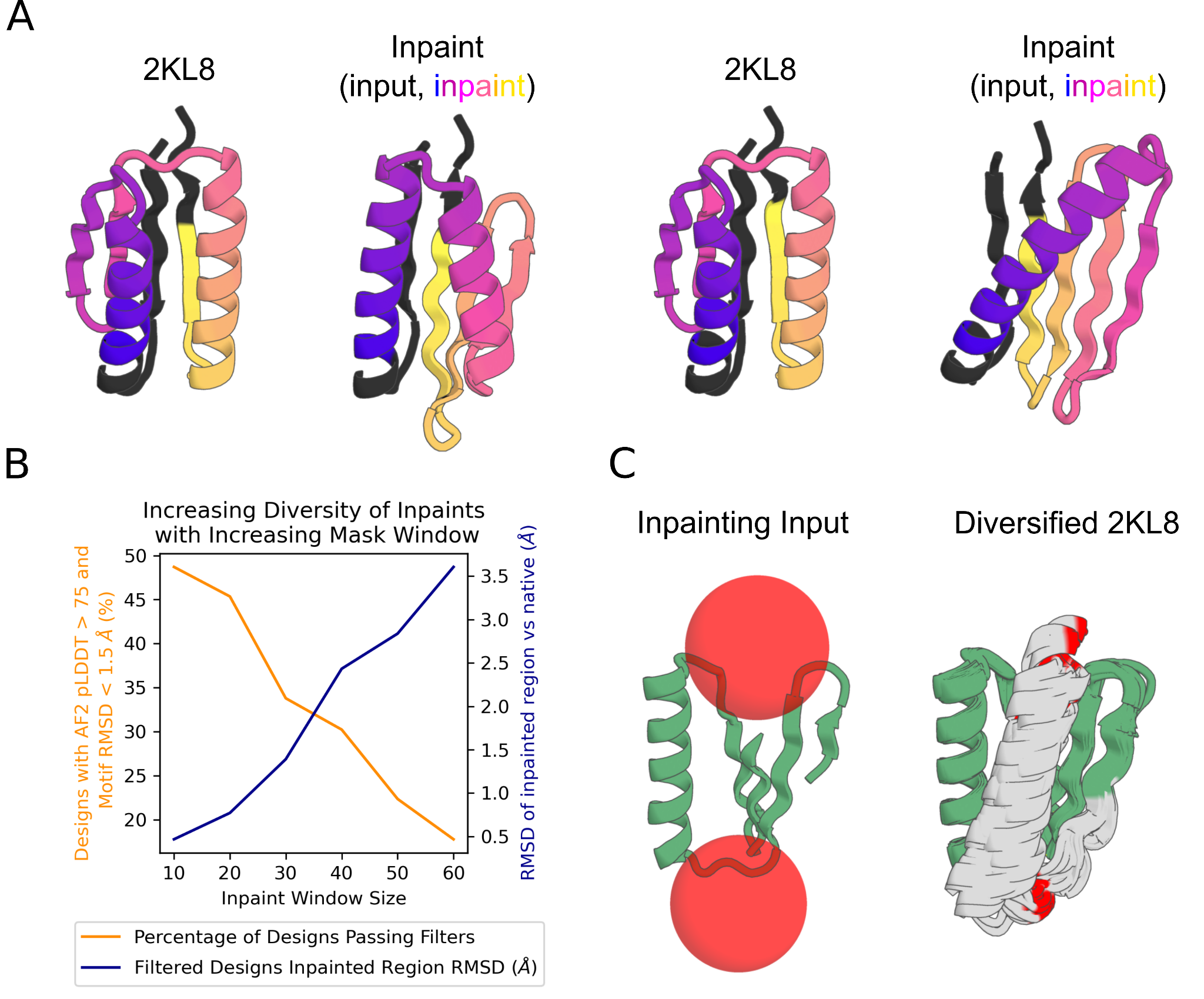


### Figure S6. Generating diversity with inpainting

(A) With a large region of structure masked, inpainting can sometimes produce confidently-predicted designs that scaffold the input motif. Two designs are shown, with the dramatically different looping order (left) or topology (right) highlighted with spectrum colors. Both designs scaffold the input “motif” (dark gray). (B) Analysis performed on the inpainting benchmarking data shown in Fig. S5. While the proportion of inpainted designs passing AlphaFold filters (> 75 pLDDT, < 1.5 Å, orange line) decreases with increasing size of the masked window, those designs that do pass filters, and thus successfully scaffold the motif, show more scaffold diversity (as assessed by AF-RMSD to the native masked region) than those designs with a smaller inpainted region (blue line). (C) Further diversity can be explicitly generated by perturbing the input coordinates. During training, RF_joint_ was trained to Cɑ-coordinates as approximate positional information (see Methods). Therefore at inference, input Cɑ-coordinates can be randomly translated (uniformly sampled from within depicted spheres, left), and the model thus outputs diverse inpainted structure (right, gray) capable of supporting the unmasked “Motif” (right, green). All designs shown in (C) have pLDDT (both total pLDDT and just in the inpainted region) > 80 and “Motif” AF-RMSD < 1.2 Å, and represent examples from each of 30 clusters (clustered at total TM score cutoff of 0.95).

###
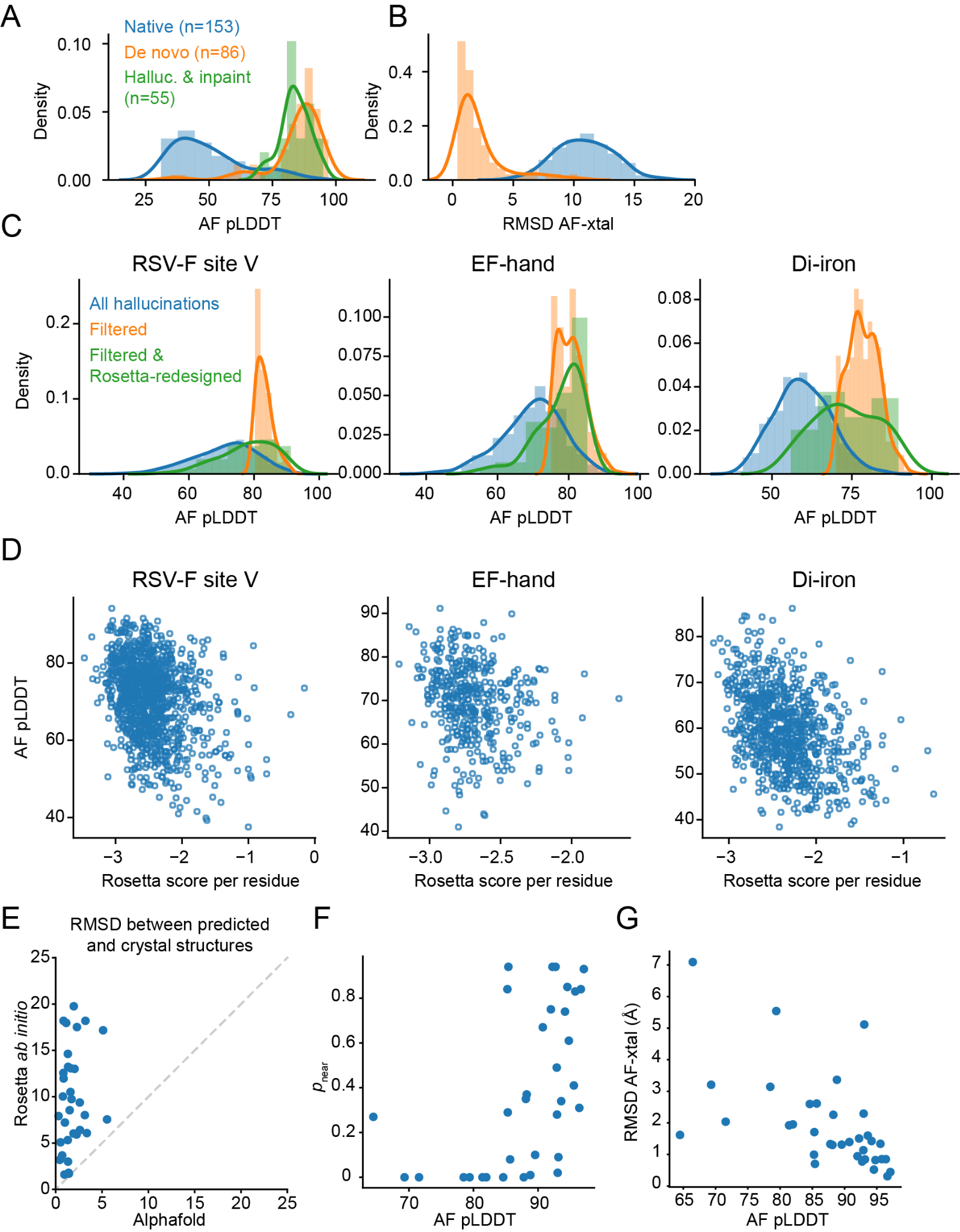


### Figure S7. Using AlphaFold for *in silico* evaluation of *de-novo*-designed proteins

(A) AF pLDDT (mean across all 5 models) using single-sequence input (no multiple-sequence alignment) for a benchmark set of 153 native proteins [(*79*)](https://www.zotero.org/google-docs/?xbnTsD), 86 structurally validated *de novo* proteins, and experimentally tested or visually displayed designs in this study. (B) RMSDs between AF predictions and crystal structures (averaged across all 5 models) for the same proteins as in (A). (C) AlphaFold pLDDT distributions of hallucinations for 3 representative design problems (blue). The designs are filtered to those with high plDDT and low motif RF-AF RMSD (orange), and then the sequence is redesigned using Rosetta Fastdesign and scored again by AlphaFold (green). (D) Scatterplots of AF pLDDT versus Rosetta score (energy) per residue, showing that AF quality estimates correlate with energy-function-based quality estimates. (E) RMSD between predicted and crystal structure via Rosetta *ab initio* (“forward folding”) versus AlphaFold for 34 *de novo* designs not in the AF training set (Supplementary Text). All predictions used only single-sequence input. (F) p_near_, a measure of the confidence of an *ab initio* prediction, versus AF pLDDT, for the de novo designs in (E). (G) RMSD between AF predictions and crystal structures versus AF pLDDT for the *de novo* designs in (E).


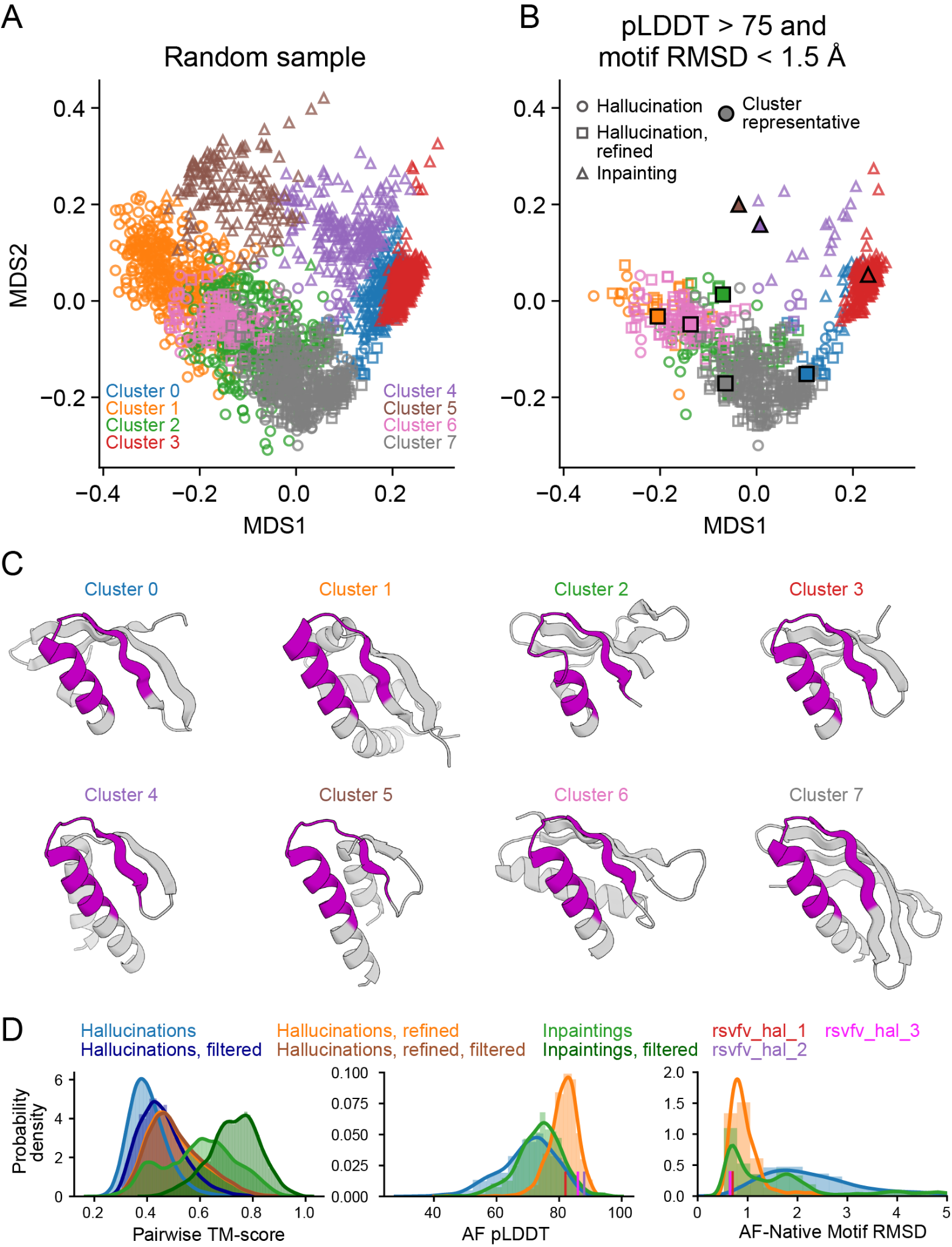


### Figure S8. Structural diversity of hallucinated and inpainted RSV-F site V scaffolds

(A) Random subsample of 1000 hallucinations, 500 refined hallucinations (Materials and Methods), and 1000 inpaintings for the RSV-F site V epitope scaffolding problem, and (B) subset of designs with AF pLDDT > 75 and motif AF-RMSD < 1.5 Å. All pairwise structural distances (1 - TM-score) were projected into 2 dimensions using classic multidimensional scaling. 8 clusters were identified using k-means, and design models of cluster representatives (black-outlined markers) with highest pLDDT are shown in (C) with motif region in purple. The number of k-means clusters was chosen arbitrarily. Inpaintings (triangles) and hallucinations (circles, squares) occupy different regions of structure space. (D) Distributions of AlphaFold pLDDT, motif AF-RMSD, and pairwise TM-scores within hallucinations, refined hallucinations, and inpaintings, either in full set or only designs with pLDDT > 75 and motif AF-RMSD < 1.5 Å (“filtered”).

###
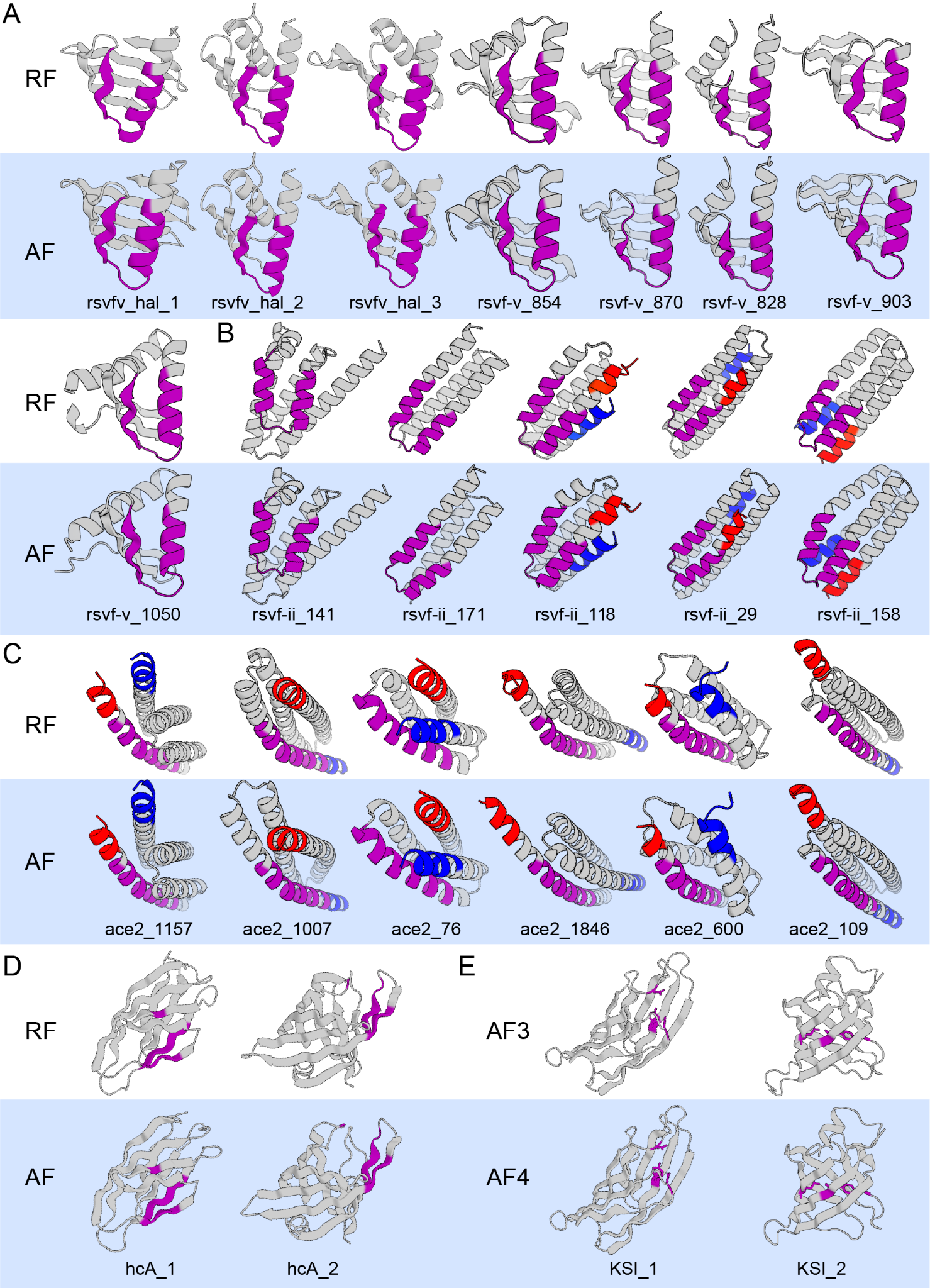


### Figure S9. RosettaFold and AlphaFold models of hallucinations

RosettaFold (RF) and AlphaFold (AF) models of hallucinations for (A) RSV-F site V and (B) site II epitope scaffolds, (C) ACE2 receptor traps, and (D) carbonic anhydrase and (E) ketosteroid isomerase (KSI) active-site scaffolds. These include the designs shown in the main figures, as well as additional designs. Functional motifs are highlighted in purple. The N- and C-termini in some designs have been colored blue and red (respectively) to highlight that hallucination can find diverse topological solutions, despite having similar overall folds. Because the KSI designs were hallucinated using AlphaFold model 3 (AF3), validation models were predicted with AF model 4 (AF4). Detailed metrics for these designs can be found in Table S2.


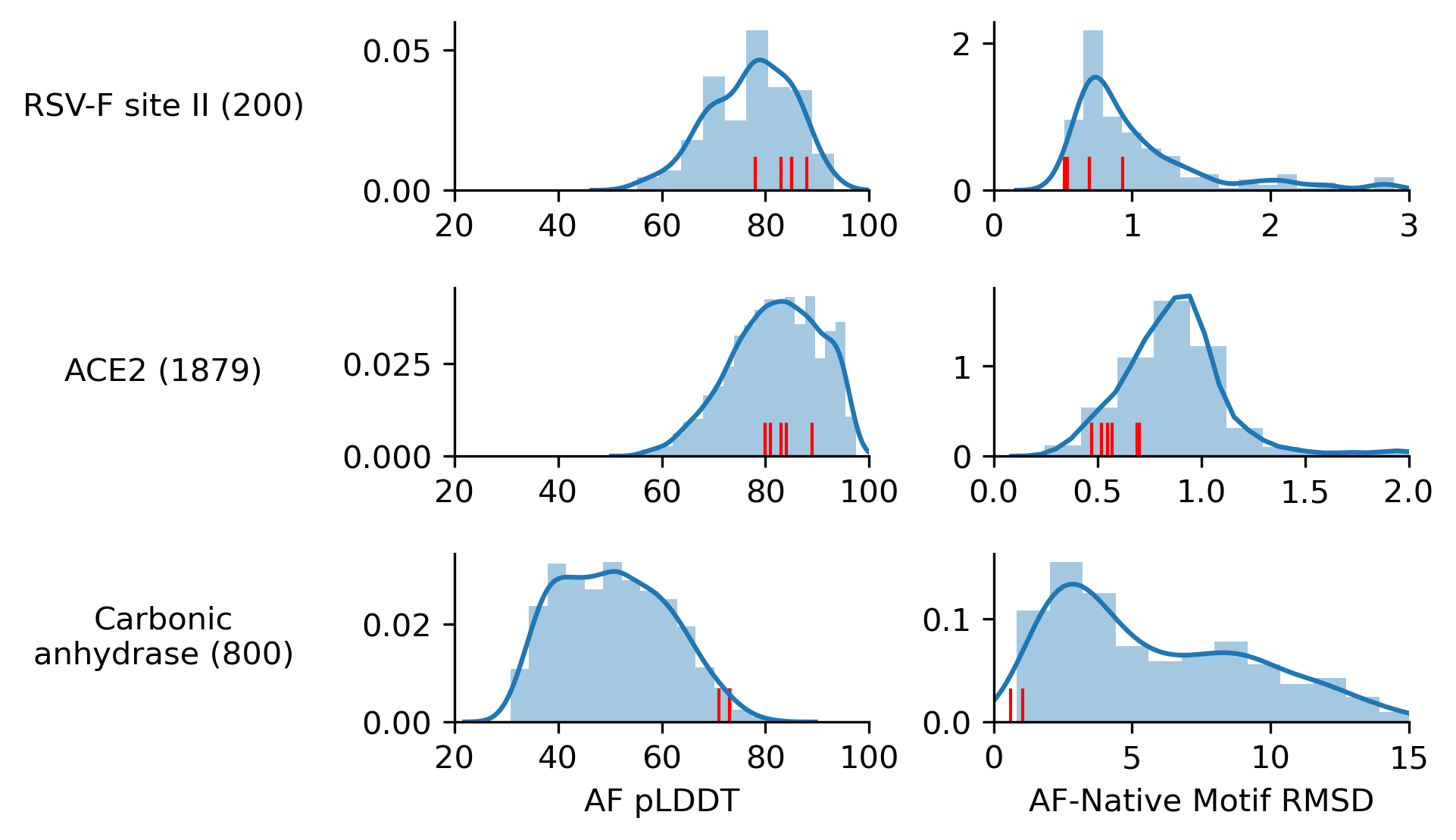


### Figure S10. Distribution of plDDT and motif RMSD of hallucinations before filtering

Distributions of (A) AlphaFold pLDDT and (B) backbone RMSD between native motif and AF predictions from hallucinated sequences, for design problems presented in Fig. 2-3. Parentheses indicate the number of designs. Red lines indicate designs filtered and chosen for display in main figures.

###
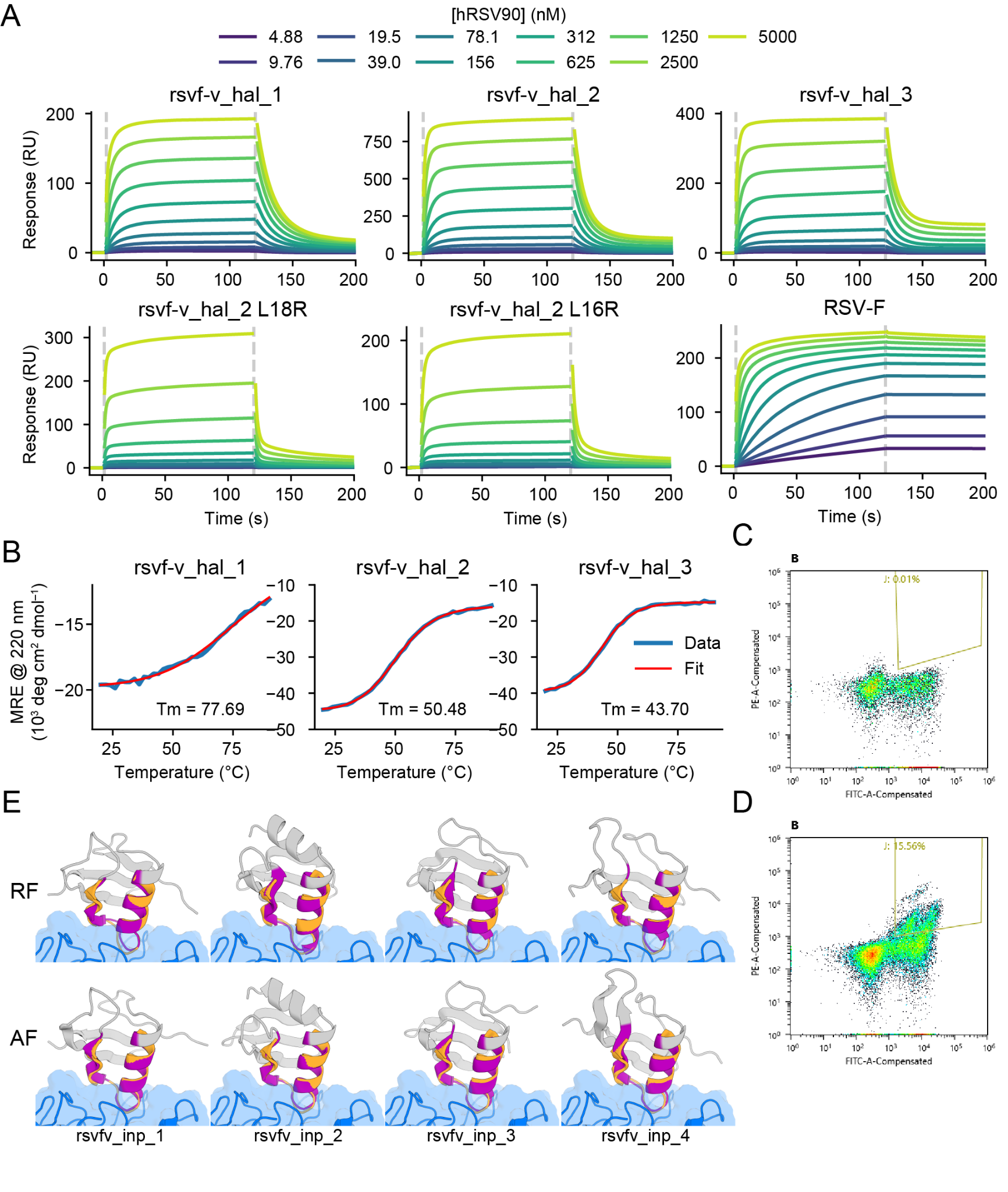


### Figure S11. Experimental characterization of RSV-F site V scaffolds

(A) Binding response (response units) versus time on SPR for RSV-F site V designs, point mutants, and control RSV F protein. Computed K_D_ values are shown in Fig. 2. (B) Mean residue ellipticity at 220 nm versus temperature from CD. Melting points (T_m_) values calculated from a two-state fit are shown in the inset. (C) CD spectra of point mutants with complete loss of activity. (D-E) Compensated PE-A (hRSV90 binding) versus compensated FITC-A (yeast display) for a pool of 56 RSV-F site V inpaints with (D) no target or (E) 100nM binding target. (F) RosettaFold (RF) and Alphafold2 (AF) models of inpainted designs recovered from the sorted cells in (E).


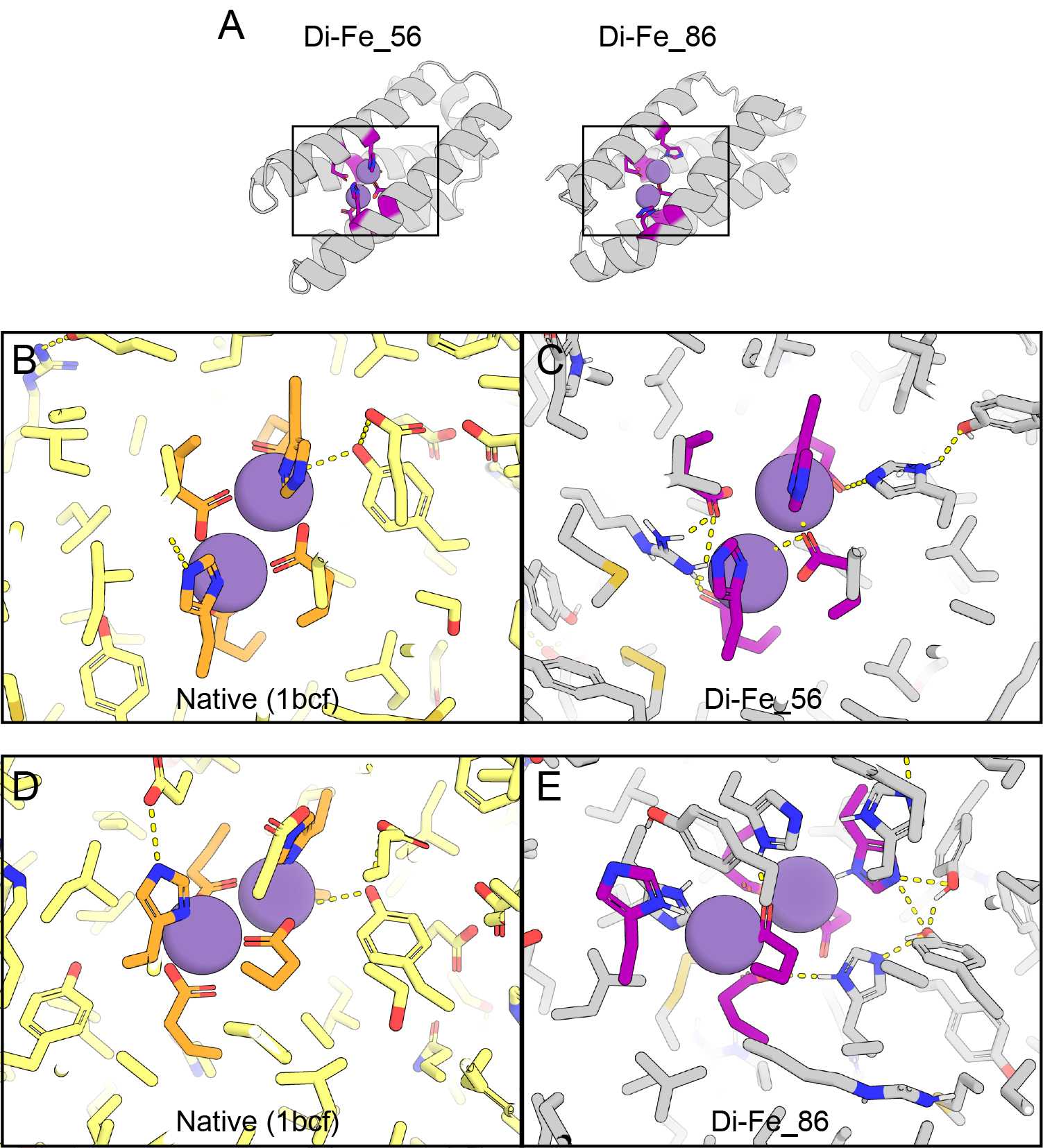


### Figure S12. Di-iron hallucinations containing buried hydrogen-bond networks

(A) Two di-iron hallucinations and close-ups (C, E) of the residues near the metal binding site. Structures are AF predictions after AMBER relax [(*80*)](https://www.zotero.org/google-docs/?seXvzV). The native protein used as a hallucination reference is shown in (B, D) after aligning to the hallucinations on the backbone atoms of the functional residues (orange in native, purple in hallucinations). Metals shown in (C, E) are taken from the native structure after superimposition. Note the presence of hallucinated polar residues (gray histidines and tyrosines) to form hydrogen-bonding networks with the functional histidines and glutamates, which were constrained to their native identities during hallucination.


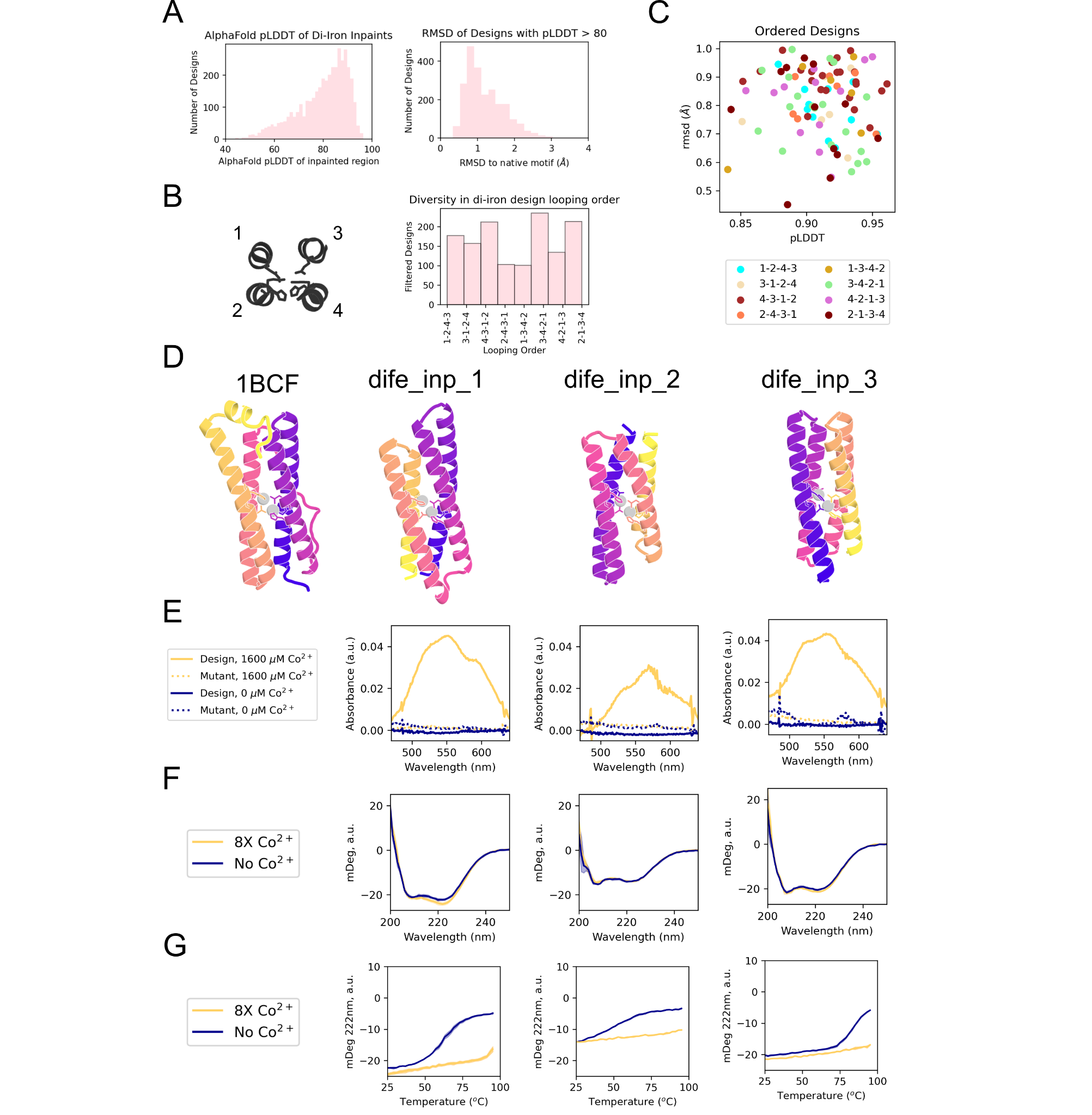


###

### Figure S13. A subset of successful di-iron binding proteins designed with RF*_joint_*

A total of 4000 inpainted designs haboring the bacterioferritin (1BCF) di-iron binding site and encompassing 8 unique looping orders were generated with RF*_joint_*. (A) 57.9% of outputs had AlphaFold pLDDT in the inpainted region > 80 (left), and 43.7% of these designs had a predicted RMSD to the input motif < 1Å (right). (B) All 8 looping orders produced designs with AlphaFold pLDDT > 80 and motif AF-RMSD < 1Å. Looping orders are with respect to residue-indices in the native bacterioferritin protein (left). (C) After filtering and modest sequence optimization with RF***_joint_*** (see supplementary methods), 96 designs were ordered encompassing all 8 looping orders. (D-G) Characterization of three successful designs. (D) AlphaFold predictions of the three designs (right-most three designs), colored to highlight the different looping orders from the native bacterioferritin (left). Iron atoms, aligned to the motif, are depicted in gray for clarity. (pLDDT/Motif AF-RMSD: *dife_inp_1*: 92/0.65Å; *dife_inp_2*: 94/0.64Å; *dife_inp_3*: 90/0.76Å) (E) Designs at 200 μM were incubated with an 8X molar excess of CoCl***_2_***. All three designs show absorbance spectra consistent with Co^2+^ binding in a tetra/penta-coordinate state to the designs (solid yellow lines). Such absorbance was not present in the absence of Co^2+^ (solid blue lines), or with mutant designs where the 6 coordinating residues were mutated to alanine (dashed yellow lines). (F) All designs showed circular-dichroism (CD) spectra consistent with helical proteins. (G) Analysis of protein stability by CD-melts. All three designs were stabilized by binding to metal ions (8X molar excess of Co^2+^). Note that *dife_inp_1* data (E-G) is the same as in Figure 3, reproduced here for convenience.


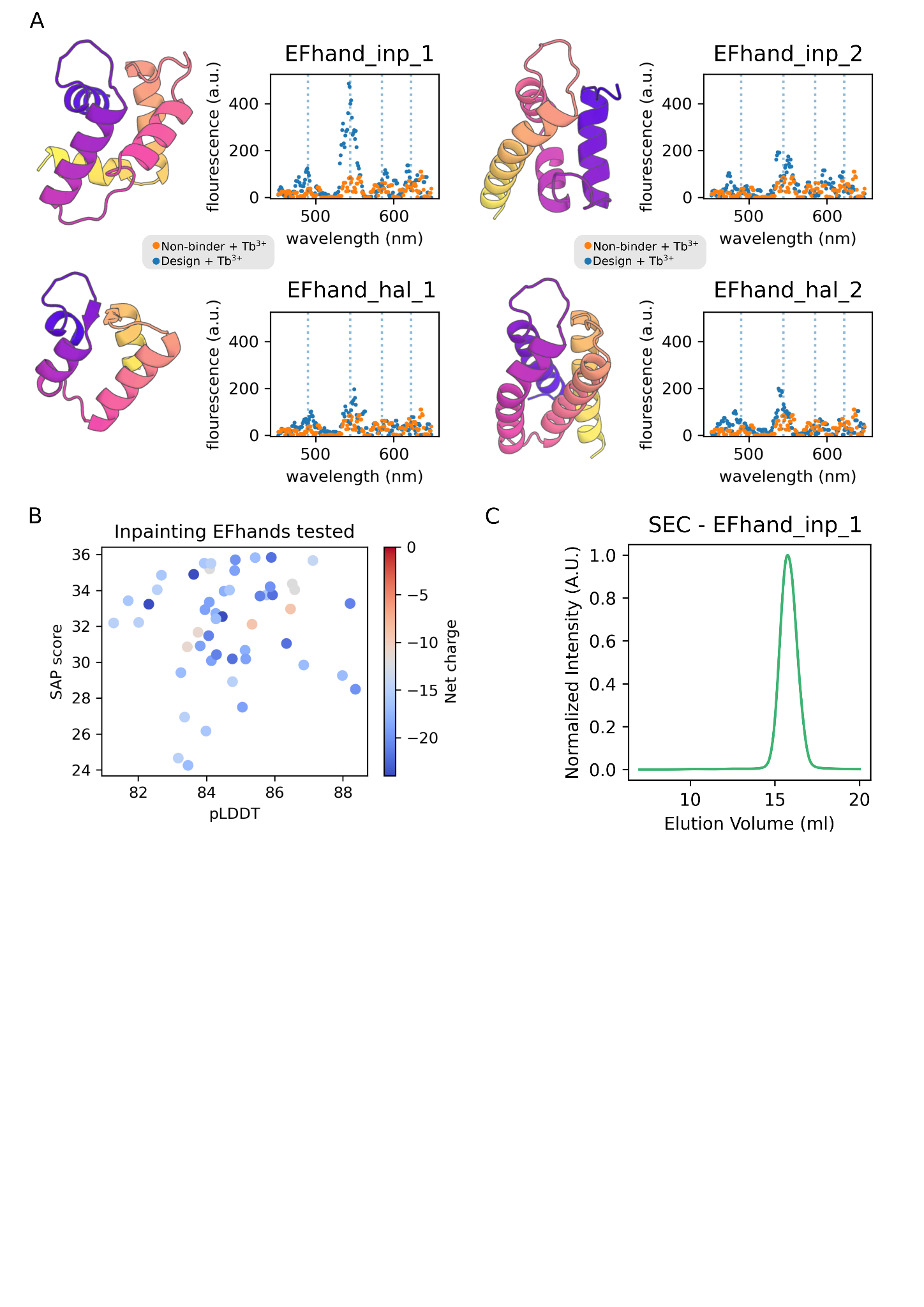


### Figure S14. Characterization of EF-hand designs

Experimental and computational characterization of EF-hand designs tested experimentally. (A) AF2 prediction of inpainted proteins *EFhand_inp_1* and *EFhand_inp_2* (top row) and hallucinated proteins *EFhand_hal_1* and *EFhand_hal_2* (bottom row) next to their terbium fluorescence spectra from a yeast-based initial screen (Materials and Methods). The same negative control spectrum (PDB accession 4DT5, orange) is duplicated across all plots. (B) Computational metrics of inpainted EF-hand designs from RF_joint_ that were tested by yeast display. In addition to standard filters like motif AF-RMSD and AF2 pLDDT, designs were also filtered by their SAP score and net charge. (C) Size exclusion chromatogram at 280 nm absorbance for EFhand_inp_1 suggests the protein occupies a stable monomeric state.


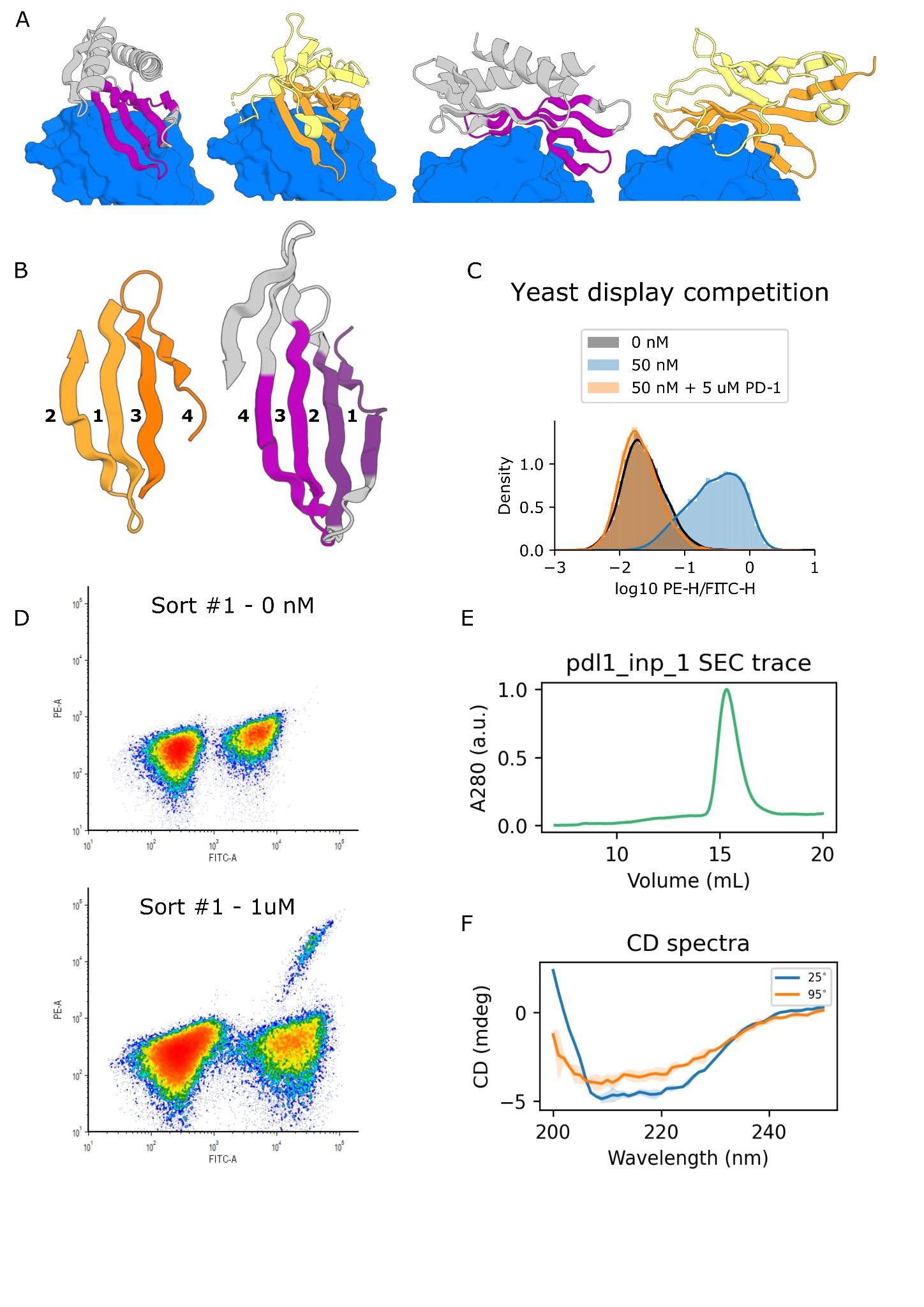


### Figure S15. Experimental characterization of inpainted PD-L1 binder

(A) Crystal structure of HAC PD-1 (binding interface motif in orange) in complex with PD-L1 (blue) and design model of pdl1_inp_1 (motif in purple). The overall fold of the design is quite different from HAC PD-1, as the former contains two buttressing helices against the interfacial sheet instead of the original beta-sandwich. The design also includes an additional beta strand which extends the sheet in its C-terminus. (B) The looping order of the interfacial beta strands in the design (purple / dark purple) has changed dramatically from the HAC PD-1, demonstrating the ease of relooping secondary structure elements while maintaining the desired motif with inpainting. Notably, the order in which the two discontiguous strand-loop-strand submotifs appear in primary structure has switched, as well as the order in which strands 3 and 4 from HAC PD-1, which become strands 1 and 2 in the design, respectively. (C) Binding signal (PE-H) normalized to yeast surface expression (FITC-H) of clonal yeast population displaying pdl1_inp_1 labeled with 0 or 50 nM PD-L1, or 50 nM PD-L1 + 5 μM unlabeled PD-1. Loss of binding upon PD-1 competition suggests that pdl1_inp_1 binds PD-L1 at the native PD-1 binding site. (D) Fluorescence activated cell sorting data from yeast display binding experiments. Titles denote the concentration of a disulfide linked homodimeric PD-L1 target present in the binding reaction. Sort #1 denotes the first pooled sort of 31 designs, Sort #2 denotes the second sort performed with the enriched population of yeast displaying binding activity from Sort #1.


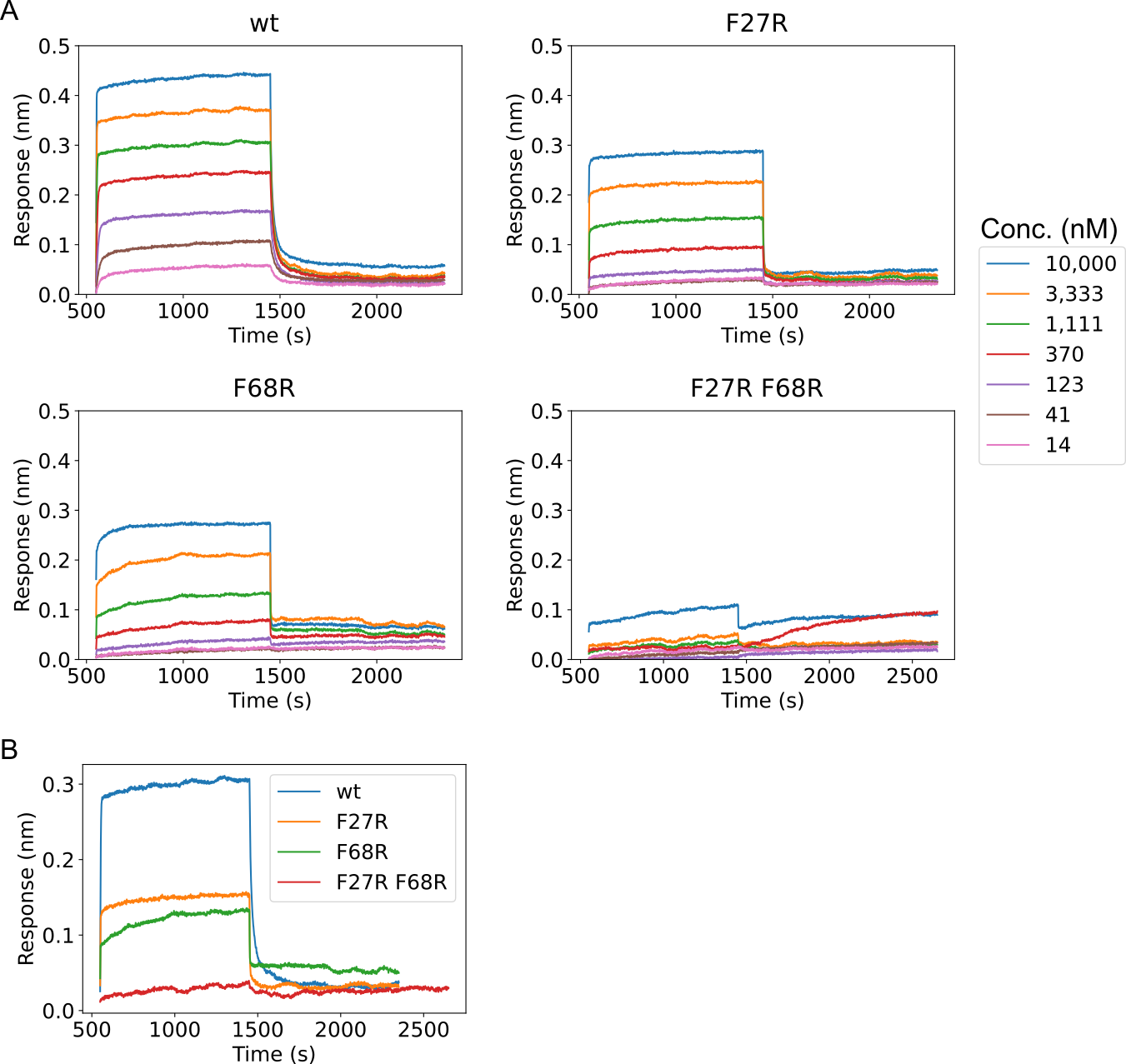


### Figure S16. Experimental characterization of a bivalent TrkA binder

(A) Association and dissociation kinetics of several TrkA binder variants as measured by biolayer interferometry. WT is the designed binder, F27R and F68R are mutants knocking out either one of the designed binding interfaces, and F27R F68R is a double-mutant knocking out both interfaces. (B) Kinetic traces of all four TrkA binder variants compared at the same concentration (1111 nM) show that wt binds the most TrkA, both single site mutants bind similar amounts, and the double mutant binds negligible amounts of TrkA. These data show that either binding site is sufficient to bind TrkA, indicating that we successfully made a bivalent TrkA binder.


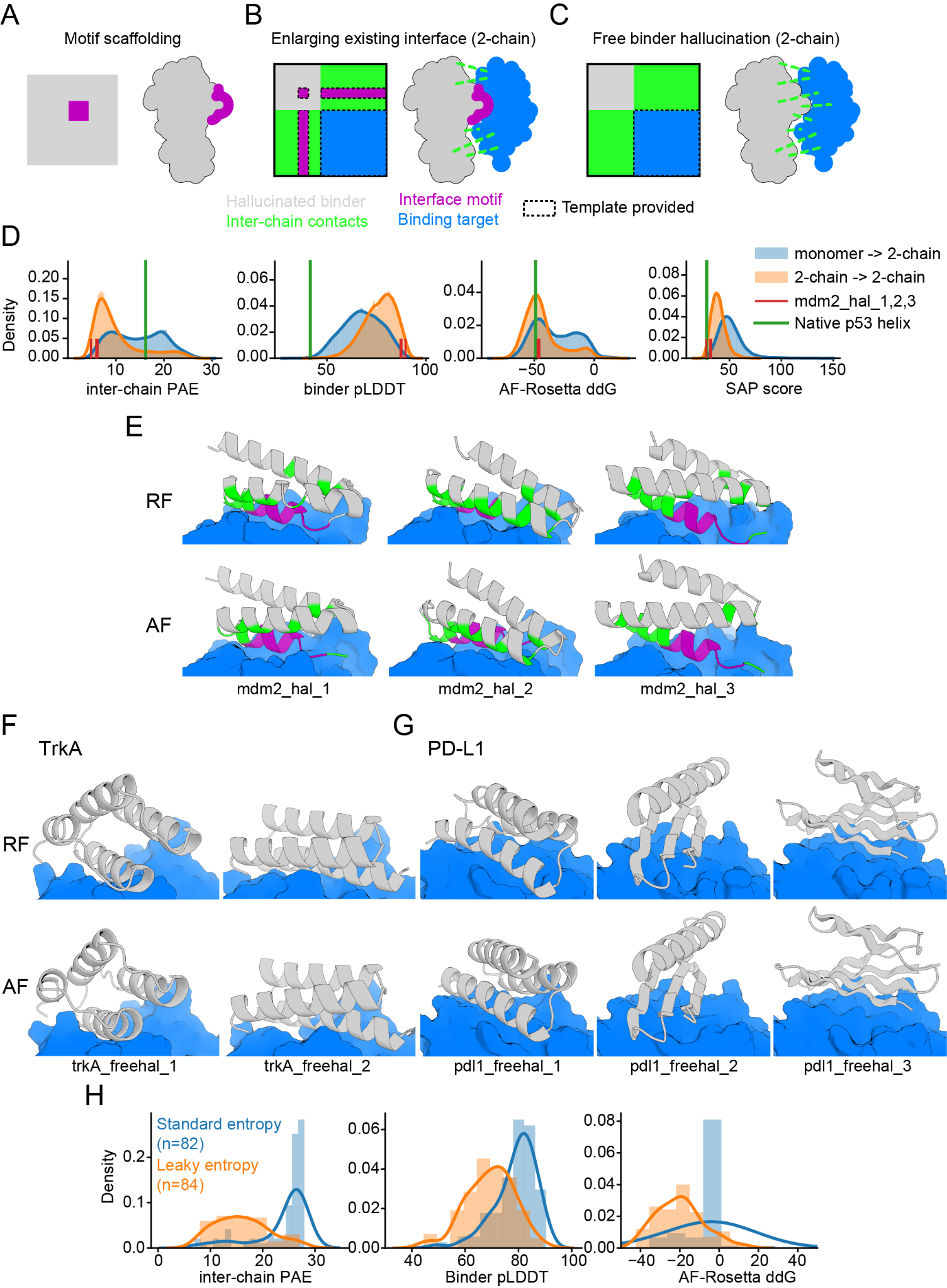


### Figure S17. Multi-chain hallucination for binder design

Schematic of the variations on binder hallucination methods. Hallucinated binder gray, binding partners blue, motifs purple. (A) Motif scaffolding (B) Motif scaffolding while enlarging existing interfaces. (C) Free binder hallucination. (D) Design metrics of 17,450 Mdm2 binder hallucinations. “Monomer -> 2-chain” are designs after one round of two-chain MCMC refinement starting from high-scoring hallucinated monomers (Supplementary Text). “2-chain -> 2-chain” are designs after an additional round of filtering and MCMC refinement. Metrics for the native p53 helix and the 3 highlighted designs are shown in green and red lines, respectively. (E) RF and AF design models of the Mdm2 binder designs shown in Fig. 5G. New binding interactions (hallucinated residues within 5 Å of the target) are in green. (E) Free-hallucinated TrkA and (F) PD-L1 binder designs. (G) Design metrics for free-hallucinated PD-L1 binders using the “leaky” entropy loss (orange), compared to the standard entropy loss (blue) (Supplementary Text).


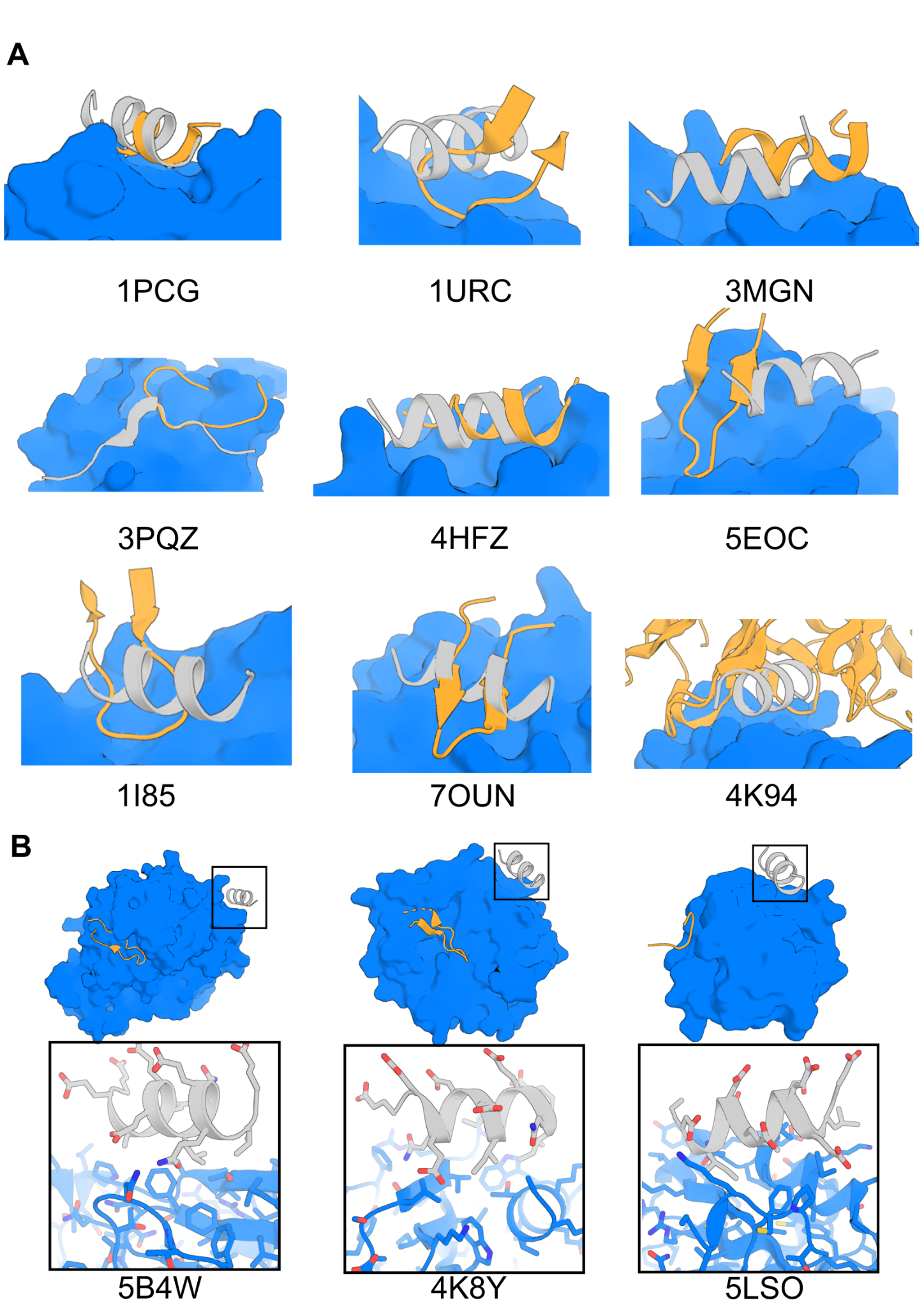


### Figure S18. Free hallucination 12 residue stub placement on native targets

(A) Freely hallucinated 12 residue stubs against native proteins. Gray hallucinated stub; Orange native binder. (B) Hallucinated stubs network docked on alternative hydrophobic grooves to those of native binders. Boxed structures show side chains packing against targets. Structure PDB IDs listed.


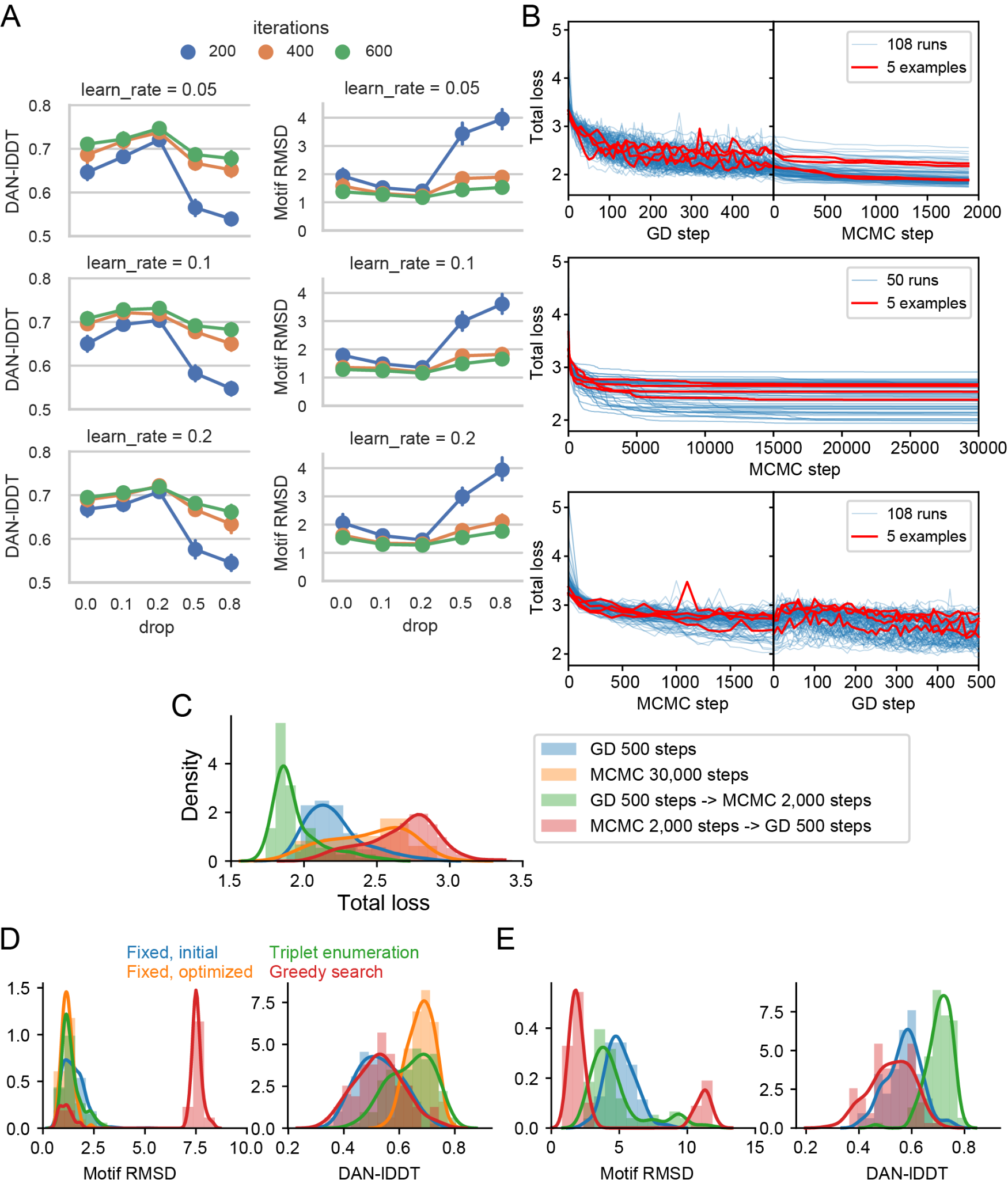


### Figure S19. Hyperparameter tuning and motif-placement methods for hallucination

(A) DeepAccNet-predicted lDDT (DAN-lDDT) and backbone RMSD between hallucination model and reference crystal structure at 22 interface residues for a hyperparameter scan for gradient descent using RosettaFold with a 2-segment motif from HAC PD-1 (residues 63-82 and 119-140). Plotted are mean and 90% confidence interval of 50-100 trajectories per condition. Optimal parameters were dropout = 0.2, learning rate = 0.05. Running 600 iterations gave the best results but 400 steps was comparable and therefore used for most problems. (B) Loss trajectories for gradient descent (GD) followed by MCMC, MCMC only, or MCMC followed by GD. (C) Distributions of final losses for the trajectories shown in (B). (D-E) Motif backbone RMSD and DAN-lDDT for (D) PD-1 mimetics hallucinated with different motif placement methods (Supplementary Text). Motif consists of 2 discontinuous segments as shown in Fig. S1. “Fixed, initial” is an initial run of the fixed motif placement method where contigs are placed anywhere along a given length, and “Fixed, optimized” is a run where gaps between contigs are chosen based on results from the “initial” run. (E) Same methods but with a 2-segment motif from C3d (1GHQ: A104-126,A170-185) and a “fixed, optimized” run was not done.


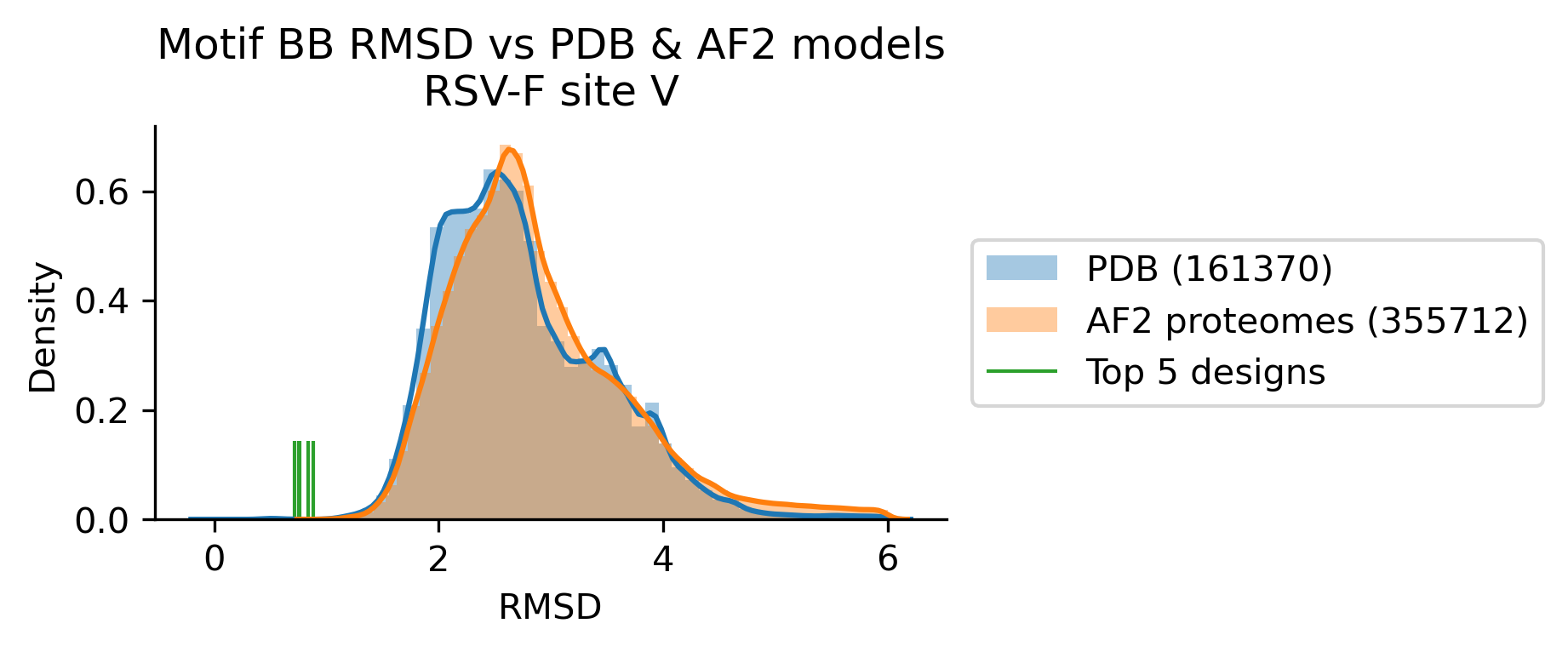


### Figure S20. RMSD of RSV-F motif hits in PDB and AlphaFold proteomes database

Distribution of best RMSD between the RSV-F epitope (PDB 5tpn, chain A residues 163-181) and each structure in the PDB or AlphaFold proteomes database. The motif RMSD of the best 5 hallucinated designs are plotted for comparison. The frequency of finding an RMSD as good as any of these designs or better was 3.9x10^-6^ (see “Native protein scaffold search” in Supplementary Text).


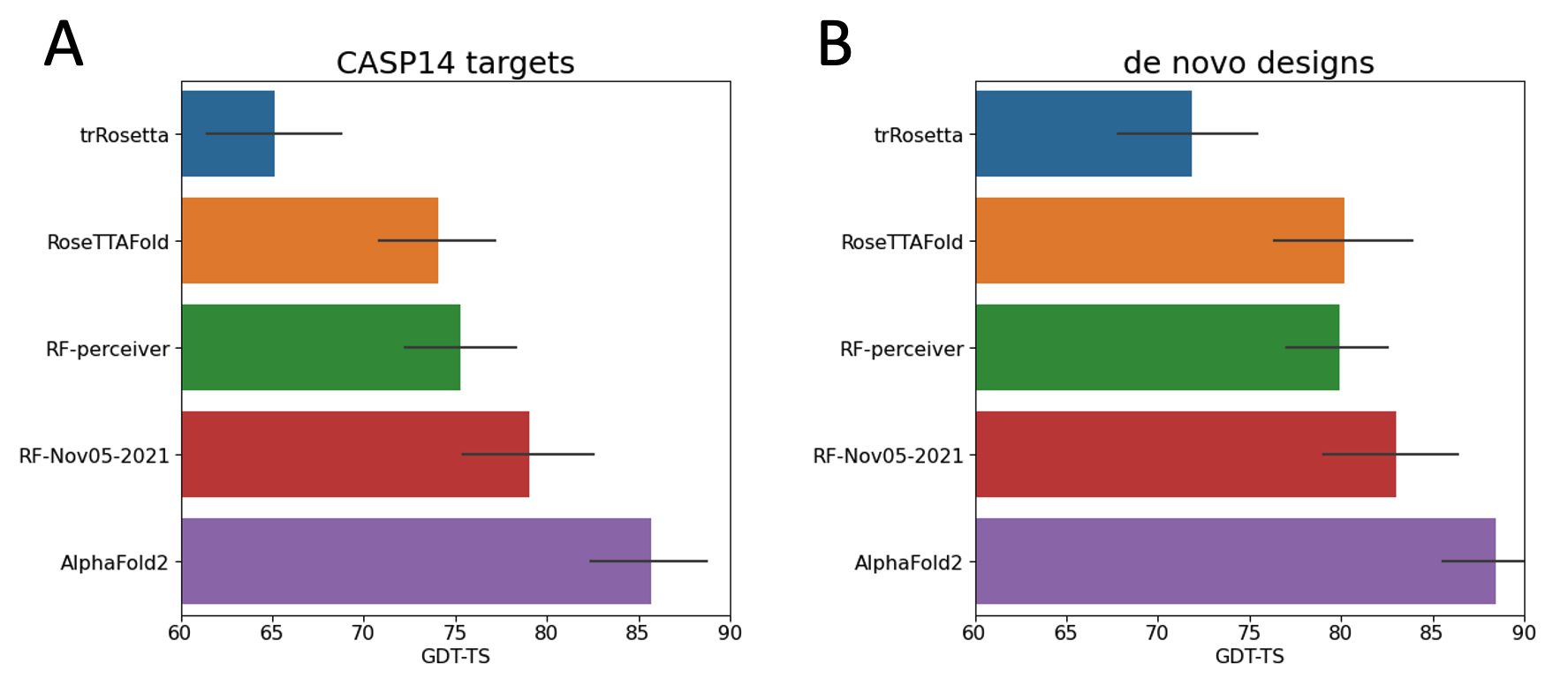


### Figure S21. Structure prediction performance of neural networks used in this study

(A) Average GDT-TS of prediction methods on the CASP14 targets. AlphaFold predictions were made with the same MSA and templates used for the RosettaFold variants. (B) Average GDT-TS of prediction methods on 60 de novo designs not included in the RosettaFold training set. The error bars represent a 95% confidence interval.

###

###

## Supplementary Tables

### Table S1. Natural proteins used for mimetic design

“Motif residues” indicate residues that were constrained to native geometry during hallucination. Sometimes only a subset of the motif residues actually comprise a binding interface or catalytic site; these are denoted “functional residues”.

| **Native protein (Reference)** | **PDB ID** | **Chain** | **Motif residues** | **Functional residues** | **Binding partner(s)** |
| --- | --- | --- | --- | --- | --- |
| HAC PD-1 [(](https://www.zotero.org/google-docs/?D3Jgmv)*15*[)](https://www.zotero.org/google-docs/?D3Jgmv) | 5IUS | A | A63-82, A119-140 | A64, 66, 68, 70, 73-75, 77-78, 81, 85, 89-91, 124, 126, 128, 132, 134, 136, 139 | PD-L1 |
| RSV-F site II [(*81*)](https://www.zotero.org/google-docs/?ACDpgL) | 3IXT | P | P254-277 |  | Antibody |
| RSV-F site V [(*27*)](https://www.zotero.org/google-docs/?BNo7Ia) | 5TPN | A | A163-181 |  | Antibody |
| ACE2 [(*82*)](https://www.zotero.org/google-docs/?InFtA6) | 6VW1 | A | A24-42 |  | SARS-CoV2 receptor binding domain |
| EF-hand [(*83*)](https://www.zotero.org/google-docs/?CQnGMr) | 1PRW | A | A21-31,A56-67 | A21-31,A56-67 | Ca^2+^ |
| Di-Fe [(*29*)](https://www.zotero.org/google-docs/?Tf9bHF) | 1BCF | A | A18-25,A47-54,A94-97,A123-130 | A18, 51, 54, 94, 127, 130 | Fe^2+^ |
| Carbonic anhydrase II [(*84*)](https://www.zotero.org/google-docs/?XcVZkc) | 5YUI | A | A62-65,A93-97,A118-120 | A94,A96,A119,A199 | Zn^2+^ |
| Δ^5^-3-ketosteroid isomerase [(*36*)](https://www.zotero.org/google-docs/?buqG32) | 1QJG | A | A14,A38,A99 | A14,A38,A99 | equilenin |
| p53 N-term helix [(*85*)](https://www.zotero.org/google-docs/?j2gPfM) | 1YCR | B | B17-27 | A19, 23, 26, 27 | Mdm2 |
| TrkA minibinder [(*4*)](https://www.zotero.org/google-docs/?bjW7Ma) | 7N3T | A | A5-18 | A5, 6, 9, 10, 12, 13, 14, 16, 17, 18 | TrkA |

###

### Table S2. RMSDs between native protein, design model, and AlphaFold model

All RMSDs are in angstroms. Columns in red are the metrics reported in the main text and figures. RMSD values in parentheses (for hcA and KSI) are full-atom RMSDs over the catalytic sidechains. KSI designs are generated using AF, and “Design” refers to models generated using the ensembling approach over AF models 1,2,3,5 and “AF” refers to AF model 4 (Materials and methods).

|  | **Overall** | | **Motif** | | |
| --- | --- | --- | --- | --- | --- |
| **Design** | **AF plDDT** | **RMSD, Design to AF** | **RMSD, Design to AF** | **RMSD, Design to native** | **RMSD, AF to native** |
| rsvfv_hal_1 | 82 | 1.37 | 1.06 | 1.31 | 0.7 |
| rsvfv_hal_2 | 88 | 0.75 | 0.34 | 0.67 | 0.64 |
| rsvfv_hal_3 | 86 | 0.85 | 0.24 | 0.65 | 0.65 |
| rsvf-v_854 | 82 | 2.45 | 0.65 | 0.71 | 0.75 |
| rsv_inp_1 | 83 | 0.91 | 0.5 | 0.51 | 0.59 |
| rsv_inp_2 | 83 | 0.76 | 0.57 | 0.6 | 0.81 |
| rsv_inp_3 | 88 | 1.14 | 0.55 | 0.74 | 0.85 |
| rsv_inp_4 | 81 | 1.69 | 0.64 | 0.5 | 0.87 |
| dife_inp_1 | 92 | 0.3 | 0.24 | 0.61 | 0.65 |
| dife_inp_1_mutant | 87 | n/a | n/a | n/a | 0.71 |
| dife_inp_2 | 94 | 0.91 | 0.39 | 0.54 | 0.64 |
| dife_inp_2_mutant | 95 | n/a | n/a | n/a | 0.79 |
| dife_inp_3 | 90 | 0.54 | 0.31 | 0.72 | 0.76 |
| dife_inp_3_mutant | 92 | n/a | n/a | n/a | 0.89 |
| dife_inp_4 | 88 | 1.04 | 0.77 | 0.32 | 0.85 |
| dife_inp_5 | 90 | 0.82 | 0.67 | 0.39 | 0.71 |
| dife_inp_6 | 93 | 0.77 | 0.39 | 0.99 | 0.92 |
| dife_inp_7 | 95 | 0.4 | 0.27 | 0.64 | 0.68 |
| dife_inp_8 | 90 | 0.72 | 0.62 | 0.31 | 0.8 |
| Di-Fe_86 | 84 | 1.97 | 0.89 | 0.4 | 0.9 |
| Di-Fe_56 | 84 | 2.28 | 0.74 | 0.46 | 0.87 |
| EFhand_inp_1 | 87 | 0.86 | 0.82 | 0.29 | 0.69 |
| EFhand_inp_2 | 87.5 | 1.7 | 0.3 | 0.8 | 0.7 |
| EFhand_hal_1 | 82.2 | 1.42 | 0.59 | 0.36 | 0.52 |
| EFhand_hal_2 | 82.8 | 0.76 | 0.47 | 0.55 | 0.73 |
| hcA_1 | 73 | 1.44 | 0.73 (2.23) | 0.75 (1.39) | 1.04 (1.97) |
| hcA_2 | 71 | 1.62 | 0.46 (1.74) | 0.46 (1.36) | 0.62 (2.02) |
| ksi_1 (AF) | 84 | 1.04 | 0.30 (0.30) | 0.30 (1.22) | 0.30 (1.20) |
| ksi_2 (AF) | 72 | 1.06 | 0.16 (0.22) | 0.43 (1.63) | 0.53 (1.65) |
| pdl1_inp_1 | 84 | 0.79 | 0.51 | 1 | 1.1 |
| trkA_56 | 89 | 2.53 | 2.06 | 1.15 | 2.34 |
| mdm2_hal_1 | 88.6 | 1.70 | 1.75 | 0.73 | 1.29 |
| mdm2_hal_2 | 84.1 | 1.95 | 0.83 | 0.59 | 0.63 |
| mdm2_hal_3 | 81.7 | 1.14 | 1.00 | 0.77 | 0.68 |

##

##

### Table S3. Interface metrics of protein-binder designs

AlphaFold inter-PAE, binder pLDDT, AF-Rosetta ddG, and target-aligned binder RMSD (Materials and Methods) for protein-binder designs presented in this paper. Note that designs based off of motifs are listed here and in Table S2, but the free hallucinations are only shown here. pdl1_inp_1 and trkA_56 were not designed using 2-chain hallucination, so there were no RF complex design models to use for target-aligned binder RMSD calculations.

| **Design** | **Inter-PAE** | **Binder pLDDT** | **AF-Rosetta ddG** | **Target-aligned binder RMSD** |
| --- | --- | --- | --- | --- |
| **pdl1_inp_1** | 5.695 | 88.5 | -49.9 | N/A |
| **trkA_56** | 8.428 | 88.4 | -51.8 | N/A |
| **mdm2_hal_1** | 5.904 | 87.6 | -47.2 | 2.93 |
| **mdm2_hal_2** | 4.822 | 89.7 | -45.8 | 3.36 |
| **mdm2_hal_3** | 6.208 | 87.1 | -45.9 | 3.48 |
| **trkA_freehal_1** | 6.40 | 87.4 | -32.5 | 3.87 |
| **trkA_freehal_2** | 4.63 | 92.1 | -35.8 | 1.24 |
| **pdl1_freehal_1** | 5.58 | 84.8 | -38.23 | 3.43 |
| **pdl1_freehal_2** | 9.72 | 82.3 | -26.36 | 1.58 |
| **pdl1_freehal_3** | 8.87 | 81.0 | -37.15 | 1.59 |

###

###

### Table S4. Frequency of suitable native scaffolds

| **Native protein** | **PDB ID** | **Chain** | **Motif residues** | **Scaffolds in the PDB with <1Å motif RMSD** | |
| --- | --- | --- | --- | --- | --- |
|  |  |  |  | **Number** | **Frequency** |
| RSV-F site II | 3IXT | P | P254-277 | 0 | 0 |
| RSV-F site V | 5TPN | A | A163-181 | 1 | 3.76e-05 |
| ACE2 | 6VW1 | A | A24-42 | 1874 | 7.05e-02 |
| EF-hand (double) | 1PRW | A | A21-31,A56-67 | 30 | 1.13e-03 |
| EF-hand (single) | 1PRW | A | A56-67 | 77 | 2.90e-03 |
| Di-iron | 1BCF | A | A18-25,A47-54,A94-97,A123-130 | 3 | 1.13e-04 |
| Carbonic anhydrase II | 5YUI | A | A62-65,A93-97,A118-120 | 1 | 3.76e-05 |
| C3d | 1GHQ | A | A104-126,A170-185 | 2 | 7.52e-05 |
| HAC PD-1 | 5IUS | A | A63-82, A119-140 | 56 | 2.11e-03 |

###

### Table S5. Similarity of designs to native proteins

Designed proteins were compared to protein in the PDB and the Facebook AF2 models database [(*64*)](https://www.zotero.org/google-docs/?ytM2Rm) for structural and sequence similarity with TM-align [(*71*)](https://www.zotero.org/google-docs/?RRmLDH) and blastp [(*86*)](https://www.zotero.org/google-docs/?gv9ZCw), respectively (Materials and Methods). TMalign “% ID” refers to the number of identities over the aligned region divided by the number of aligned residues. BLAST “% ID” refers to the number of identities over the best HSP, normalized to the length of the query sequence (design).

|  | **TMalign to PDB** | | | **TMalign to FB AF2** | | | **BLAST to NR** | | |
| --- | --- | --- | --- | --- | --- | --- | --- | --- | --- |
| **Design** | **Top hit** | **TM score** | **% ID** | **Top hit** | **TM score** | **% ID** | **Top hit** | **E-value** | **% ID** |
| dife_inp_1 | 5vju_A | 0.89 | 9.2 | A0A4S8HXL5 | 0.87 | 8.5 | None | NA | NA |
| dife_inp_1_mutant | 5vju_A | 0.88 | 9.2 | A0A4S8HXL5 | 0.90 | 9.2 | None | NA | NA |
| dife_inp_2 | 7jic_B | 0.84 | 3.5 | A0A328DJV2 | 0.87 | 9.7 | WP_000675503.1 | 2.24E-02 | 23 |
| dife_inp_2_mutant | 7jic_B | 0.82 | 3.5 | A0A1D2MQT9 | 0.89 | 8.8 | None | NA | NA |
| dife_inp_3 | 1yo7_A | 0.85 | 12.3 | A0A3S2NBQ8 | 0.87 | 12.5 | None | NA | NA |
| dife_inp_3_mutant | 4phq_B | 0.83 | 13 | A0A2N1PYQ6 | 0.89 | 11.6 | None | NA | NA |
| dife_inp_4 | 6egc_A | 0.84 | 22.4 | A0A1J0A759 | 0.87 | 5.7 | None | NA | NA |
| dife_inp_5 | 6egc_A | 0.85 | 14.8 | A0A1D8FWU8 | 0.84 | 18.6 | None | NA | NA |
| dife_inp_6 | 5vjs_A | 0.80 | 10.2 | A0A131Z7Y1 | 0.87 | 4.7 | None | NA | NA |
| dife_inp_7 | 5vjs_A | 0.84 | 10.5 | A0A1D1UXZ2 | 0.87 | 9.7 | None | NA | NA |
| dife_inp_8 | 5vju_A | 0.85 | 30.7 | J9JP71 | 0.88 | 9.9 | 2LFD_A | 9.30E-03 | 22 |
| rsv_inp_1 | 5a2q_G | 0.61 | 16.1 | A0A2V8WE05 | 0.63 | 15.7 | 1G2C_A | 5.40E-01 | 40 |
| rsv_inp_2 | 6apd_B | 0.64 | 42.1 | A0A2V9VQU5 | 0.65 | 11.1 | XP_021434148.1 | 6.38E+00 | 30 |
| rsv_inp_3 | 5clr_A | 0.60 | 10.2 | I2B993 | 0.69 | 13.3 | WP_120068072.1 | 1.24E+00 | 29 |
| rsv_inp_4 | 5g4y_A | 0.67 | 11.5 | A0A2N5ZAK5 | 0.66 | 8.1 | WP_159887573.1 | 5.70E+00 | 35 |
| trkA_56 | 2d4c_A | 0.80 | 8.1 | UPI00083C0126 | 0.83 | 8.1 | None | NA | NA |
| rsvfv_hal_1 | 6ntr_D | 0.69 | 10.4 | A0A2H6GLY6 | 0.77 | 10.1 | 3KPE_A | 8.90E-01 | 26 |
| rsvfv_hal_2 | 4dmg_A | 0.71 | 11.9 | A0A290HYD2 | 0.78 | 12.1 | RZV56203.1 | 3.12E+00 | 20 |
| rsvfv_hal_3 | 4auk_A | 0.69 | 11.4 | R7HWW9 | 0.76 | 12.9 | WP_154333053.1 | 4.53E+00 | 31 |
| rsvf-v_854 | 5wb0_F | 0.58 | 21.9 | UPI000B354BFA | 0.67 | 8.6 | 1G2C_A | 5.80E-02 | 27 |
| rsvf-v_870 | 5csl_B | 0.67 | 16.7 | A0A413CFN9 | 0.72 | 11.5 | 1G2C_A | 2.63E+00 | 27 |
| rsvf-v_828 | 6cp8_A | 0.62 | 13.6 | UPI0009045699 | 0.67 | 10.5 | None | NA | NA |
| rsvf-v_903 | 2x32_B | 0.63 | 5 | UPI0011AE9EE2 | 0.74 | 8.2 | 3KPE_A | 1.56E-01 | 32 |
| rsvf-v_1050 | 5wti_Z | 0.59 | 12.7 | A0A524IGV4 | 0.63 | 1.6 | AIZ95772.1 | 3.16E-02 | 32 |
| rsvf-ii_141 | 6ivm_A | 0.68 | 10 | A0A366EM18 | 0.74 | 9.1 | HHG91166.1 | 6.16E+00 | 17 |
| rsvf-ii_171 | 5j0l_E | 0.86 | 12.5 | A0A1Y6CLD7 | 0.89 | 8.7 | AWV19065.1 | 3.23E-01 | 34 |
| rsvf-ii_118 | 2yfa_A | 0.74 | 15.4 | A0A0M0J6I0 | 0.81 | 9 | CCW60917.1 | 1.54E+00 | 27 |
| rsvf-ii_29 | 4jeh_B | 0.78 | 9.1 | R6XLH6 | 0.82 | 4.5 | RKX18559.1 | 2.83E-01 | 17 |
| rsvf-ii_158 | 2j0o_A | 0.86 | 11.1 | A0A354DBJ4 | 0.88 | 8.2 | WP_068486906.1 | 3.04E-01 | 29 |
| ace2_76 | 7jh6_A | 0.81 | 14.6 | A0A073CH21 | 0.86 | 10.4 | QIN87098.1 | 5.91E+00 | 21 |
| ace2_1157 | 2j0o_A | 0.76 | 11.8 | A0A1Y2MHD8 | 0.83 | 3.5 | WP_100023565.1 | 1.17E+00 | 24 |
| ace2_1007 | 5tqy_A | 0.80 | 10.5 | A0A4R7HW16 | 0.86 | 9.6 | None | NA | NA |
| ace2_1846 | 5iig_A | 0.82 | 9.4 | UPI00041B2217 | 0.81 | 10.3 | None | NA | NA |
| ace2_600 | 4q2g_B | 0.72 | 11.1 | R5GU22 | 0.81 | 5 | EPE07190.1 | 2.41E+00 | 27 |
| ace2_109 | 3zcj_B | 0.80 | 9.2 | A0A3N0EL48 | 0.85 | 6.7 | ROL44962.1 | 1.58E-01 | 22 |
| hcA_1 | 2hb0_A | 0.77 | 17.2 | A0A376L8Y0 | 0.75 | 14.8 | WP_107852251.1 | 3.10E-01 | 30 |
| hcA_2 | 6ohh_B | 0.79 | 15.5 | A0A3D3R120 | 0.81 | 10.5 | WP_021068970.1 | 6.97E+00 | 23 |
| ksi_1 (AF) | 5k59_B | 0.73 | 6.2 | M3UPS5 | 0.78 | 4.2 | WP_147602516.1 | 4.22E-01 | 21 |
| ksi_2 (AF) | 1z8k_A | 0.58 | 5.6 | Q66636 | 0.61 | 7.1 | KAF3849996.1 | 2.21E+00 | 25 |
| Di-Fe_86 | 6h2f_H | 0.76 | 5.9 | X0WN74 | 0.85 | 7.8 | None | NA | NA |
| Di-Fe_56 | 6ezv_X | 0.75 | 3.5 | A0A399XE29 | 0.78 | 3.5 | TGO06933.1 | 1.45E+00 | 19 |
| pdl1_inp_1 | 5ldz_F | 0.61 | 8.3 | A0A2N1TGW6 | 0.67 | 6.7 | WP_071803821.1 | 3.28E-02 | 25 |
| EFhand_inp_1 | 4by5_B | 0.75 | 20.7 | A0A2E7SWA3 | 0.77 | 21.4 | XP_020433196.1 | 1.38E-17 | 52 |
| EFhand_inp_2 | 1juo_A | 0.65 | 15.9 | UPI00052857BB | 0.72 | 23.2 | None | 9.52E-05 | 35 |
| EFhand_hal_1 | 2f8p_A | 0.72 | 26.3 | A0A0A1TVZ3 | 0.82 | 16.9 | XP_019463585.1 | 5.04E-02 | 36 |
| EFhand_hal_2 | 6afs_B | 0.67 | 3.1 | UPI0004131E18 | 0.76 | 12.2 | WP_092746209.1 | 6.09E-02 | 23 |
| mdm2_hal_1 | 5h78_A | 0.77 | 17.9 | A0A2T4JJG5 | 0.85 | 15.8 | None | NA | NA |
| mdm2_hal_2 | 1fjg_T | 0.78 | 17.9 | A0A429CN45 | 0.86 | 16.4 | XP_012788760.1 | 2.38E+00 | 27 |
| mdm2_hal_3 | 6w2v_B | 0.86 | 16.7 | A0A1F7QLQ5 | 0.90 | 11.7 | XP_030199201.1 | 7.14E+00 | 27 |
| trkA_freehal_1 | 5wyl_A | 0.75 | 7.9 | UPI0012EDFAF2 | 0.81 | 4.7 | WP_165006269.1 | 3.64E+00 | 25 |
| trkA_freehal_2 | 2oku_A | 0.82 | 6.7 | A0A4R8UL89 | 0.83 | 9.5 | None | NA | NA |
| pdl1_freehal_1 | 3q5d_A | 0.77 | 12.1 | A0A292YNZ8 | 0.80 | 4.9 | WP_132874866.1 | 7.67E+00 | 33 |
| pdl1_freehal_2 | 4jhc_A | 0.78 | 10 | A0A521U212 | 0.86 | 10.3 | MSR05998.1 | 4.94E+00 | 25 |
| pdl1_freehal_3 | 2ygt_A | 0.75 | 3.3 | A0A538M5E7 | 0.79 | 11.9 | PVH99412.1 | 2.20E+00 | 32 |

## Supplementary Algorithms

### Algorithm S1. Joint RosettaFold training Epoch


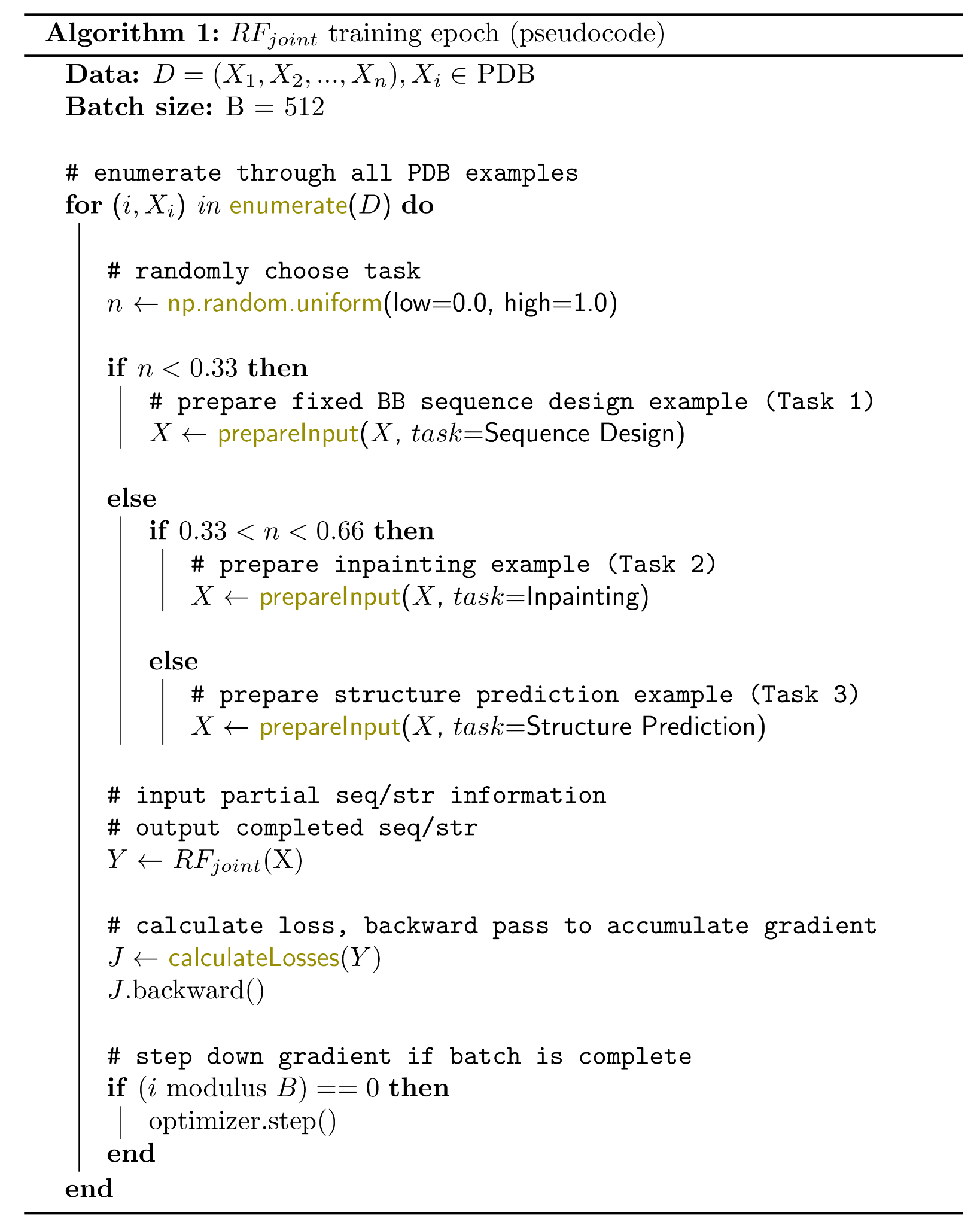


## Supplementary Data

### Data S1. PDB files of all designs in paper

### Data S2. FASTA files of all designed sequences in paper

## References

[1. O. Khersonsky, A. M. Wollacott, L. Jiang, J. Dechancie, J. Betker, J. L. Gallaher, E. A. Althoff, A. Zanghellini, O. Dym, S. Albeck, K. N. Houk, D. S. Tawfik, D. Baker, Kemp elimination catalysts by computational enzyme design. **453** (2008), doi:10.1038/nature06879.](https://www.zotero.org/google-docs/?5JaQi2)

[2. L. Jiang, E. A. Althoff, F. R. Clemente, L. Doyle, D. Röthlisberger, A. Zanghellini, J. L. Gallaher, J. L. Betker, F. Tanaka, C. F. Barbas, D. Hilvert, K. N. Houk, B. L. Stoddard, D. Baker, De Novo Computational Design of Retro-Aldol Enzymes. *Science*. **319**, 1387–1391 (2008).](https://www.zotero.org/google-docs/?5JaQi2)

[3. J. B. Siegel, A. Zanghellini, H. M. Lovick, G. Kiss, A. R. Lambert, J. L. St. Clair, J. Gallaher, D. Hilvert, M. H. Gelb, B. L. Stoddard, K. N. Houk, F. E. Michael, D. Baker, Computational Design of an Enzyme Catalyst for a Stereoselective Bimolecular Diels-Alder Reaction. *Science*. **329** (2010), doi:10.1126/science.1190239.](https://www.zotero.org/google-docs/?5JaQi2)

[4. L. Cao, B. Coventry, I. Goreshnik, B. Huang, J. S. Park, K. M. Jude, I. Marković, R. U. Kadam, K. H. G. Verschueren, K. Verstraete, S. T. R. Walsh, N. Bennett, A. Phal, A. Yang, L. Kozodoy, M. DeWitt, L. Picton, L. Miller, E.-M. Strauch, N. D. DeBouver, A. Pires, A. K. Bera, S. Halabiya, B. Hammerson, W. Yang, S. Bernard, L. Stewart, I. A. Wilson, H. Ruohola-Baker, J. Schlessinger, S. Lee, S. N. Savvides, K. C. Garcia, D. Baker, Design of protein binding proteins from target structure alone. *Nature* (2022), doi:10.1038/s41586-022-04654-9.](https://www.zotero.org/google-docs/?5JaQi2)

[5. A. A. Chevalier, D. Silva, G. J. Rocklin, R. Derrick, R. Vergara, P. Murapa, S. M. Bernard, L. Zhang, G. Yao, C. D. Bahl, S. Miyashita, I. Goreshnik, T. James, M. Bryan, D. A. Fernández-velasco, L. Stewart, M. Dong, X. Huang, Massively parallel de novo protein design for targeted therapeutics. *Nat. Publ. Group* (2017), doi:10.1038/nature23912.](https://www.zotero.org/google-docs/?5JaQi2)

[6. E. Procko, G. Y. Berguig, B. W. Shen, Y. Song, S. Frayo, A. J. Convertine, D. Margineantu, G. Booth, B. E. Correia, Y. Cheng, W. R. Schief, D. M. Hockenbery, O. W. Press, B. L. Stoddard, P. S. Stayton, D. Baker, A Computationally Designed Inhibitor of an Epstein-Barr Viral Bcl-2 Protein Induces Apoptosis in Infected Cells. *Cell*. **157**, 1644–1656 (2014).](https://www.zotero.org/google-docs/?5JaQi2)

[7. B. E. Correia, J. T. Bates, R. J. Loomis, G. Baneyx, C. Carrico, J. G. Jardine, P. Rupert, C. Correnti, O. Kalyuzhniy, V. Vittal, M. J. Connell, E. Stevens, A. Schroeter, M. Chen, S. MacPherson, A. M. Serra, Y. Adachi, M. A. Holmes, Y. Li, R. E. Klevit, B. S. Graham, R. T. Wyatt, D. Baker, R. K. Strong, J. E. Crowe, P. R. Johnson, W. R. Schief, Proof of principle for epitope-focused vaccine design. *Nature*. **507**, 201–206 (2014).](https://www.zotero.org/google-docs/?5JaQi2)

[8. D.-A. Silva, S. Yu, U. Y. Ulge, J. B. Spangler, K. M. Jude, C. Labão-Almeida, L. R. Ali, A. Quijano-Rubio, M. Ruterbusch, I. Leung, T. Biary, S. J. Crowley, E. Marcos, C. D. Walkey, B. D. Weitzner, F. Pardo-Avila, J. Castellanos, L. Carter, L. Stewart, S. R. Riddell, M. Pepper, G. J. L. Bernardes, M. Dougan, K. C. Garcia, D. Baker, De novo design of potent and selective mimics of IL-2 and IL-15. *Nature*. **565**, 186–191 (2019).](https://www.zotero.org/google-docs/?5JaQi2)

[9. F. Sesterhenn, C. Yang, J. Bonet, J. T. Cramer, X. Wen, Y. Wang, C.-I. Chiang, L. A. Abriata, I. Kucharska, G. Castoro, S. S. Vollers, M. Galloux, E. Dheilly, S. Rosset, P. Corthésy, S. Georgeon, M. Villard, C.-A. Richard, D. Descamps, T. Delgado, E. Oricchio, M.-A. Rameix-Welti, V. Más, S. Ervin, J.-F. Eléouët, S. Riffault, J. T. Bates, J.-P. Julien, Y. Li, T. Jardetzky, T. Krey, B. E. Correia, De novo protein design enables the precise induction of RSV-neutralizing antibodies. *Science*. **368** (2020), doi:10.1126/science.aay5051.](https://www.zotero.org/google-docs/?5JaQi2)

[10. C. Yang, F. Sesterhenn, J. Bonet, E. A. van Aalen, L. Scheller, L. A. Abriata, J. T. Cramer, X. Wen, S. Rosset, S. Georgeon, T. Jardetzky, T. Krey, M. Fussenegger, M. Merkx, B. E. Correia, Bottom-up de novo design of functional proteins with complex structural features. *Nat. Chem. Biol.*, 1–9 (2021).](https://www.zotero.org/google-docs/?5JaQi2)

[11. J. Yang, I. Anishchenko, H. Park, Z. Peng, S. Ovchinnikov, D. Baker, Improved protein structure prediction using predicted interresidue orientations. *Proc. Natl. Acad. Sci.* (2020), doi:10.1073/pnas.1914677117.](https://www.zotero.org/google-docs/?5JaQi2)

[12. I. Anishchenko, S. J. Pellock, T. M. Chidyausiku, T. A. Ramelot, S. Ovchinnikov, J. Hao, K. Bafna, C. Norn, A. Kang, A. K. Bera, F. DiMaio, L. Carter, C. M. Chow, G. T. Montelione, D. Baker, De novo protein design by deep network hallucination. *Nature*. **600**, 547–552 (2021).](https://www.zotero.org/google-docs/?5JaQi2)

[13. C. Norn, B. I. M. Wicky, D. Juergens, S. Liu, D. Kim, D. Tischer, B. Koepnick, I. Anishchenko, F. Players, D. Baker, S. Ovchinnikov, Protein sequence design by conformational landscape optimization. *Proc. Natl. Acad. Sci.* **118** (2021), doi:10.1073/pnas.2017228118.](https://www.zotero.org/google-docs/?5JaQi2)

[14. D. Tischer, S. Lisanza, J. Wang, R. Dong, I. Anishchenko, L. F. Milles, S. Ovchinnikov, D. Baker, *bioRxiv*, in press, doi:10.1101/2020.11.29.402743.](https://www.zotero.org/google-docs/?5JaQi2)

[15. R. Pascolutti, X. Sun, J. Kao, R. L. Maute, A. M. Ring, G. R. Bowman, A. C. Kruse, Structure and Dynamics of PD-L1 and an Ultra-High-Affinity PD-1 Receptor Mutant. *Structure*. **24**, 1719–1728 (2016).](https://www.zotero.org/google-docs/?5JaQi2)

[16. M. Baek, F. DiMaio, I. Anishchenko, J. Dauparas, S. Ovchinnikov, G. R. Lee, J. Wang, Q. Cong, L. N. Kinch, R. D. Schaeffer, C. Millán, H. Park, C. Adams, C. R. Glassman, A. DeGiovanni, J. H. Pereira, A. V. Rodrigues, A. A. van Dijk, A. C. Ebrecht, D. J. Opperman, T. Sagmeister, C. Buhlheller, T. Pavkov-Keller, M. K. Rathinaswamy, U. Dalwadi, C. K. Yip, J. E. Burke, K. C. Garcia, N. V. Grishin, P. D. Adams, R. J. Read, D. Baker, Accurate prediction of protein structures and interactions using a three-track neural network. *Science* (2021), doi:10.1126/science.abj8754.](https://www.zotero.org/google-docs/?5JaQi2)

[17. J. Devlin, M.-W. Chang, K. Lee, K. Toutanova, BERT: Pre-training of Deep Bidirectional Transformers for Language Understanding. *ArXiv181004805 Cs* (2019) (available at http://arxiv.org/abs/1810.04805).](https://www.zotero.org/google-docs/?5JaQi2)

[18. R. A. Yeh, C. Chen, T. Y. Lim, A. G. Schwing, M. Hasegawa-Johnson, M. N. Do, Semantic Image Inpainting with Deep Generative Models. *ArXiv160707539 Cs* (2017) (available at http://arxiv.org/abs/1607.07539).](https://www.zotero.org/google-docs/?5JaQi2)

[19. Z. Li, S. P. Nguyen, D. Xu, Y. Shang, in *2017 IEEE 29th International Conference on Tools with Artificial Intelligence (ICTAI)* (2017), pp. 1085–1091.](https://www.zotero.org/google-docs/?5JaQi2)

[20. N. Anand, P. Huang, in *Advances in Neural Information Processing Systems 31*, S. Bengio, H. Wallach, H. Larochelle, K. Grauman, N. Cesa-Bianchi, R. Garnett, Eds. (Curran Associates, Inc., 2018; http://papers.nips.cc/paper/7978-generative-modeling-for-protein-structures.pdf), pp. 7494–7505.](https://www.zotero.org/google-docs/?5JaQi2)

[21. J. Jumper, R. Evans, A. Pritzel, T. Green, M. Figurnov, O. Ronneberger, K. Tunyasuvunakool, R. Bates, A. Žídek, A. Potapenko, A. Bridgland, C. Meyer, S. A. A. Kohl, A. J. Ballard, A. Cowie, B. Romera-Paredes, S. Nikolov, R. Jain, J. Adler, T. Back, S. Petersen, D. Reiman, E. Clancy, M. Zielinski, M. Steinegger, M. Pacholska, T. Berghammer, S. Bodenstein, D. Silver, O. Vinyals, A. W. Senior, K. Kavukcuoglu, P. Kohli, D. Hassabis, Highly accurate protein structure prediction with AlphaFold. *Nature*. **596**, 583–589 (2021).](https://www.zotero.org/google-docs/?5JaQi2)

[22. R. Chowdhury, N. Bouatta, S. Biswas, C. Rochereau, G. M. Church, P. K. Sorger, M. AlQuraishi, Single-sequence protein structure prediction using language models from deep learning, 22.](https://www.zotero.org/google-docs/?5JaQi2)

[23. K. T. Simons, R. Bonneau, I. Ruczinski, D. Baker, Ab initio protein structure prediction of CASP III targets using ROSETTA. *Proteins Struct. Funct. Bioinforma.* **37**, 171–176 (1999).](https://www.zotero.org/google-docs/?5JaQi2)

[24. T.-E. Kim, K. Tsuboyama, S. Houliston, C. M. Martell, C. M. Phoumyvong, H. K. Haddox, C. H. Arrowsmith, G. J. Rocklin, Dissecting the stability determinants of a challenging de novo protein fold using massively parallel design and experimentation (2021), p. 2021.12.17.472837, , doi:10.1101/2021.12.17.472837.](https://www.zotero.org/google-docs/?5JaQi2)

[25. M. A. Pak, K. A. Markhieva, M. S. Novikova, D. S. Petrov, I. S. Vorobyev, E. S. Maksimova, F. A. Kondrashov, D. N. Ivankov, Using AlphaFold to predict the impact of single mutations on protein stability and function (2021), p. 2021.09.19.460937, , doi:10.1101/2021.09.19.460937.](https://www.zotero.org/google-docs/?5JaQi2)

[26. G. R. Buel, K. J. Walters, Can AlphaFold2 predict the impact of missense mutations on structure? *Nat. Struct. Mol. Biol.* **29**, 1–2 (2022).](https://www.zotero.org/google-docs/?5JaQi2)

[27. J. J. Mousa, N. Kose, P. Matta, P. Gilchuk, J. E. Crowe, A novel pre-fusion conformation-specific neutralizing epitope on the respiratory syncytial virus fusion protein. *Nat. Microbiol.* **2**, 1–8 (2017).](https://www.zotero.org/google-docs/?5JaQi2)

[28. T. W. Linsky, R. Vergara, N. Codina, J. W. Nelson, M. J. Walker, W. Su, C. O. Barnes, T.-Y. Hsiang, K. Esser-Nobis, K. Yu, Z. B. Reneer, Y. J. Hou, T. Priya, M. Mitsumoto, A. Pong, U. Y. Lau, M. L. Mason, J. Chen, A. Chen, T. Berrocal, H. Peng, N. S. Clairmont, J. Castellanos, Y.-R. Lin, A. Josephson-Day, R. S. Baric, D. H. Fuller, C. D. Walkey, T. M. Ross, R. Swanson, P. J. Bjorkman, M. Gale, L. M. Blancas-Mejia, H.-L. Yen, D.-A. Silva, De novo design of potent and resilient hACE2 decoys to neutralize SARS-CoV-2. *Science* (2020), doi:10.1126/science.abe0075.](https://www.zotero.org/google-docs/?5JaQi2)

[29. F. Frolow, A. J. Kalb (Gilboa), J. Yariv, Structure of a unique twofold symmetric haem-binding site. *Nat. Struct. Biol.* **1**, 453–460 (1994).](https://www.zotero.org/google-docs/?5JaQi2)

[30. A. Lombardi, F. Pirro, O. Maglio, M. Chino, W. F. DeGrado, De Novo Design of Four-Helix Bundle Metalloproteins: One Scaffold, Diverse Reactivities. *Acc. Chem. Res.* **52**, 1148–1159 (2019).](https://www.zotero.org/google-docs/?5JaQi2)

[31. J. R. Calhoun, F. Nastri, O. Maglio, V. Pavone, A. Lombardi, W. F. DeGrado, Artificial diiron proteins: From structure to function. *Pept. Sci.* **80**, 264–278 (2005).](https://www.zotero.org/google-docs/?5JaQi2)

[32. A. M. Keech, N. E. L. Brun, M. T. Wilson, S. C. Andrews, G. R. Moore, A. J. Thomson, Spectroscopic Studies of Cobalt(II) Binding to Escherichia coli Bacterioferritin*. *J. Biol. Chem.* **272**, 422–429 (1997).](https://www.zotero.org/google-docs/?5JaQi2)

[33. E. N. G. Marsh, W. F. DeGrado, Noncovalent self-assembly of a heterotetrameric diiron protein. *Proc. Natl. Acad. Sci.* **99**, 5150–5154 (2002).](https://www.zotero.org/google-docs/?5JaQi2)

[34. M. Yáñez, J. Gil-Longo, M. Campos-Toimil, in *Calcium Signaling*, Md. S. Islam, Ed. (Springer Netherlands, Dordrecht, 2012; https://doi.org/10.1007/978-94-007-2888-2_19), *Advances in Experimental Medicine and Biology*, pp. 461–482.](https://www.zotero.org/google-docs/?5JaQi2)

[35. S. J. Caldwell, I. C. Haydon, N. Piperidou, P.-S. Huang, M. J. Bick, H. S. Sjöström, D. Hilvert, D. Baker, C. Zeymer, Tight and specific lanthanide binding in a de novo TIM barrel with a large internal cavity designed by symmetric domain fusion. *Proc. Natl. Acad. Sci.* **117**, 30362–30369 (2020).](https://www.zotero.org/google-docs/?5JaQi2)

[36. H.-S. Cho, N.-C. Ha, G. Choi, H.-J. Kim, D. Lee, K. S. Oh, K. S. Kim, W. Lee, K. Y. Choi, B.-H. Oh, Crystal Structure of Δ5-3-Ketosteroid Isomerase from Pseudomonas testosteroni in Complex with Equilenin Settles the Correct Hydrogen Bonding Scheme for Transition State Stabilization*. *J. Biol. Chem.* **274**, 32863–32868 (1999).](https://www.zotero.org/google-docs/?5JaQi2)

[37. R. L. Maute, S. R. Gordon, A. T. Mayer, M. N. McCracken, A. Natarajan, N. G. Ring, R. Kimura, J. M. Tsai, A. Manglik, A. C. Kruse, S. S. Gambhir, I. L. Weissman, A. M. Ring, Engineering high-affinity PD-1 variants for optimized immunotherapy and immuno-PET imaging. *Proc. Natl. Acad. Sci.* **112**, E6506–E6514 (2015).](https://www.zotero.org/google-docs/?5JaQi2)

[38. H. M. Berman, J. Westbrook, Z. Feng, G. Gilliland, T. N. Bhat, H. Weissig, I. N. Shindyalov, P. E. Bourne, The Protein Data Bank. *Nucleic Acids Res.* **28**, 235–242 (2000).](https://www.zotero.org/google-docs/?5JaQi2)

[39. C. Wiesmann, M. H. Ultsch, S. H. Bass, A. M. de Vos, Crystal structure of nerve growth factor in complex with the ligand-binding domain of the TrkA receptor. *Nature*. **401**, 184–188 (1999).](https://www.zotero.org/google-docs/?5JaQi2)

[40. I. R. Humphreys, J. Pei, M. Baek, A. Krishnakumar, I. Anishchenko, S. Ovchinnikov, J. Zhang, T. J. Ness, S. Banjade, S. R. Bagde, V. G. Stancheva, X.-H. Li, K. Liu, Z. Zheng, D. J. Barrero, U. Roy, J. Kuper, I. S. Fernández, B. Szakal, D. Branzei, J. Rizo, C. Kisker, E. C. Greene, S. Biggins, S. Keeney, E. A. Miller, J. C. Fromme, T. L. Hendrickson, Q. Cong, D. Baker, Computed structures of core eukaryotic protein complexes. *Science*. **0**, eabm4805.](https://www.zotero.org/google-docs/?5JaQi2)

[41. K. Tunyasuvunakool, J. Adler, Z. Wu, T. Green, M. Zielinski, A. Žídek, A. Bridgland, A. Cowie, C. Meyer, A. Laydon, S. Velankar, G. J. Kleywegt, A. Bateman, R. Evans, A. Pritzel, M. Figurnov, O. Ronneberger, R. Bates, S. A. A. Kohl, A. Potapenko, A. J. Ballard, B. Romera-Paredes, S. Nikolov, R. Jain, E. Clancy, D. Reiman, S. Petersen, A. W. Senior, K. Kavukcuoglu, E. Birney, P. Kohli, J. Jumper, D. Hassabis, Highly accurate protein structure prediction for the human proteome. *Nature* (2021), doi:10.1038/s41586-021-03828-1.](https://www.zotero.org/google-docs/?5JaQi2)

[42. J. Ingraham, V. K. Garg, R. Barzilay, T. Jaakkola, Generative models for graph-based protein design, 10 (2019).](https://www.zotero.org/google-docs/?5JaQi2)

[43. A. Strokach, D. Becerra, C. Corbi-Verge, A. Perez-Riba, P. M. Kim, Fast and Flexible Protein Design Using Deep Graph Neural Networks. *Cell Syst.* **11**, 402-411.e4 (2020).](https://www.zotero.org/google-docs/?5JaQi2)

[44. S. Biswas, G. Khimulya, E. C. Alley, K. M. Esvelt, G. M. Church, Low- N protein engineering with data-efficient deep learning. *Nat. Methods*. **18**, 389–396 (2021).](https://www.zotero.org/google-docs/?5JaQi2)

[45. D. Repecka, V. Jauniskis, L. Karpus, E. Rembeza, J. Zrimec, S. Poviloniene, I. Rokaitis, A. Laurynenas, W. Abuajwa, O. Savolainen, R. Meskys, M. K. M. Engqvist, A. Zelezniak, Expanding functional protein sequence space using generative adversarial networks. *bioRxiv*, 789719 (2019).](https://www.zotero.org/google-docs/?5JaQi2)

[46. J.-E. Shin, A. J. Riesselman, A. W. Kollasch, C. McMahon, E. Simon, C. Sander, A. Manglik, A. C. Kruse, D. S. Marks, Protein design and variant prediction using autoregressive generative models. *Nat. Commun.* **12**, 1–11 (2021).](https://www.zotero.org/google-docs/?5JaQi2)

[47. Z. Wu, K. E. Johnston, F. H. Arnold, K. K. Yang, Protein sequence design with deep generative models. *Curr. Opin. Chem. Biol.* **65**, 18–27 (2021).](https://www.zotero.org/google-docs/?5JaQi2)

[48. N. Anand-Achim, R. R. Eguchi, A. Derry, R. B. Altman, P.-S. Huang, “Protein sequence design with a learned potential” (preprint, Bioinformatics, 2020), , doi:10.1101/2020.01.06.895466.](https://www.zotero.org/google-docs/?5JaQi2)

[49. A. Madani, B. Krause, E. R. Greene, S. Subramanian, B. P. Mohr, J. M. Holton, J. L. Olmos, C. Xiong, Z. Z. Sun, R. Socher, J. S. Fraser, N. Naik, *bioRxiv*, in press, doi:10.1101/2021.07.18.452833.](https://www.zotero.org/google-docs/?5JaQi2)

[50. S. Ovchinnikov, P.-S. Huang, Structure-based protein design with deep learning. *Curr. Opin. Chem. Biol.* **65**, 136–144 (2021).](https://www.zotero.org/google-docs/?5JaQi2)

[51. N. Anand, R. Eguchi, P.-S. Huang, Fully differentiable full-atom protein backbone generation (2019) (available at https://openreview.net/forum?id=SJxnVL8YOV).](https://www.zotero.org/google-docs/?5JaQi2)

[52. R. R. Eguchi, N. Anand, C. A. Choe, P.-S. Huang, *bioRxiv*, in press, doi:10.1101/2020.08.07.242347.](https://www.zotero.org/google-docs/?5JaQi2)

[53. Z. Lin, T. Sercu, Y. LeCun, A. Rives, Deep generative models create new and diverse protein structures, 17.](https://www.zotero.org/google-docs/?5JaQi2)

[54. M. Jendrusch, J. O. Korbel, S. K. Sadiq, *bioRxiv*, in press, doi:10.1101/2021.10.11.463937.](https://www.zotero.org/google-docs/?5JaQi2)

[55. L. Moffat, J. G. Greener, D. T. Jones, *bioRxiv*, in press, doi:10.1101/2021.08.24.457549.](https://www.zotero.org/google-docs/?5JaQi2)

[56. L. Moffat, S. M. Kandathil, D. T. Jones, Design in the DARK: Learning Deep Generative Models for De Novo Protein Design (2022), p. 2022.01.27.478087, , doi:10.1101/2022.01.27.478087.](https://www.zotero.org/google-docs/?5JaQi2)

[57. L. Li, Y. Liu, J. Tao, M. Zhang, H. Pan, X. Xu, R. Tang, Surface Modification of Hydroxyapatite Nanocrystallite by a Small Amount of Terbium Provides a Biocompatible Fluorescent Probe. *J. Phys. Chem. C*. **112**, 12219–12224 (2008).](https://www.zotero.org/google-docs/?5JaQi2)

[58. J. Wang, S. Lisanza, D. Juergens, D. Tischer, J. Watson, RFDesign: Protein hallucination and inpainting with RosettaFold, version 1, Zenodo (2022); https://doi.org/10.5281/zenodo.6808038.](https://www.zotero.org/google-docs/?5JaQi2)

[59. E. Jang, S. Gu, B. Poole, Categorical Reparameterization with Gumbel-Softmax. *ArXiv161101144 Cs Stat* (2017) (available at http://arxiv.org/abs/1611.01144).](https://www.zotero.org/google-docs/?5JaQi2)

[60. N. Bogard, J. Linder, A. B. Rosenberg, G. Seelig, A Deep Neural Network for Predicting and Engineering Alternative Polyadenylation. *Cell*. **178**, 91-106.e23 (2019).](https://www.zotero.org/google-docs/?5JaQi2)

[61. J. Linder, G. Seelig, Fast differentiable DNA and protein sequence optimization for molecular design. *ArXiv200511275 Cs Stat* (2020) (available at http://arxiv.org/abs/2005.11275).](https://www.zotero.org/google-docs/?5JaQi2)

[62. D. P. Kingma, J. Ba, Adam: A Method for Stochastic Optimization. *ArXiv14126980 Cs* (2017) (available at http://arxiv.org/abs/1412.6980).](https://www.zotero.org/google-docs/?5JaQi2)

[63. R. M. Rao, J. Liu, R. Verkuil, J. Meier, J. Canny, P. Abbeel, T. Sercu, A. Rives, *bioRxiv*, in press, doi:10.1101/2021.02.12.430858.](https://www.zotero.org/google-docs/?5JaQi2)

[64. C. Hsu, R. Verkuil, J. Liu, Z. Lin, B. Hie, T. Sercu, A. Lerer, A. Rives, Learning inverse folding from millions of predicted structures (2022), p. 2022.04.10.487779, , doi:10.1101/2022.04.10.487779.](https://www.zotero.org/google-docs/?5JaQi2)

[65. N. Chennamsetty, V. Voynov, V. Kayser, B. Helk, B. L. Trout, Design of therapeutic proteins with enhanced stability. *Proc. Natl. Acad. Sci.* **106**, 11937–11942 (2009).](https://www.zotero.org/google-docs/?5JaQi2)

[66. S. K. Jha, A. Ramanathan, R. Ewetz, A. Velasquez, S. Jha, Protein Folding Neural Networks Are Not Robust. *ArXiv210904460 Cs Q-Bio* (2021) (available at http://arxiv.org/abs/2109.04460).](https://www.zotero.org/google-docs/?5JaQi2)

[67. A. Ilyas, S. Santurkar, D. Tsipras, L. Engstrom, B. Tran, A. Madry, Adversarial Examples Are Not Bugs, They Are Features. *ArXiv190502175 Cs Stat* (2019) (available at http://arxiv.org/abs/1905.02175).](https://www.zotero.org/google-docs/?5JaQi2)

[68. A. Demontis, M. Melis, M. Pintor, M. Jagielski, B. Biggio, A. Oprea, C. Nita-Rotaru, F. Roli, Why Do Adversarial Attacks Transfer? Explaining Transferability of Evasion and Poisoning Attacks. *ArXiv180902861 Cs Stat* (2019) (available at http://arxiv.org/abs/1809.02861).](https://www.zotero.org/google-docs/?5JaQi2)

[69. B. Dang, M. Mravic, H. Hu, N. Schmidt, B. Mensa, W. F. DeGrado, SNAC-tag for sequence-specific chemical protein cleavage. *Nat. Methods*. **16**, 319–322 (2019).](https://www.zotero.org/google-docs/?5JaQi2)

[70. F. W. Studier, Protein production by auto-induction in high density shaking cultures. *Protein Expr. Purif.* **41**, 207–234 (2005).](https://www.zotero.org/google-docs/?5JaQi2)

[71. Y. Zhang, J. Skolnick, TM-align: a protein structure alignment algorithm based on the TM-score. *Nucleic Acids Res.* **33**, 2302–2309 (2005).](https://www.zotero.org/google-docs/?5JaQi2)

[72. A. Jaegle, F. Gimeno, A. Brock, A. Zisserman, O. Vinyals, J. Carreira, Perceiver: General Perception with Iterative Attention. *ArXiv210303206 Cs Eess* (2021) (available at http://arxiv.org/abs/2103.03206).](https://www.zotero.org/google-docs/?5JaQi2)

[73. W. Kabsch, A solution for the best rotation to relate two sets of vectors. *Acta Crystallogr. A*. **32**, 922–923 (1976).](https://www.zotero.org/google-docs/?5JaQi2)

[74. A. W. Senior, R. Evans, J. Jumper, J. Kirkpatrick, L. Sifre, T. Green, C. Qin, A. Žídek, A. W. R. Nelson, A. Bridgland, H. Penedones, S. Petersen, K. Simonyan, S. Crossan, P. Kohli, D. T. Jones, D. Silver, K. Kavukcuoglu, D. Hassabis, Improved protein structure prediction using potentials from deep learning. *Nature*, 1–5 (2020).](https://www.zotero.org/google-docs/?5JaQi2)

[75. R. F. Alford, A. Leaver-Fay, J. R. Jeliazkov, M. J. O’Meara, F. P. DiMaio, H. Park, M. V. Shapovalov, P. D. Renfrew, V. K. Mulligan, K. Kappel, J. W. Labonte, M. S. Pacella, R. Bonneau, P. Bradley, R. L. Dunbrack, R. Das, D. Baker, B. Kuhlman, T. Kortemme, J. J. Gray, The Rosetta All-Atom Energy Function for Macromolecular Modeling and Design. *J. Chem. Theory Comput.* **13**, 3031–3048 (2017).](https://www.zotero.org/google-docs/?5JaQi2)

[76. S. E. Boyken, Z. Chen, B. Groves, R. A. Langan, G. Oberdorfer, A. Ford, J. M. Gilmore, C. Xu, F. DiMaio, J. H. Pereira, B. Sankaran, G. Seelig, P. H. Zwart, D. Baker, De novo design of protein homo-oligomers with modular hydrogen-bond network--mediated specificity. *Science*. **352**, 680–687 (2016).](https://www.zotero.org/google-docs/?5JaQi2)

[77. D.-A. Silva, B. E. Correia, E. Procko, in *Computational Design of Ligand Binding Proteins*, B. L. Stoddard, Ed. (Springer, New York, NY, 2016; https://doi.org/10.1007/978-1-4939-3569-7_17), *Methods in Molecular Biology*, pp. 285–304.](https://www.zotero.org/google-docs/?5JaQi2)

[78. M. Steinegger, J. Söding, MMseqs2 enables sensitive protein sequence searching for the analysis of massive data sets. *Nat. Biotechnol.* **35**, 1026–1028 (2017).](https://www.zotero.org/google-docs/?5JaQi2)

[79. H. Park, P. Bradley, P. Greisen, Y. Liu, V. K. Mulligan, D. E. Kim, D. Baker, F. DiMaio, Simultaneous Optimization of Biomolecular Energy Functions on Features from Small Molecules and Macromolecules. *J. Chem. Theory Comput.* **12**, 6201–6212 (2016).](https://www.zotero.org/google-docs/?5JaQi2)

[80. V. Hornak, R. Abel, A. Okur, B. Strockbine, A. Roitberg, C. Simmerling, Comparison of multiple Amber force fields and development of improved protein backbone parameters. *Proteins Struct. Funct. Bioinforma.* **65**, 712–725 (2006).](https://www.zotero.org/google-docs/?5JaQi2)

[81. J. S. McLellan, M. Chen, A. Kim, Y. Yang, B. S. Graham, P. D. Kwong, Structural basis of respiratory syncytial virus neutralization by motavizumab. *Nat. Struct. Mol. Biol.* **17**, 248–250 (2010).](https://www.zotero.org/google-docs/?5JaQi2)

[82. J. Shang, G. Ye, K. Shi, Y. Wan, C. Luo, H. Aihara, Q. Geng, A. Auerbach, F. Li, Structural basis of receptor recognition by SARS-CoV-2. *Nature*. **581**, 221–224 (2020).](https://www.zotero.org/google-docs/?5JaQi2)

[83. J. L. Fallon, F. A. Quiocho, A Closed Compact Structure of Native Ca2+-Calmodulin. *Structure*. **11**, 1303–1307 (2003).](https://www.zotero.org/google-docs/?5JaQi2)

[84. C. U. Kim, H. Song, B. S. Avvaru, S. M. Gruner, S. Park, R. McKenna, Tracking solvent and protein movement during CO2 release in carbonic anhydrase II crystals. *Proc. Natl. Acad. Sci.* **113**, 5257–5262 (2016).](https://www.zotero.org/google-docs/?5JaQi2)

[85. P. H. Kussie, S. Gorina, V. Marechal, B. Elenbaas, J. Moreau, A. J. Levine, N. P. Pavletich, Structure of the MDM2 Oncoprotein Bound to the p53 Tumor Suppressor Transactivation Domain. *Sci. New Ser.* **274**, 948–953 (1996).](https://www.zotero.org/google-docs/?5JaQi2)

[86. S. F. Altschul, W. Gish, W. Miller, E. W. Myers, D. J. Lipman, Basic local alignment search tool. *J. Mol. Biol.* **215**, 403–410 (1990).](https://www.zotero.org/google-docs/?5JaQi2)

### 
